# A chemical reaction controlled by light-activated molecular switches based on hetero-cyclopentanediyls†
†Electronic supplementary information (ESI) available: Experimental information (incl. experimental setups), structure elucidation, syntheses, spectroscopic details and computational details. CCDC 1869014 and 1869015. For ESI and crystallographic data in CIF or other electronic format see DOI: 10.1039/c8sc04893b


**DOI:** 10.1039/c8sc04893b

**Published:** 2019-02-18

**Authors:** Jonas Bresien, Thomas Kröger-Badge, Stefan Lochbrunner, Dirk Michalik, Henrik Müller, Axel Schulz, Edgar Zander

**Affiliations:** a Institute of Chemistry , University of Rostock , Albert-Einstein-Str. 3a , D-18059 Rostock , Germany . Email: jonas.bresien@uni-rostock.de ; Email: axel.schulz@uni-rostock.de; b Institute of Physics , University of Rostock , Albert-Einstein-Str. 23-24 , D-18059 Rostock , Germany; c Department of Life, Light & Matter , University of Rostock , D-18051 Rostock , Germany; d Leibniz Institute for Catalysis at the University of Rostock e.V. , Albert-Einstein-Straße 29a , D-18059 Rostock , Germany

## Abstract

Molecular switches are molecules that can reversibly be shifted between at least two stable states with different physical and chemical properties, making them interesting for application as chemical sensors or molecular machines. We recently discovered that five-membered, cyclic biradicals based on group 15 elements are efficient and robust photochemical switches that can be activated by red light. The quantum yield of the photo-isomerization is as high as 24.6%, and the thermal equilibration of the photo-activation product proceeds rapidly at ambient temperature. The fully reversible process was studied by experimental and high-level *ab initio* techniques. We could further demonstrate that the biradical character could be completely turned on and off, so the system could be applied to control chemical equilibria that involve activation products of the cyclic biradicals.

## Abstract 2

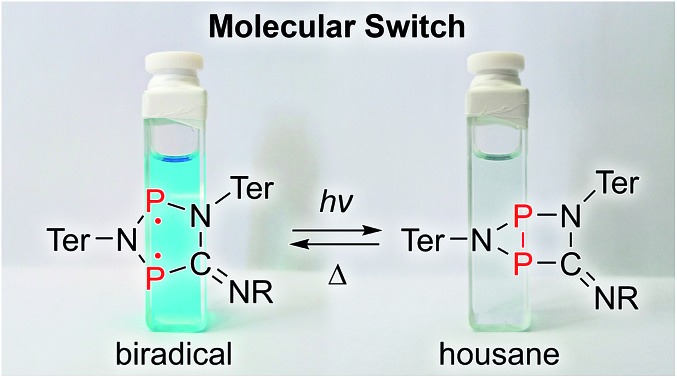
Biradicals were applied as molecular switches to control chemical reactions that involve the activation of small molecules. The mechanism was studied by experimental and computational methods.

## Introduction

The term “molecular switch” originated in the early 1980s and was first used in conjunction with biological signal transmission.^1^ Since then, it has found widespread use throughout chemistry, and generally refers to any kind of molecule that can exist in two or more (*meta*-)stable states. Interconversion can be induced by external stimuli such as temperature, chemical modifications, redox reactions, electric fields, or irradiation with photons.^2^–^5^ The most commonly known type of molecular switches are pH indicators, which are based on acid–base equilibria and therefore belong to the group of chemically activated compounds.^6^,^7^ Other chemically switchable systems may, for example, find application in the field of gas or ion sensing,^5^,^8^ or be used as redox indicators.^7^

**Fig. 1:**
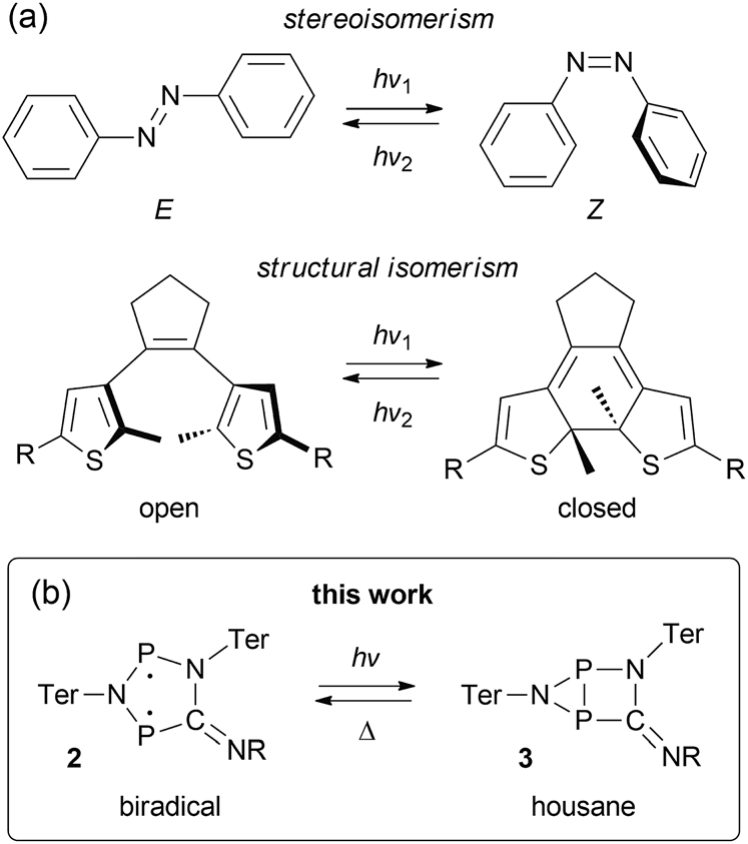
Types of molecular switches. (a) Azobenzenes (top) and stilbenes can be categorized as stereoisomeric switches, while diarylethenes (bottom), spiropyranes, spiroxazines, and fulgimides are typical examples of structural isomeric switches. (b) We investigated the photo-switching behavior of hetero-cyclopentane-1,3-diyls (**2**), which display considerable singlet biradical character (R = ^*t*^Bu, Dmp (2,6-dimethylphenyl), Ter = 2,6-bis(2,4,6-trimethylphenyl)phenyl).

Especially light-activated molecular switches promise diverse applications, *e.g.* as photoactuators^9^–^11^ or photo-regulated catalysts.^12^,^13^ Therefore, a growing research interest is aimed both at the synthesis of new systems as well as the exploration of new fields of applications.^14^,^15^ Most light-activated molecular switches function by conformational changes.^3^ One of the most prominent examples is probably retinal, the chromophore of the receptor protein rhodopsin, which is found in the rods of the retina in mammals' eyes. Upon irradiation with visible light, retinal undergoes a conformational change resulting in a signalling cascade, which eventually leads to a visual stimulus.^16^–^18^ Therefore, it plays a crucial role in the functioning of the eye. Mechanistically, the isomerization of retinal can be traced back to an *E*/*Z* isomerization along a C–C double bond. Thus, it is not surprising that many synthetic, light activated molecular switches are based on a similar principle:^19^ For example, azobenzene may be isomerized from its *E* to its *Z* conformer by light in the UV region, while the *Z* → *E* isomerization is triggered by light in the blue region of the visible spectrum (Fig. 1a).^20^,^21^ The absorption maxima can be shifted by derivatization of azobenzene with electron donating or electron withdrawing groups.^4^ Another popular type of photo-switching is represented by photo-cyclization reactions,^22^,^23^ as observed, for example, in case of diarylethenes (Fig. 1a).^24^ This class of molecular switches is based on the formation or breaking of chemical bonds. In fact, most (light-activated) molecular switches can be divided into these two categories, namely stereoisomerism (change of structure without modification of the chemical bonding pattern) and structural isomerism (including a redistribution of bonds).^3^ Other mechanisms are discussed in the literature, such as dipole, charge, or spin switching,^3^ but these processes often include some kind of structural change, too.

Understanding bond formation and breaking processes (or the nature of chemical bonds in general) has been a widely investigated topic throughout all areas of chemical science.^25^–^28^ Amongst others, singlet biradicals (or biradicaloids) are frequently cited as suitable model systems for bond formation and breaking processes due to their peculiar electronic structure, which is sometimes referred to as comprising a “partial bond” between the formal radical centres.^29^ In this regard, singlet biradicals are promising candidates for molecular switches, as small structural changes should generally alter the bonding interaction between the radical centres significantly, thereby also affecting the biradical character. The latter being the source of many interesting properties, such as small-molecule activation^30^–^33^ or non-linear optical properties,^29^,^34^ it should be possible to switch these characteristics as well. Still, there are only few experimental examples in the literature taking advantage of such an approach.^35^–^38^

We recently introduced stable hetero-cyclopentane-1,3-diyls based on group 15 elements (**2**, Fig. 1b).^39^–^42^ These five-membered cyclic biradicals can be used to activate small molecules such as alkynes, carbon monoxide or isonitriles. Furthermore, preliminary investigations indicated that they can be isomerized by light to a closed-shell housane-type isomer (**3**) featuring a P–P single bond. Contrary to carbon based cyclopentane-1,3-diyls,^43^–^46^ the isomerization was shown to be reversible. Since we are not aware of molecular switches based on this class of compounds, we sought to study the photo-isomerization in more detail, so as to understand the mechanism of the photo-switching process and control the activation chemistry by switching the biradical character on and off.

## Results and discussion

### Photo-isomerization and thermal reverse reaction

**Fig. 2:**
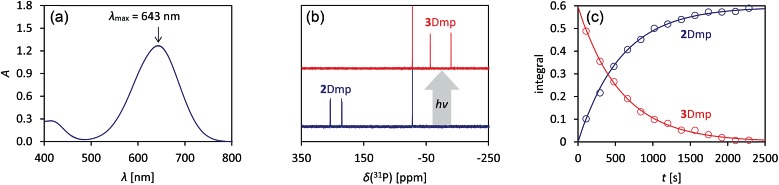
Photo-isomerization of **2Dmp** and thermal equilibration of **3Dmp** at 25 °C. (a) UV-Vis spectrum of **2Dmp**. (b) ^31^P NMR spectra of a solution of **2Dmp** in the dark (bottom) and under irradiation (top). (c) Thermal reverse reaction (**3Dmp** → **2Dmp**) monitored by ^31^P NMR spectroscopy (please see ESI† for data at different temperatures).

**Fig. 3:**
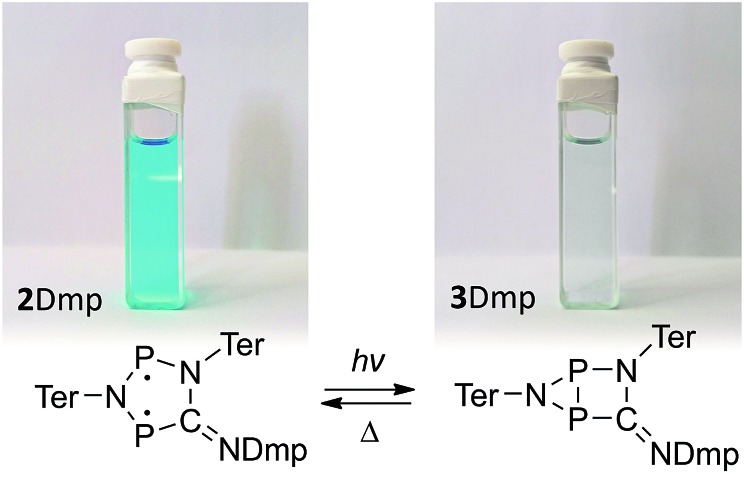
Left: blue, dilute solution of **2Dmp** in benzene. Right: colourless solution of the photo-product **3Dmp** in benzene.

We chose to begin our investigations on the system **2Dmp** (Fig. 1b, R = Dmp = 2,6-dimethylphenyl), since it had proved to be the most stable derivative.^40^ It was synthesized from the biradical [P(μ-NTer)]_2_ (**1**, Ter = 2,6-dimesitylphenyl) by insertion of DmpNC according to published procedures (*cf.* ESI,†). The UV-Vis spectrum exhibited a broad absorption band centred at 643 nm, *i.e.* in the red region of the visible spectrum (Fig. 2a). According to time-dependent density functional theory (TD-DFT), complete active space self-consistent field (CASSCF), and model multi-reference configuration interaction (MRCI) calculations, the absorption may be attributed to the formal HOMO → LUMO transition (*vide infra*; for detailed information on all calculations and proper electronic description of the singlet biradical please refer to the ESI, p. S40†). Since the “LUMO” is transannularly bonding in character between the two P atoms (Fig. S28 and S29;† *cf.*Fig. 7b), it seemed plausible that population of this orbital due to electronic excitation would lead to P–P bond formation and therefore to isomerization to the housane **3Dmp**. Accordingly, irradiation of a blue, dilute solution of **2Dmp** with red light (600–650 nm) resulted in quick discoloration (Fig. 3), and the absorption band at 643 nm vanished. When the irradiation was discontinued, the blue solution was fully recovered within 30 minutes (25 °C), as evidenced by UV-Vis measurements (Fig. S18†).

**Fig. 4:**
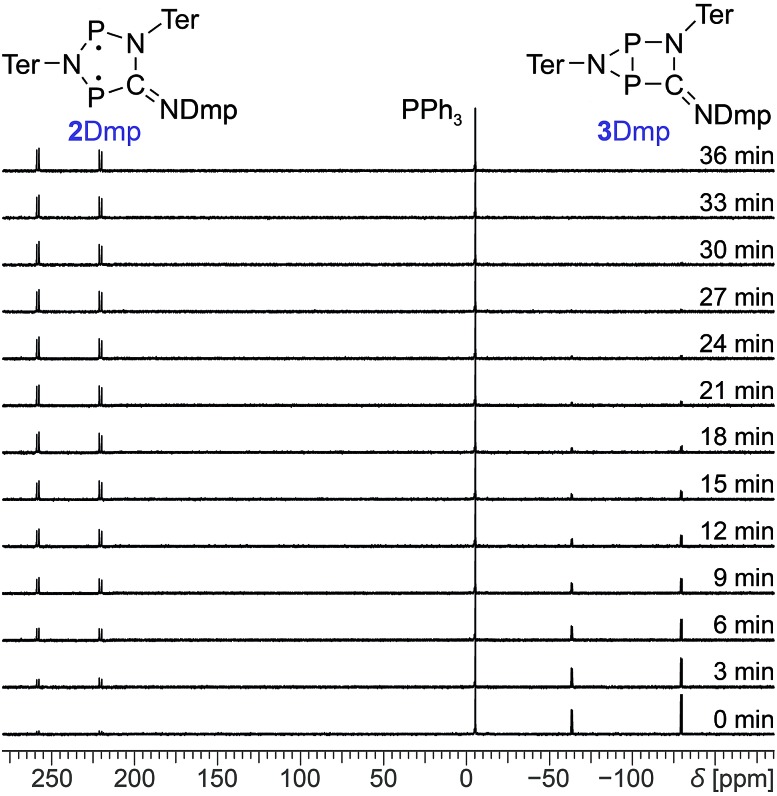
Time-dependent ^31^P NMR spectra of the thermal reverse reaction (**3Dmp** → **2Dmp**). PPh_3_ was added as internal standard.

Since ^31^P NMR spectroscopy is exceptionally well suited to study molecules containing P atoms, we decided to irradiate our samples directly in the spectrometer, so we could trace the isomerization reactions as well as determine yields, reaction rates, the stability of the system and possible by-products. We adopted a setup previously published by the Gschwind group,^47^ using a laser diode instead of an LED as light source. The diode was coupled with an optical fibre, which was inserted into an NMR tube equipped with a coaxial insert, allowing us to measure spectra under light (for technical details, see ESI†). Indeed, irradiation of **2Dmp** with red light (638 nm) led to full conversion of the biradical **2Dmp** to the housane **3Dmp** (Fig. 2b). When the diode was switched off, the thermal equilibration could be monitored (Fig. 4). The thermal reverse reaction was found to be a first-order reaction with a half-life of about 7 min at ambient temperature (*k* = 1.73(3) × 10^–3^ s^–1^), leading to quantitative recovery of **2Dmp** (Fig. 2c). The activation barrier was determined according to the Eyring theory by re-determination of the rate constants at several temperatures, giving a Gibbs free energy of activation of 88(4) kJ mol^–1^ at 25 °C (*cf.* p. S25†), in good agreement with theoretical predictions (ESI†). No signals of side products were observed in the ^31^P NMR spectrum after several switching cycles. In a sealed tube, a solution of **2Dmp** in benzene was stable for more than a year, and it did not lose its switching capacity.

### Solid-state properties

**Fig. 5:**
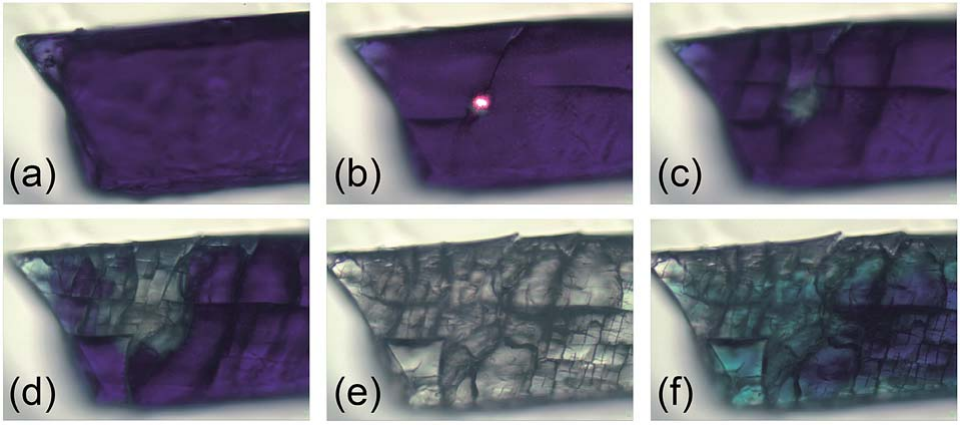
Photos of a single crystal of **2Dmp** at several stages of irradiation (25 °C). The cracks in the crystal due to structural changes at the molecular level can be nicely seen. (a) Before irradiation. (b) During irradiation with red laser (633 nm, 0.1 mW). (c) Immediately after irradiation for 1 min (0.1 mW). (d) Immediately after irradiation for 5 min (0.1 mW). (e) Immediately after irradiation for 5 min (10 mW). (f) 10 min after irradiation (reverse reaction is not yet complete).

Next, we investigated the solid-state properties of the molecular switch **2Dmp**. Upon irradiation of a dark blue crystal with a red HeNe laser (633 nm, 0.1 mW), several cracks emanating from a circular, colourless spot could be observed (Fig. 5a–c). Increasing the light intensity (10 mW) led to complete discoloration, but also to fragmentation of the crystal (Fig. 5d and e). We attributed this observation to significant structural changes at the molecular level (*vide infra*) that caused stress within the crystal lattice and ultimately led to cracking of the crystal. When the light was switched off, a blue, polycrystalline material was recovered (Fig. 5f) that could be identified as **2Dmp** by Raman spectroscopy (*vide infra*).

Notably, ambient light did not affect the integrity of single crystals at room temperature. However, at low temperatures such as –80 °C, even low intensity (white) light eventually led to their disintegration. This can be understood in terms of the thermal reverse reaction: at ambient temperature, the rate of thermal equilibration is much higher than the rate of photo-conversion from ambient light, so the light is completely absorbed at the surface of the crystal. At low temperatures, though, the thermal reverse reaction is much slower than the rate of photo-conversion. Hence, given time, a critical number of molecules will have isomerized at some point, resulting in cracking of the crystal.

**Fig. 6:**
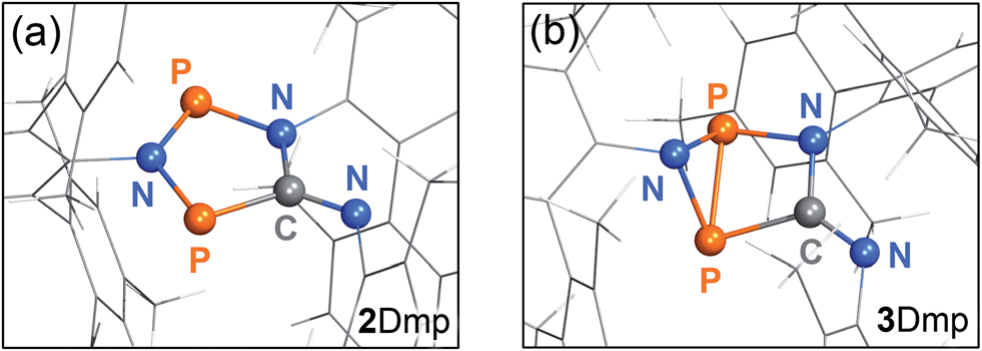
(a) Structure of the planar P_2_N_3_C scaffold of **2Dmp** as determined by single crystal X-ray diffraction. (b) Calculated structure of **3Dmp** with a central hetero-bicyclo[2.1.0]pentane motif.

With this knowledge, we could now determine the crystal structure of **2Dmp**. Notably, the crystal was mounted at *ambient* temperature, and then cooled to –150 °C for measurement in complete darkness. (All previous attempts to obtain a complete data set failed since the crystals were mounted at low temperatures, leading to their disintegration due to irradiation by the mounting lamp.)^40^ The molecular structure comprises a nearly planar, five-membered P_2_N_2_C ring system (Fig. 6a). All P–N, P–C, and N–C bond lengths within the ring range between typical values^48^ of a single and a double bond. The transannular P···P distance (2.9437(7) Å) is significantly longer than a P–P single bond (Σ*r*_cov_ = 2.22 Å),^48^ in agreement with the biradical character of the compound (*cf.* p. S46†).

So far, all our efforts to obtain single crystals of the housane **3Dmp** have failed. Crystallization under light led to a colourless solid; however, the crystals were too small for single-crystal X-ray analysis. Nonetheless, we performed dispersion corrected^49^ density functional theory (DFT-D3) calculations to predict the structure (Fig. 6b). Contrary to **2Dmp**, the five-membered P_2_N_2_C ring system of **3Dmp** adopts an envelope conformation with a transannular P–P single bond (2.220 Å). Therefore, the structure changes significantly upon isomerization, explaining the macroscopic changes observed in the material under irradiation.

The isomerization in the solid state could also be observed by Raman microscopy. Apart from visual changes of the material, the Raman spectrum changed significantly after irradiation. Especially, the C–N stretch of the exocyclic C=N double bond can be utilized as a probe to identify the isomers (*ν̃*_CN_ = 1640 cm^–1^ in **3Dmp**, Fig. S25†).

### Mechanism

Comprehensive computations were carried out to understand the mechanism of the photo-isomerization as well as the thermal reverse reaction. Since the correct quantum-mechanical treatment of biradicals requires multi-reference wave functions (which severely limits the size of systems that can be investigated), we opted to study the isomerization process using a model system bearing only hydrogen substituents (**2H**). Various calculations were performed to ascertain that this is indeed a suitable model (ESI†).

**Fig. 7:**
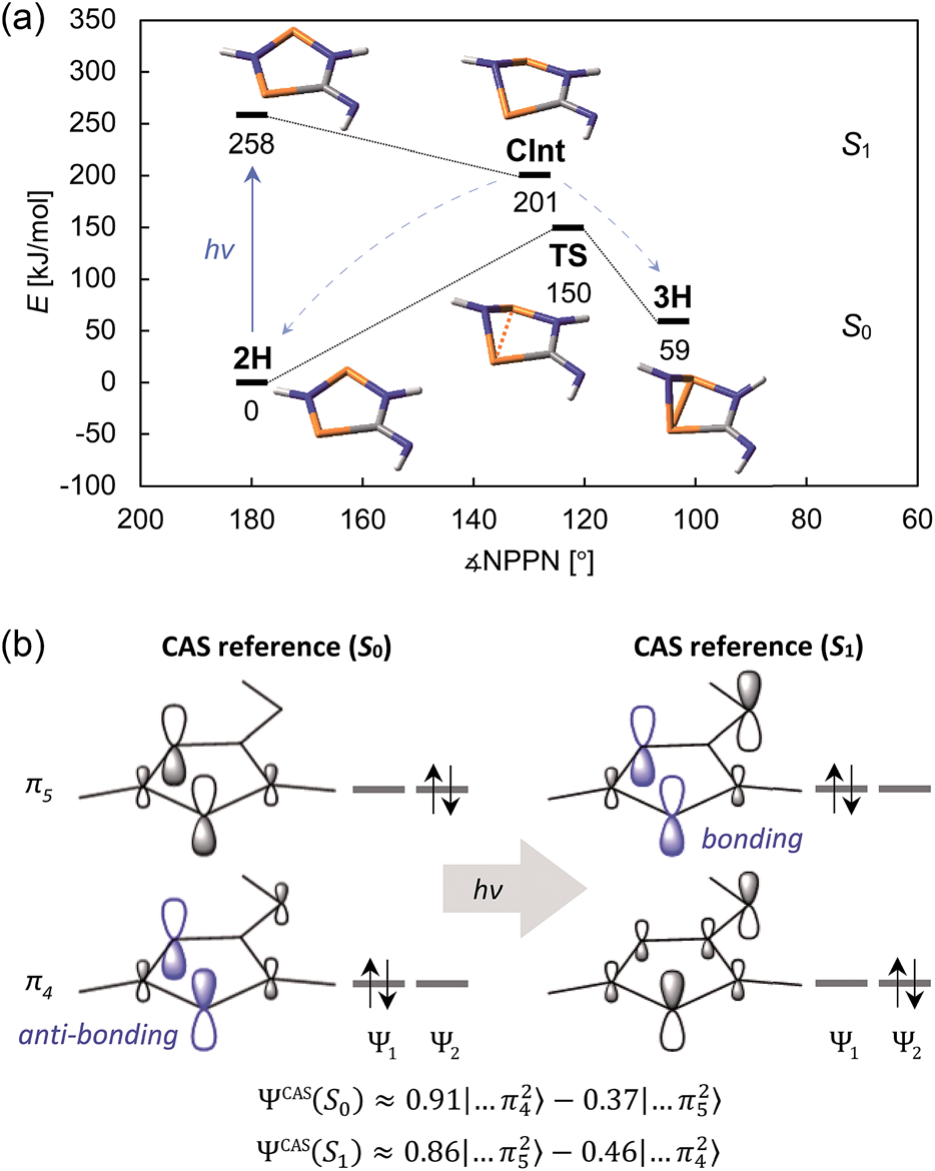
(a) Schematic view of the ground state and first excited state PES of the model system **2H**. (b) Simplified depiction of the relevant orbitals of a state-specific CAS (8, 6) calculation. Upon excitation, the electron density is redistributed, resulting in a mostly bonding interaction between the two P atoms. The approximate wave functions are given by *Ψ*^CAS^ ≈ *c*_1_*Ψ*_1_ + *c*_2_*Ψ*_2_.

The CASSCF and MRCI results indicated that the electronic excitation of **2H** leads to a non-stationary point on the *S*_1_ potential energy surface (PES). Distortion of the molecule – mainly by folding of the five-membered ring along the P···P axis and concurrent shortening of the P···P interatomic distance – leads to a conical intersection (CInt) between the *S*_1_ and *S*_0_ surface, therefore allowing radiationless deactivation of the excited state. The CInt is in the vicinity of a transition state on the *S*_0_ PES which connects the biradical **2H** and the housane **3H**, enabling thermal isomerization of **3H** to **2H** (Fig. 7a; for more details on the calculations, see p. S42†).

As indicated, a qualitatively correct wave function of a biradical requires multiple determinants. The same is true of excited states, at least if they are modelled as higher energy roots of the CI matrix. However, depending on the reference orbitals used for the CI, the resulting wave functions may be rather difficult to interpret. The more qualitative CASSCF approach (which neglects dynamic electron correlation) is probably best suited to understand the electronic structures of **2H** in its ground and first excited singlet state. In particular, a state-specific CASSCF calculation yields a unique set of orbitals for each state. These orbitals are variationally optimized to best represent the electron distribution for a given state, *i.e.* fewer determinants are needed to describe the wave function, making it easier to interpret. The results for **2H** nicely demonstrate that both the *S*_0_ and *S*_1_ state have considerable biradical character^50^ (28 and 44%, respectively; *cf.* 27 and 36% for **2Dmp**). The dominant contribution to the wave function of the ground state is transannularly anti-bonding between the two P atoms, whereas the dominant contribution to the *S*_1_ state is bonding in nature, in agreement with earlier considerations (Fig. 7b). Note that the CASSCF results also correctly indicate that the electronic excitation involves redistribution of the whole electron density, rather than just one electron being excited into a vacant orbital. A rather detailed discussion of the electronic wave function and electronic excitation including MRCI results can be found in the ESI (p. S46†).

### Quantum yield

As demonstrated by the calculations, the excited state can deactivate through a conical intersection. However, this process can lead to the formation of both the starting material (**2Dmp**) or the photo-product (**3Dmp**). Thus, we were interested in the quantum yield of the photo-isomerization, *i.e.* the ratio of successful isomerizations to absorbed photons. Taking advantage of the different absorbances of **2Dmp** and **3Dmp**, we irradiated a dilute solution with a red HeNe laser (633 nm) and measured the transmitted laser intensity behind the sample. Due to photo-isomerization, the transmitted intensity increased over time, and the time-resolved data could be used to determine the quantum yield (p. S34†). With a value of 24.6(8)%, the photo-isomerization is rather efficient (*cf.* photosynthesis in plants: 5–12%;^51^–^53^ azobenzene: 11% (*trans* → *cis*), 42% (*cis* → *trans*);^54^ diarylethenes: 13–59% (cyclization), 0.002–59% (cycloreversion) depending on the substituents;^55^ retinal: 65% 56).

### Manipulation of an equilibrium reaction

Having understood the photo-switching of the biradical **2Dmp**, we were now interested whether it might be possible to manipulate a chemical reaction using this type of molecular switch. In particular, an equilibrium reaction involving activation chemistry of the biradical **2** seemed promising, as this would open the possibility to couple thermal and photochemical processes.

**Scheme 1:**
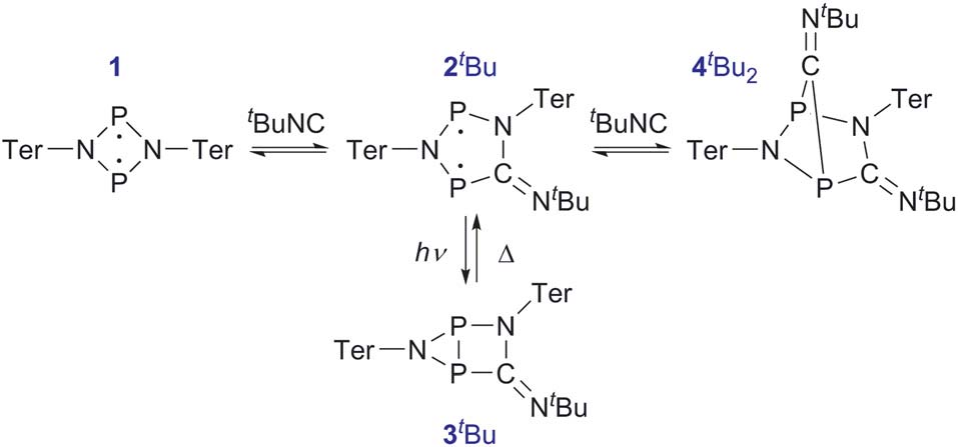
The thermal equilibrium can be completely suppressed by irradiation.

**Fig. 8:**
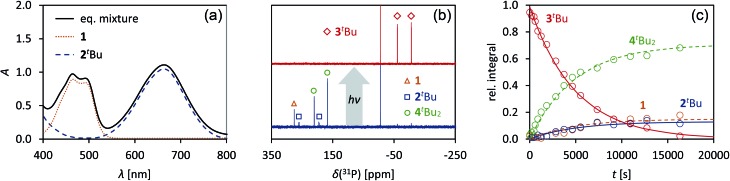
(a) UV-Vis spectrum of an equilibrium mixture of **1**, **2^*t*^Bu**, **4^*t*^Bu_2_**, and ^*t*^BuNC. (b) Upon irradiation with red light, the complete equilibrium is suppressed and the housane **3^*t*^Bu** is formed exclusively, as evidenced by ^31^P NMR spectroscopy. (c) The thermal reverse reaction to recover the equilibrium species proceeds as first-order reaction.

As previously shown, the reaction of **1** with ^*t*^BuNC did not lead to quantitative formation of **2^*t*^Bu** but rather to an equilibrium mixture of **1**, **2^*t*^Bu**, and **4^*t*^Bu_2_**, the addition product of the biradical **2^*t*^Bu** and ^*t*^BuNC (Scheme 1). The colourless adduct **4^*t*^Bu_2_** could be isolated from the mixture by crystallization (ESI†).^40^ When re-dissolving the colourless crystals, the blue-green equilibrium mixture of **1**, **2^*t*^Bu**, and **4^*t*^Bu_2_** was recovered, as evidenced by ^31^P NMR experiments. The UV-Vis spectrum of the solution (Fig. 8a) showed a broad absorption band at 663 nm (**2^*t*^Bu**) and two absorption maxima at 463 and 491 nm (**1**). Accordingly, irradiation of the solution with a red laser diode (638 nm) led to photo-isomerization of the molecular switch **2^*t*^Bu**. Thus, it was completely removed from the thermal equilibrium, resulting in quantitative formation of the housane **3^*t*^Bu** and disappearance of the thermal products **1** and **4^*t*^Bu_2_** (Fig. 8b); *i.e.* the thermal equilibrium involving the activation chemistry of the biradical was entirely suppressed by the photo-isomerization! This might be regarded as “quenching of the biradical character”, as previously proposed by Wang *et al.*^34^

When the light source was switched off, the thermodynamic equilibrium mixture was slowly recovered. The housane **3^*t*^Bu** decayed in a first order reaction with a half-life of about 57 min at 25 °C (*k* = 2.03(4) × 10^–4^ s^–1^, Fig. 8c). The process was completely reversible, and even after several switching cycles, no side products could be observed in the ^31^P NMR spectrum.

**Scheme 2:**
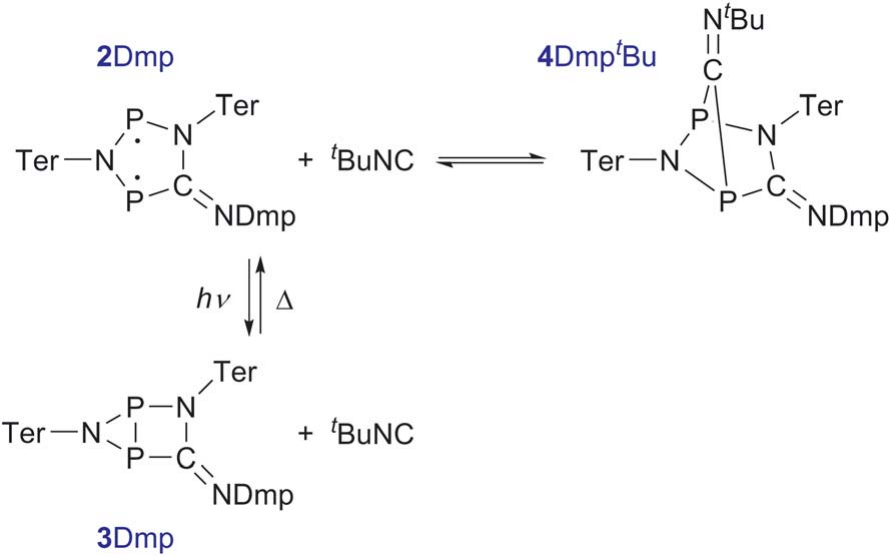
Reaction of **2Dmp** with ^*t*^BuNC.

Finally, the activation of ^*t*^BuNC with the more stable biradical **2Dmp** was investigated. Indeed, the expected adduct **4Dmp^*t*^Bu** was formed in an equilibrium with **2Dmp** (Scheme 2). To our surprise, irradiation of the blue solution with a red laser diode (638 nm) did not lead to discoloration as in the previous case. Even though formation of the photo-product **3Dmp** was observed in the ^31^P NMR spectrum, quantitative conversion could not be achieved at ambient temperature, indicating that the rate of thermal equilibration was on the same order of magnitude as the rate of photo-conversion and therefore much faster than previously observed (*cf. t*_1/2_ ≈ 7 min in the absence of ^*t*^BuNC). In fact, as soon as the irradiation was discontinued, the thermal equilibrium was restored. Low-temperature investigations indicated that the ^*t*^BuNC acted as a catalytic agent to accelerate the thermal reverse reaction of the housane **3Dmp** (p. S31†). The mechanism of the catalyzed reverse reaction has yet to be investigated; this is outside the scope of this article and will therefore be explored in the future. For now, we just want to point out that it is possible to chemically manipulate the pathway of the thermal reverse reaction and thus influence the switching behaviour.

## Conclusion

We could demonstrate that biradicals based on hetero-cyclopentane-1,3-diyls (**2**) are potent and chemically robust systems for application as molecular switches. They could be isomerized by red light to a closed-shell housane-type species (**3**) with a transannular bond, effectively quenching the biradical character. The biradical (**2**) was then fully recovered by a thermal reverse reaction in darkness. Substitution of the organic groups at the exocyclic C=N scaffold changed the properties of the systems, promising variability of key features such as stability, activation chemistry, and excitation wavelength.

The isomerization could be monitored by UV-Vis, NMR, and Raman spectroscopy. In the solid state, significant structural changes at the molecular level upon irradiation resulted in macroscopic changes such as fragmentation of crystals. Hence, even though the molecular process was fully reversible, the macroscopic crystallinity was lost upon isomerization. In solution, the switching process could be repeated many times without any indications of decomposition. While the rate of photo-isomerization depended on the light intensity, the rate of thermal reverse reaction could be influenced either by changes in temperature or chemically by addition of isonitriles.

In case of the ^*t*^Bu derivative **2^*t*^Bu**, it was possible to completely suppress the thermal equilibrium related to the activation chemistry of the biradical upon irradiation. This serves as a proof-of-principle that chemical reactions can be controlled by molecular switches that take advantage of kinetically stabilized biradicals.

The mechanisms of both photo- and thermal isomerization were studied in detail using multi-reference calculations as well as experimental data. Formal one-electron excitation of **2** led to a transannularly bonding interaction and thus distortion of the molecule on the *S*_1_ PES. Deactivation proceeded through a conical intersection and hence radiationlessly. The quantum yield of the photochemical isomerization was determined to be 24.6(8)%. The thermal reverse reaction (**3** → **2**) proceeded through a single transition state on the ground state PES and was found to be a first-order reaction.

In future studies, we will further investigate the scope of hetero-cyclopentane-1,3-diyls as molecular switches, especially with respect to the control of chemical reactions, such as [2 + 2] additions of alkynes and alkenes.

## Experimental

For detailed information on syntheses, equipment, analytical data, computational methods *etc.* please also consult the ESI.†

All manipulations were carried out under oxygen- and moisture-free conditions under an inert atmosphere of argon using standard Schlenk or dry box techniques.

### NMR spectroscopy under irradiation

All irradiation experiments were performed on a Bruker AVANCE 250 MHz spectrometer. Light was directed into the sample using a multimode optical fibre with 1000 μm core diameter. To ensure inert conditions, the NMR tubes were filled in a glove box and equipped with a conical insert that could host the optical fibre. Thus, the sample volume could be kept isolated from the outside atmosphere. As light source we used a 700 mW laser diode (638 nm), which was mounted on the far end of the optical fibre. The outer cladding of the fibre was removed for about 5 cm on the sample side and the glass surface was roughened to ensure uniform irradiation of the sample, as previously reported.^47^

### UV-Vis spectroscopy

UV-Vis spectra were recorded on a Perkin-Elmer Lambda 19 UV-Vis spectrometer. For irradiation studies, the samples were irradiated outside the spectrometer using white or monochromated light (600–650 nm).

### Determination of the quantum yield

We used a self-constructed setup mounted on a standard optical table (*cf.* ESI†). The samples were prepared in a glove box and filled in gas-tight cuvettes with an optical path length of 1 cm. The solutions were irradiated with a HeNe laser (633 nm) and the transmitted laser intensity was measured using a photo diode. The laser intensity was chosen so high that the rate of photo conversion was much larger than the rate of thermal equilibration; hence, the reverse reaction could be neglected. We derived the following time law for the signal sig(*t*) measured at the photo diode:
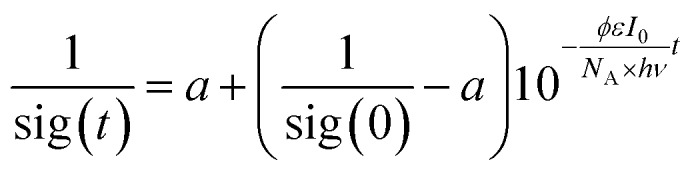
 *φ* is the quantum yield, *ε* the molar extinction coefficient, *I*_0_ the incident laser intensity, *N*_A_ Avogadro's number, *hν* the energy of a photon, and *a* a fitting parameter related to the detection sensitivity. For a derivation of this formula and detailed description of the experimental setup, please refer to the ESI.†

Two different concentrations of **2Dmp** in benzene were prepared. The samples were irradiated for 15 seconds, and each of those measurements was repeated multiple times. The data for each of the two concentrations were combined and evaluated by non-linear fitting procedures.

### Computational methods

Electronic structure computations were performed using Gaussian09 57 (DFT, CASSCF) or ORCA 4.0.1 58 (CASSCF, MRCI). Structure optimizations were run using pure density functionals in conjunction with the D3BJ^49^ dispersion correction. The investigation of the PES of the model system was done using CAS(8,6) computations, including all π-type orbitals of **2H** (*i.e.* those belonging to the *A*′′ irrep, point group *C*_S_).

To obtain an idea of the reliability of DFT methods to predict experimental properties of the biradical systems **1** and **2**, we performed a survey of different methods and compared their results to experimental as well as high-level *ab initio* data. Generally, DFT methods are suitable to predict molecular structures and first excitation energies with reasonable accuracy; however, the absolute KS energies are notoriously bad due to single-determinantal treatment of the KS wave function. Notably, the PBE functional^59^ outperformed all other methods at predicting NMR data. Please refer to the ESI† for in-depth information.

## Conflicts of interest

There are no conflicts to declare.

## Supplementary Material

Supplementary information

Crystal structure data
