# Supplementary material for: A chemical reaction controlled by light-activated molecular switches based on hetero-cyclopentanediyls
Source: Chem Sci. 2019 Feb 18;10(12):3486–93. doi: 10.1039/c8sc04893b (PMC6430090; doi:10.1039/c8sc04893b)
Supplement: Supplementary file 1 [file SC-010-C8SC04893B-s001.pdf]

Electronic Supplementary Material (ESI) for Chemical Science.
This journal is © The Royal Society of Chemistry 2019

SUPPLEMENTARY INFORMATION

A chemical reaction controlled by light-activated molecular switches based on hetero-cyclopentanediyls

Jonas Bresien, Thomas Kröger-Badge, Stefan Lochbrunner, Dirk Michalik, Henrik Müller, Axel Schulz,* and Edgar Zander*

This file includes:

1 Experimental ... 3
2 Structure elucidation ... 5
3 Syntheses of starting materials ... 8
3.1 Synthesis of DmpNC ... 8
3.2 Synthesis of TerN(H)PCl_2 ... 9
3.3 Synthesis of [ClP(μ-NTer)$_2$] ... 10
3.4 Synthesis of [P(μ-NTer)]$_2$ (**1**) ... 11
4 Syntheses of compounds ... 13
4.1 Synthesis of the biradical {[PN(Ter)]$_2$(μ-CNDmp)} (**2**Dmp) ... 13
4.2 Generation of the housane {[PN(Ter)]$_2$(μ-CNDmp)} (**3**Dmp) ... 14
4.3 Synthesis of {[PN(Ter)]$_2$(μ-CN^{t}Bu)$_2$} (**4**^{t}Bu$_2$) ... 15
4.4 Generation of the housane {[PN(Ter)]$_2$(μ-CN^{t}Bu)} (**3**^{t}Bu) ... 16
4.5 Synthesis of {[PN(Ter)]$_2$(μ-CN^{t}Bu)$_2$} (**4**Dmp^{t}Bu) ... 16
5 Additional spectroscopic details ... 18

5.1 UV-Vis spectra, extinction coefficient ... 18
5.2 NMR spectroscopy under irradiation ... 20
5.3 Photoisomerization ... 23
5.4 Thermal equilibration ... 25
5.5 Determination of the quantum yield of the photoisomerization ... 34
5.6 Raman microscopy ... 38
5.7 Fluorescence spectroscopy ... 39
6 Computational details ... 40
6.1 Prediction of experimental properties using DFT ... 40
6.2 Model system ... 42
6.3 Biradical character ... 46
6.4 Electronic structure of S_0 and S_1 state ... 47
6.5 NMR data ... 49
6.6 Optimized structures (.xyz files) ... 50
7 References ... 68

1 Experimental

General Information. If not stated otherwise, all manipulations were carried out under oxygen- and moisture-free conditions under an inert atmosphere of argon using standard Schlenk or Drybox techniques.

Table S1: Origin and purification of solvents and reactants.

Substance	Origin	Purification
CH_2Cl_2	local trade	purified according to literature procedure[1]<br>dried over P_4O_{10}, stored over CaH_2<br>freshly distilled and degassed (freeze-pump-thaw)
Et_2O, THF	local trade	dried over Na/benzophenone<br>freshly distilled prior to use
n-pentane, *n*-hexane	local trade	dried over Na/benzophenone/tetraglyme<br>freshly distilled prior to use
PhF	local trade	dried over CaH_2<br>freshly distilled prior to use
$HCCl_3$	local trade	used as received
CD_2Cl_2	euriso-top	dried over P_4O_{10} and CaH_2<br>freshly distilled prior to use
C_6D_6	euriso-top	dried over Na<br>freshly distilled prior to use
THF-d_8	eursio-top	dried over Na<br>distilled and stored over molecular sieves (4 Å)
PCl_3	Merck, for synthesis	dried over P_4O_{10}<br>freshly distilled and degassed (freeze-pump-thaw)
NEt_3	Sigma Aldrich, 99 %	dried over Na<br>freshly distilled prior to use
DBU	Merck, for synthesis	distilled
^{t}BuNC	old stock	distilled
$DmpNH_2$	Acros, 99 %	used as received
$[NE_4]Br$	Fluka	used as received
NaOH	Chemsolute, 98.8 %	used as received
Mg	abcr, 99.8 %, for Grignards	used as received
PPh_3	old stock	sublimed
$TerNH_2$	synthesized[2,3]	re-crystallized as described in the literature

NMR spectra were recorded on Bruker spectrometers AVANCE 250 and AVANCE 300 and were referenced internally to the deuterated solvent (^{13}C: CD_2Cl_2 δ_{ref} = 54.0 ppm, C_6D_6 δ_{ref} = 128.4 ppm, THF-d_8 $\delta_{ref,1}$ = 25.4 ppm, $\delta_{ref,2}$ = 67.6 ppm), to protic impurities in the deuterated solvent (^{1}H: $CHDCl_2$ δ_{ref} = 5.32 ppm, C_6HD_5 δ_{ref} = 7.16 ppm, THF-d_7 $\delta_{ref,1}$ = 1.73 ppm, $\delta_{ref,2}$ = 3.58 ppm) or externally (^{31}P: 85% H_3PO_4 δ_{ref} = 0 ppm). All measurements were carried out at ambient temperature unless denoted otherwise. NMR signals were assigned using experimental data (e.g. chemical shifts, coupling constants, integrals where applicable) in conjunction with computed NMR data (GIAO method, *cf.* Computational details, p. 40). The signs of $^{n}J(^{31}P,^{31}P)$ coupling constants were derived from the calculated spectra.

For details regarding NMR spectroscopy under irradiation, see chapter 5.2 (p. 20).

IR spectra of crystalline samples were recorded on a Nicolet 380 FT-IR spectrometer equipped with a Smart Orbit ATR unit at ambient temperature.

Raman spectra of crystalline samples were recorded using a LabRAM HR 800 Horiba Jobin YVON Raman spectrometer equipped with an Olympus BX41 microscope with variable lenses. The samples were excited by a red laser (633 nm, 17 mW, air-cooled HeNe laser). Alternatively, a Buker VERTEX 70 FT-IR spectrometer equipped with a RAM II FT Raman module and an Nd:YAG solid state laser (1604 nm) was used. All measurements were carried out at ambient temperature unless stated otherwise.

Elemental analyses were obtained using an Elementar vario Micro cube CHNS analyser or a LECO TruSpec Micro CHNS analyser.

Melting points (uncorrected) were determined using a Stanford Research Systems EZ Melt at a heating rate of 20 °C/min.

Mass spectra were recorded on a Thermo Electron MAT 95-XP sector field mass spectrometer using crystalline samples.

UV-Vis spectra were acquired on a Perkin-Elmer Lambda 19 UV-Vis spectrometer.

2 Structure elucidation

X-ray Structure Determination: X-ray quality crystals were selected in Fomblin YR-1800 perfluoroether (Alfa Aesar) at ambient temperature. The samples were cooled to 123(2) K during measurement. The data were collected on a Bruker D8 Quest diffractometer using Mo K_{α} radiation (λ = 0.71073 Å). The structures were solved by iterative methods (SHELXT)[4] and refined by full matrix least squares procedures (SHELXL).[5] Semi-empirical absorption corrections were applied (SADABS).[6] All non-hydrogen atoms were refined anisotropically, hydrogen atoms were included in the refinement at calculated positions using a riding model.

Obtaining a good data set of **2**Dmp at low temperatures proved to be somewhat of a challenge since the light of the mounting lamp was sufficient to quantitatively isomerize **2**Dmp to **3**Dmp (which is irreversible at low temperatures), resulting in a microcrystalline material unsuitable for single-crystal X-ray structure analysis. Fortunately, the thermal reverse reaction **3**Dmp → **2**Dmp is fast enough at ambient temperature that single crystals of **2**Dmp stay intact for some time under light. We therefore mounted a crystal of **2**Dmp at room temperature (298 K) and collected a full data set in the dark to verify the purity of the crystal. Afterwards, the crystal was slowly cooled to 123 K under strict exclusion of light, and a low temperature data set was recorded. This procedure allowed us to obtain a phase-pure diffraction pattern of **2**Dmp · 0.5 PhF.

The co-crystallized solvent molecule in **2Dmp_298K** was found to be strongly disordered and was therefore treated as a diffuse contribution to the overall scattering using Platon/Squeeze.

The co-crystallized solvent molecule in **2Dmp_123K** was also found to be disordered, but the disorder was explicitly modelled during the structure refinement by splitting the molecule in two parts. (Due to its location on a crystallographic inversion centre, the overall number of possible orientations of the PhF molecule is doubled, *i.e.* the

disorder is modelled by four different orientations of the PhF molecule.) The position of the major component was fitted using DSR,[7] and the corresponding restraints were kept throughout the refinement. A second layer was added using the same restraints, and both parts were restrained to adopt similar structures using the SAME command. Additionally, the fluorobenzene layers were restrained to adopt a nearly mirror symmetric structure. The ADPs of all atoms within the PhF residue were restrained using the ISOR command.

Table S2: Crystallographic details.

Compound	**2Dmp_298K**	**2Dmp_123K**
Chem. Formula	$C_{57}H_{59}N_3P_2 \cdot 0.5\ (C_6H_5F)$	$C_{57}H_{59}N_3P_2 \cdot 0.5\ (C_6H_5F)$
Formula weight [g/mol]	896.06	896.06
Colour	blue	blue
Crystal system	triclinic	triclinic
Space group	$P\bar{1}$	$P\bar{1}$
a [Å]	10.7104(4)	10.5944(6)
b [Å]	13.0233(5)	12.9038(8)
c [Å]	20.6386(8)	20.3409(13)
α [°]	87.872(2)	85.380(2)
β [°]	83.020(2)	82.564(2)
γ [°]	65.815(2)	66.245(2)
V [Å^3]	2606.38(18)	2522.5(3)
Z	2	2
$\rho_{calc.}$ [g/cm^3]	1.142	1.180
μ [mm^{-1}]	0.125	0.129
T [K]	298(2)	123(2)
Measured reflections	86492	128125
Independent reflections	10222	13422
Reflections with $I > 2\sigma(I)$	7846	9934
R_{int}	0.0691	0.0930
$F(000)$	954	954
$R_1(R[F^2>2\sigma(F^2)])$	0.0456	0.0473
w$R_2(F^2)$	0.1296	0.1117
GooF	1.045	1.024
No. of Parameters	574	700
CCDC #	1869014	1869015

3 Syntheses of starting materials

3.1 Synthesis of DmpNC

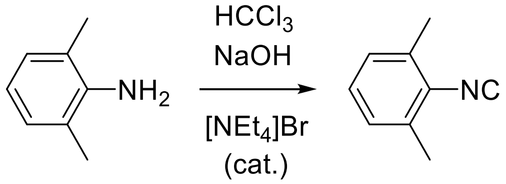

DmpNC is synthesized according to a modified literature procedure.[8] The synthesis is carried out under non-inert conditions.

In a three-necked flask equipped with a reflux condenser and a dropping funnel, $DmpNH_2$ (3.36 g, 29.7 mmol), chloroform (3.55 g, 29.7 mmol), and $[NEt_4]Br$ (90 mg, 0.43 mmol) are dissolved in CH_2Cl_2 (30 mL). Under vigorous stirring, a solution of NaOH (30 g) in water (30 mL) is added slowly over a period of 15 minutes. The mixture is stirred for 18 hours without external heat source, but with the reflux condenser turned on as to prevent evaporation of the organic solvents due to the exothermic reaction. Afterwards, the reaction mixture is diluted with water (200 mL). The aqueous phase is extracted three times with CH_2Cl_2 (50 mL). The solvents of the combined organic phases are evaporated and the solid residue is sublimed thrice (1×10^{-3} mbar, 70 °C). A final sublimation at ambient temperature (1×10^{-3} mbar) yields colourless crystals of the product (2.05 g, 15.6 mmol, 53 %).

Mp. 77 °C. **CHN** % calcd. (found): C 82.41 (82.75); H 6.92 (6.44); N 10.68 (10.90). **^{1}H NMR** (298 K, C_6D_6, 300.13 MHz): δ = 2.06 (s, 6 H, CH_3), 6.59 (d, 2 H, $^3J(^1H,^1H)$ = 7.6 Hz, *p*-CH_3), 6.73 (t, 1 H, $^3J(^1H,^1H)$ = 7.6 Hz, *o*-C*H*). **IR** (ATR, 32 scans, cm^{-1}): $\tilde{\nu}$ = 3475 (vw), 3385 (vw), 2983 (w), 2947 (w), 2920 (w), 2850 (w), 2737 (w), 2119 (s), 2085 (w), 1950 (w), 1880 (w), 1811 (w), 1684 (w), 1622 (w), 1591 (w), 1560 (w), 1471 (s), 1441 (m), 1431 (m), 1387 (m), 1379 (m), 1302 (w), 1282 (w), 1271 (w), 1230 (w), 1171 (m), 1095 (w), 1084 (m),

1038 (m), 991 (w), 976 (w), 924 (w), 800 (m), 775 (vs), 733 (w), 723 (m), 638 (w), 571 (w), 548 (w). **Raman** (633 nm, 20 s, 20 scans, cm^{-1}): $\tilde{\nu}$ = 3083 (1), 3045 (1), 3005 (1), 2944 (2), 2916 (3), 2857 (1), 2731 (1), 1612 (5), 1582 (4), 1482 (2), 1429 (3), 1383 (2), 1376 (3), 1305 (7), 1284 (4), 1244 (1), 1189 (1), 1164 (1), 1093 (2), 1030 (1), 1007 (2), 971 (1), 943 (1), 910 (1), 895 (1), 851 (1), 738 (3), 709 (1), 590 (4), 578 (10), 563 (4), 547 (2), 540 (2), 533 (2), 523 (3), 514 (2), 486 (2), 475 (1), 455 (1), 435 (3), 423 (4), 398 (1), 381 (2), 371 (2), 339 (3), 315 (1), 265 (3), 248 (2), 239 (4), 226 (3), 200 (3), 156 (5).

3.2 Synthesis of TerN(H)PCl_2

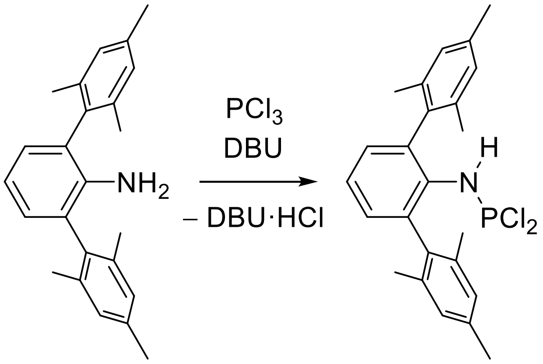

TerN(H)PCl_2 is synthesized according to a slightly modified literature procedure.[2]

PCl_3 (7.07 g, 51.4 mmol) is added to a solution of TerNH_2[2,3] (11.38 g, 34.5 mmol) in Et_2O (150 mL) at −40 °C. Afterwards, DBU (5.25 g, 34.5 mmol) is added dropwise over a period of 10 minutes, whereupon the reaction mixture is allowed to warm to ambient temperature. The mixture is stirred for 20 hours. Afterwards, the solvent is removed *in vacuo*, the remaining white solids are extracted with *n*-hexane (150 mL) and the insoluble residue is separated by filtration. The extraction process is repeated four to five times by back-condensation of the solvent. Finally, the solvent of the filtrate is removed *in vacuo*, yielding the product as a colourless, crystalline solid (13.2 g, 30.7 mmol, 89 %).

Mp. 154 °C. **^{1}H NMR** (298 K, CD_2Cl_2, 250.13 MHz): δ = 2.04 (s, 12 H, *o*-C*H*$_3$), 2.35 (s, 6 H, *p*-CH_3), 5.24 (s, 1 H, N*H*), 7.00 (s, 4 H, Mes *m*-C*H*), 7.06 (m, 2 H, m-C*H*), 7.21 (m, 1 H, *p*-

C*H*). **^{13}C{^{1}H} NMR** (298 K, CD_2Cl_2, 75.5 MHz): δ = 20.8 (d, $J(^{13}C,^{31}P)$ = 1.5 Hz, *o*-CH_3), 21.5 (s, *p*-CH_3), 124.4 (s, CH), 129.3 (s, CH), 130.3 (s, CH), 131.6 (d, $J(^{13}C,^{31}P)$ = 3.8 Hz), 134.9 (d, $J(^{13}C,^{31}P)$ = 3.0 Hz), 137.6 (d, $J(^{13}C,^{31}P)$ = 5.1 Hz), 137.8 (d, $J(^{13}C,^{31}P)$ = 4.1 Hz), 139.0 (s). **^{31}P{^{1}H} NMR** (298 K, CD_2Cl_2, 121.49 MHz): δ = 159.4 (s). **IR** (ATR, 32 scans, cm^{-1}): $\tilde{\nu}$ = 3327 (m), 3030 (w), 2972 (m), 2943 (w), 2914 (m), 2852 (w), 2731 (w), 1610 (m), 1570 (w), 1487 (w), 1431 (s), 1421 (s), 1373 (m), 1356 (s), 1261 (m), 1240 (w), 1217 (s), 1182 (w), 1163 (w), 1101 (w), 1070 (m), 1032 (m), 1009 (m), 970 (w), 947 (m), 918 (s), 852 (vs), 820 (m), 796 (s), 775 (m), 754 (vs), 741 (m), 735 (m), 715 (w), 642 (s), 596 (w), 577 (w), 567 (w), 557 (m), 532 (m). **Raman** (633 nm, 20 s, 20 scans, cm^{-1}): 3331 (1), 3092 (1), 3054 (2), 3016 (1), 2972 (1), 2944 (2), 2915 (5), 2855 (2), 2731 (1), 1612 (4), 1582 (3), 1482 (1), 1440 (2), 1383 (2), 1359 (1), 1305 (10), 1284 (2), 1266 (1), 1217 (5), 1181 (2), 1165 (1), 1071 (5), 1008 (2), 947 (2), 820 (1), 799 (1), 734 (1), 655 (3), 644 (4), 595 (1), 577 (9), 559 (2), 520 (2), 512 (2), 501 (2), 490 (4), 478 (7), 440 (10), 401 (5), 372 (2), 329 (4), 272 (8), 263 (7), 220 (6), 214 (7), 195 (2), 188 (2), 150 (4).

3.3 Synthesis of [ClP(µ-NTer)$_2$]

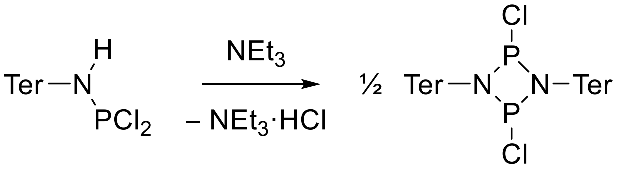

[ClP(µ-NTer)$_2$] is synthesized according to a modified literature procedure.[2]

A stirred solution of TerN(H)PCl_2 (13.2 g, 30.7 mmol) in Et_2O (100 mL) is cooled to −80 °C. NEt_3 (4.86 g, 48 mmol) is added quickly. The mixture is warmed to ambient temperature and stirred overnight, resulting in a slightly yellowish suspension. All volatiles are removed *in vacuo*, and the solid residue is extracted with Et_2O (300 mL) in a Soxhlet apparatus (under argon atmosphere) for 20 hours. The solvent of the extract is removed *in vacuo*, whereupon the product is obtained as a white powder (9.8 g, 12.4 mmol, 81 %).

Note: A mixture of the *cis* and *trans* isomers is obtained (approx. ratio: 4:1). The product may be recrystallized from a minimum of hot benzene.

Mp. 285 °C. **^{1}H NMR** (298 K, C_6D_6, 300.13 MHz): δ = 1.85 (d, 12 H, $^7J(^{31}P,^1H)$ = 1.7 Hz, *trans*, o-C*H*$_3$), 1.91 (s, 12 H, *cis, o*-C*H*$_3$), 2.36 (s, 6 H, *cis*, *p*-C*H*$_3$), 2.46 (s, 6 H, *trans*, *o*-C*H*$_3$), 6.74–7.06 (14 H, *cis/trans* C*H*). **^{13}C{^{1}H} NMR** (298 K, CD_2Cl_2, 75.5 MHz): δ = 21.0 (s, *o*-CH$_3$), 21.3 (t, $J(^{13}C,^{31}P)$ = 2.7 Hz, *o*-CH$_3$), 21.8 (s, *p*-CH$_3$), 21.9 (t, $J(^{13}C,^{31}P)$ = 3.7 Hz, *p*-CH$_3$), 123.7 (s, *C*H), 124.8 (s, *C*H), 128.8 (s, *C*H), 129.1 (s, *C*H), 131.3 (s, *C*H), 131.6 (s, *C*H), 132.4 (t, $J(^{13}C,^{31}P)$ = 1.9 Hz), 134.4 (s), 134.6 (s), 135.2 (t, $J(^{13}C,^{31}P)$ = 2.8 Hz), 135.7 (t, $J(^{13}C,^{31}P)$ = 2.9 Hz), 136.1 (t, $J(^{13}C,^{31}P)$ = 2.8 Hz), 137.9 (s), 138.3 (t, $J(^{13}C,^{31}P)$ = 4.4 Hz), 138.8 (t, $J(^{13}C,^{31}P)$ = 3.5 Hz), 138.9 (t, $J(^{13}C,^{31}P)$ = 3.5 Hz). **^{31}P{^{1}H}-NMR** (298 K C_6D_6, 121.49 MHz): δ = 226.8 (s, *cis*), 363.5 (s, *trans*). **IR** (ATR, 32 scans, cm^{-1}): $\tilde{\nu}$ = 3001 (w), 2962 (w), 2943 (w), 2912 (m), 2852 (w), 2727 (w), 1610 (m), 1581 (w), 1483 (w), 1450 (m), 1410 (s), 1375 (m), 1261 (m), 1223 (vs), 1084 (m), 1030 (m), 1007 (m), 951 (w), 906 (s), 891 (vs), 841 (s), 795 (s), 762 (m), 746 (s), 700 (s), 650 (w), 586 (w), 575 (m), 557 (m), 552 (m), 534 (m). **Raman** (633 nm, 20 s, 20 scans, cm^{-1}): $\tilde{\nu}$ = 3081 (0), 3051 (1), 3009 (1), 3007 (1), 2914 (3), 2854 (1), 2725 (0), 1612 (3), 1581 (3), 1561 (0), 1481 (1), 1435 (2), 1423 (2), 1378 (1), 1374 (1), 1305 (5), 1284 (4), 1275 (2), 1245 (1), 1239 (1), 1225 (0), 1189 (1), 1163 (0), 1157 (0), 1098 (2), 1005 (1), 943 (1), 906 (0), 895 (0), 746 (0), 736 (2), 706 (1), 649 (0), 588 (3), 576 (10), 560 (4), 546 (2), 531 (1), 520 (2), 510 (1), 483 (2), 473 (1), 458 (1), 422 (2), 387 (1), 378 (1), 362 (0), 336 (2), 316 (0), 292 (0), 273 (2), 247 (2), 235 (3), 210 (1), 199 (1), 147 (3).

3.4 Synthesis of [P(μ-NTer)]$_2$ (1)

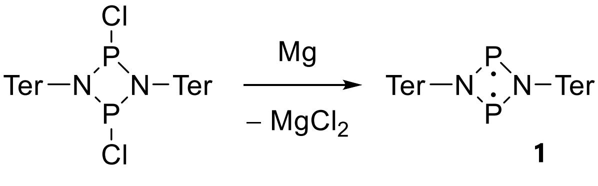

[ClP(μ-NTer)$_2$] is synthesized according to a modified literature procedure.[9,10]

Mg turnings can be activated by stirring for several days using a glass covered magnetic stir bar.

[ClP(μ-NTer)]$_2$ (0.85 g, 1.08 mmol) and Mg turnings (0.40 g, 17 mmol) are combined in a Schlenk flask. *Attention:* It is paramount to ensure that no grease finds its way into the reaction vessel. THF is added (20 mL) and the reaction mixture is stirred overnight, resulting in a brightly orange suspension. The solvents are removed *in vacuo*, and the solid residue is dried at 40 °C for 30 minutes. Benzene (10 mL) is added, and the insoluble material is separated by filtration. The intensively orange filtrate is concentrated to incipient crystallization. After crystallization overnight at ambient temperature, orange crystals of the product are obtained. The supernatant is removed by syringe and re-crystallized, yielding a second crop of product. The isolated crystals are dried *in vacuo*. Yield: 0.65 g (0.91 mmol, 84 %).

Mp. 224 °C (dec.). **^{1}H NMR** (298 K, C_6D_6, 300.13 MHz): δ = 2.04 (s, 24 H, *o*-C*H*$_3$), 2.28 (s, 12 H, *m*-C*H*$_3$), 6.73 (s, 8 H, Mes *m*-C*H*), 6.73 (s, 4 H, Mes *m*-C*H*), 6.89 (m, 2 H, *m*-C*H*), 7.16 (m, 1 H, *p*-C*H*). **^{13}C{^{1}H} NMR** (298 K, C_6D_6, 62.89 MHz): δ = 18.5 (t, $J(^{13}C,^{31}P)$ = 2.3 Hz, *o*-CH$_3$), 19.7 (s, *p*-CH$_3$), 122.6 (s, *C*H), 126.8 (s, *C*H), 128.3 (s, *C*H), 130.6 (t, $J(^{13}C,^{31}P)$ = 1.6 Hz), 133.9 (t, $J(^{13}C,^{31}P)$ = 3.2 Hz), 135.7 (s), 136.35 (t, $J(^{13}C,^{31}P)$ = 3.4 Hz), 137.2 (m, $J(^{13}C\text{-}^{31}P)$ = 2.3 Hz). **^{31}P{^{1}H} NMR** (298 K, C_6D_6, 121.49 MHz): δ = 276.4 (s). **Raman** (633 nm, 20 s, 20 scans, cm^{-1}): $\tilde{\nu}$ = 3083 (1), 3045 (1), 3005 (1), 2944 (2), 2916 (3), 2857 (1), 2731 (1), 1612 (5), 1582 (4), 1482 (2), 1429 (3), 1383 (2), 1376 (3), 1305 (7), 1284 (4), 1244 (1), 1189 (1), 1164 (1), 1093 (2), 1030 (1), 1007 (2), 971 (1), 943 (1), 910 (1), 895 (1), 851 (1), 738 (3), 709 (1), 590 (4), 578 (10), 563 (4), 547 (2), 540 (2), 533 (2), 523 (3), 514 (2), 486 (2), 475 (1), 455 (1), 435 (3), 423 (4), 398 (1), 381 (2), 371 (2), 339 (3), 315 (1), 265 (3), 248 (2), 239 (4), 226 (3), 200 (3), 156 (5).

4 Syntheses of compounds

4.1 Synthesis of the biradical {[PN(Ter)]$_2$(µ-CNDmp)} (2Dmp)

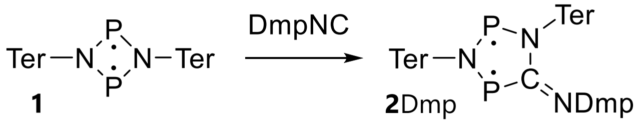

2Dmp is synthesized according to a slightly modified literature procedure.[8,11]

A solution of DmpNC (170 mg, 1.29 mmol) in benzene (5 mL) is added to a solution of [P(µ-NTer)]$_2$ (927 mg, 1.29 mmol) in benzene (20 mL). An immediate colour change from orange to green to dark blue is observed. After five minutes, the solution is concentrated to incipient crystallization and left undisturbed overnight, yielding dark blue (almost black) crystals of the product. The supernatant is removed by syringe and re-crystallized. The crystals are dried *in vacuo*. Yield: 965 mg (1.14 mmol, 88 %).

Single crystals suitable for X-ray structure determination can be obtained by re-crystallization from PhF at ambient temperature.

Mp. 207 °C. **CHN** % calcd. (found): C 80.73 (79.99); H 7.01 (6.29); N 4.95 (4.81). **^{1}H NMR** (298 K, THF-d$_8$, 250.13 MHz): δ =1.28 (s, 6 H, C*H*$_3$), 1.55 (s, 6 H, C*H*$_3$), 1.81 (s, 12 H, C*H*$_3$), 1.96 (s, 12 H, C*H*$_3$), 2.34 (s, 12 H, C*H*$_3$), 2.32 (s, 12 H, C*H*$_3$), 6.47-7.44 (m, 17 H, C*H*). **^{31}P{^{1}H} NMR** (298 K, THF-d$_8$, 101.25 MHz) δ = 220.5 (d, 1 P, J(^{31}P,^{31}P) = 134 Hz, N–*P*–C), 258.3 (d, 1 P, J(^{31}P,^{31}P) = 134 Hz, N–*P*–N). **Raman** (1064 nm, 592 mW, 1500 scans): $\tilde{\nu}$ = 3056 (2), 3017 (1), 2915 (4), 2852 (1), 2731 (1), 1613 (6), 1593 (2), 1578 (4), 1538 (10), 1486 (2), 1414 (2), 1376 (4), 1305 (6), 1285 (2), 1249 (3), 1226 (6), 1194 (1), 1189 (1), 1164 (1), 1159 (1), 1103 (1), 1093 (1), 1083 (1), 1057 (1), 1025 (1), 1017 (1), 1007 (1), 990 (2), 981 (0), 950 (2), 945 (2), 887 (0), 837 (1), 751 (0), 743 (0), 589 (0), 576 (1), 559 (1), 531 (1), 524 (1), 511 (1), 508 (1), 503 (1), 491 (1), 477 (1), 426 (1), 421 (1),

412 (1), 391 (1), 339 (1), 318 (1), 283 (1), 274 (1), 266 (1), 248 (1), 237 (1), 232 (1), 228 (1), 198 (1), 187 (1), 178 (1), 153 (2), 149 (2).

4.2 Generation of the housane {[PN(Ter)]$_2$(μ-CNDmp)} (3Dmp)

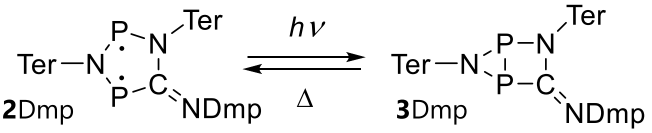

The housane **3**Dmp can be generated by irradiation of a solution of **2**Dmp with red (or white) light (λ_{max} = 643 nm). A solution of the housane is stable for several weeks at −80 °C.

^{31}P{^{1}H} NMR (298 K, THF-d$_8$, 101.25 MHz) δ = −129.2 (d, 1 P, J(^{31}P,^{31}P) = −65 Hz, N–*P*–C), −63.4 (d, 1 P, J(^{31}P,^{31}P) = −65 Hz, N–*P*–N).

When crystals of **2**Dmp are irradiated at −80 °C, microcrystalline **3**Dmp can be obtained, as evidenced by decolouration and the Raman spectrum of the crystalline material.

Raman (785 nm, 20 s, 8 scans, cm^{-1}): ṽ = 3055 (0), 2921 (0), 1699 (0), 1640 (3), 1613 (2), 1592 (4), 1581 (3), 1489 (1), 1466 (1), 1432 (2), 1407 (2), 1377 (3), 1307 (5), 1288 (2), 1266 (3), 1249 (2), 1220 (2), 1187 (2), 1178 (3), 1165 (2), 1087 (3), 1073 (3), 1007 (2), 990 (5), 946 (2), 882 (2), 849 (1), 809 (1), 792 (1), 768 (1), 763 (1), 742 (2), 701 (3), 686 (2), 649 (4), 635 (6), 604 (1), 588 (4), 577 (10), 572 (4), 563 (4), 559 (3), 554 (4), 537 (1), 530 (2), 525 (4), 511 (2), 499 (5), 467 (5), 445 (5), 422 (2), 411 (3), 394 (2), 378 (2), 353 (2), 337 (3), 328 (2), 314 (2), 308 (2), 274 (3), 260 (4), 242 (8), 218 (3), 149 (6).

4.3 Synthesis of {[PN(Ter)]$_2$(µ-CN^{t}Bu)$_2$} (**4**^{t}Bu$_2$)

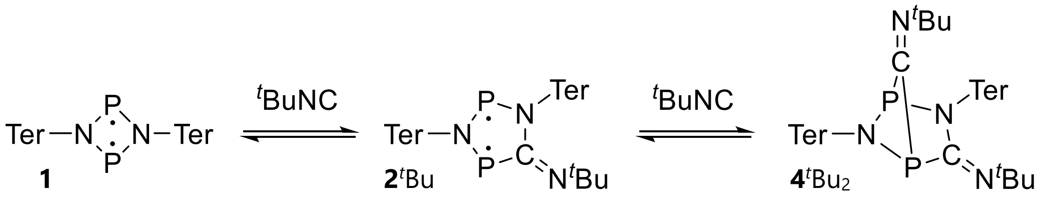

4^{t}Bu$_2$ is synthesized according to a slightly modified literature procedure.[8]

^{t}BuNC (94.7 µL, 0.84 mmol) is added to a solution of [P(µ-NTer)]$_2$ (**1**, 300 mg, 0.42 mmol) in benzene (10 mL) at ambient temperature, resulting in an immediate colour change from orange to dark green. The solution is stirred for two hours, which is accompanied by a gradual colour change to pale yellow. Subsequently, all volatiles are removed *in vacuo*, and the foamy residue is thoroughly dried *in vacuo* for further 30 minutes. The product is then crystallized from a minimal amount of benzene at ambient temperature. Yield: 298 mg (0.34 mmol, 81 %).

Single crystals suitable for X-ray structure determination can be obtained from saturated benzene solution. The identity of **4**^{t}Bu$_2$ was verified by single-crystal X-ray structure determination (*cf.* CCSD #1421779).

Mp. 164 °C (dec.). **CHN** calcd. (found): C 79.06 (79.33), H 7.55 (7.34), N 6.36 (5.98). **^{31}P{^{1}H} NMR** (298 K, THF-d_8, 101.25 MHz): Due to an equilibrium in solution, three different species are observed. **4**^{t}Bu$_2$ δ = 166.5 (d, 1 P, $^2J(^{31}P,^{31}P)$ = 33 Hz, N–*P*–C), 210.6 (d, 1 P, $^2J(^{31}P,^{31}P)$ = 33 Hz, N–*P*–N); **2**^{t}Bu δ = 194.9 (d, 1 P, $^2J(^{31}P,^{31}P)$ = 142 Hz, N–*P*–C), 259.9 (d, 1 P, $^2J(^{31}P,^{31}P)$ = 142 Hz, N–*P*–N); **1** δ = 275.9 (s, 2 P). **Raman** (633 nm, 20 s, 10 scans, cm^{-1}): $\tilde{\nu}$ = 3054 (2), 3044 (3), 3006 (3), 2977 (3), 2966 (3), 2915 (10), 2855 (3), 2729 (1), 1608 (3), 1577 (6), 1482 (2), 1451 (2), 1439 (3), 1401 (4), 1373 (3), 1301 (10), 1280 (3), 1269 (3), 1245 (5), 1223 (5), 1212 (6), 1184 (3), 1160 (2), 1094 (4), 1084 (3), 1066 (3), 1025 (2), 1001 (3), 943 (2), 916 (1), 880 (1), 826 (2), 801 (1), 786 (1), 739 (3), 689 (1), 651 (1), 602 (3), 589 (4), 574 (10), 553 (6), 540 (4), 530 (3), 521 (4), 507 (2), 468 (2), 452 (2), 408 (2), 394 (1), 333 (1), 301 (2), 243 (6), 219 (4).

4.4 Generation of the housane {[PN(Ter)]2(μ-CN*t*Bu)} (3*t*Bu)

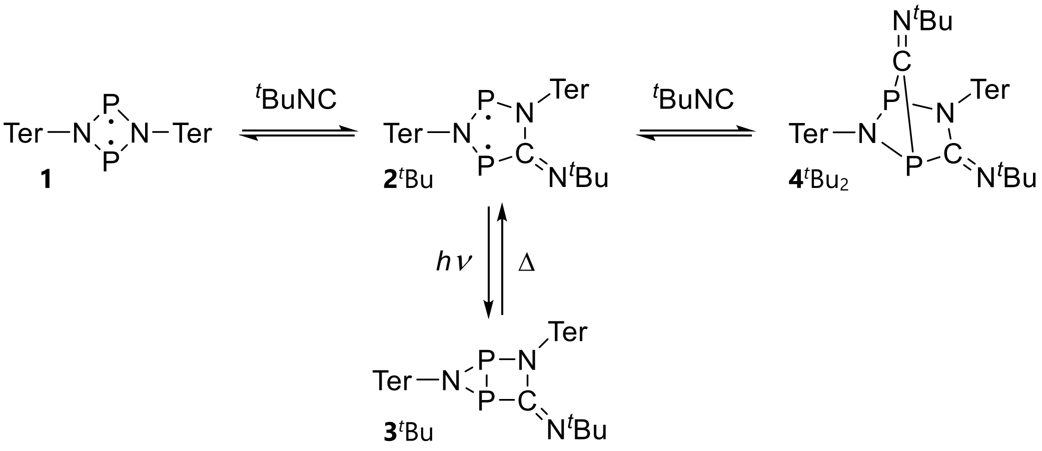

The housane **3**^{t}Bu can be generated by irradiating a solution of **4**^{t}Bu$_2$ in benzene or THF. A solution of the housane is stable for several weeks at −80 °C.

^{31}P{^{1}H} NMR (298 K, THF-d_8, 101.25 MHz): δ = −106.6 (d, 1 P, $^2J(^{31}P,^{31}P)$ = 63 Hz, N–*P*–C), −60.9 (d, 1 P, $^2J(^{31}P,^{31}P)$ = 63 Hz, N–*P*–N).

4.5 Synthesis of {[PN(Ter)]2(μ-CN*t*Bu)2} (4Dmp*t*Bu)

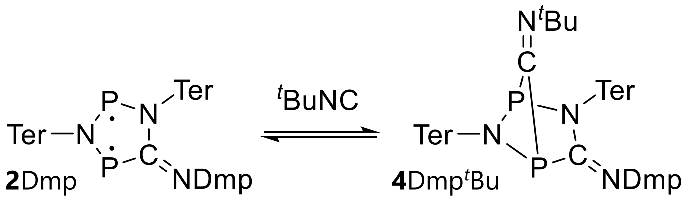

A blue solution of {[PN(Ter)]2(μ-CNDmp)} (**2**Dmp, 116 mg, 0.14 mmol) in benzene (5 mL) is treated with 1 equiv. of ^{t}BuNC (15.5 μL, 11.4 mg, 0.14 mmol). As evidenced by ^{31}P NMR spectroscopy, only a small amount of the desired product is formed (ca. 30 %).

When ^{t}BuNC is added in large excess (0.25 mL, 184 mg, 2.21 mmol), the ratio between product and starting material increases to 9:1.

Isolation of the pure product failed, partly due to scrambling of the different isonitrile moieties after a few days. Moreover, the solution could not be concentrated *in vacuo*, since this led to removal of ^{t}BuNC and therefore re-formation of the starting material.

^{31}P{^{1}H} NMR (298 K, C_6D_6, 121.49 MHz): **4**Dmp^{t}Bu δ = 188.6 (d, 1 P, $^{2}J(^{31}P,^{31}P)$ = 31 Hz, N–*P*–C), 223.1 (d, 1 P, $^{2}J(^{31}P,^{31}P)$ = 31 Hz, N-*P*-N); **2**Dmp δ = 221.5 (d, 1 P, $^{2}J(^{31}P,^{31}P)$ = 135 Hz, N–*P*–C), 258.1 (d, 1 P, $^{2}J(^{31}P,^{31}P)$ = 135 Hz, N–*P*–N).

5 Additional spectroscopic details

5.1 UV-Vis spectra, extinction coefficient

Figure S1: UV-Vis absorption spectrum of **2**Dmp in benzene (λ_{max} = 643 nm).

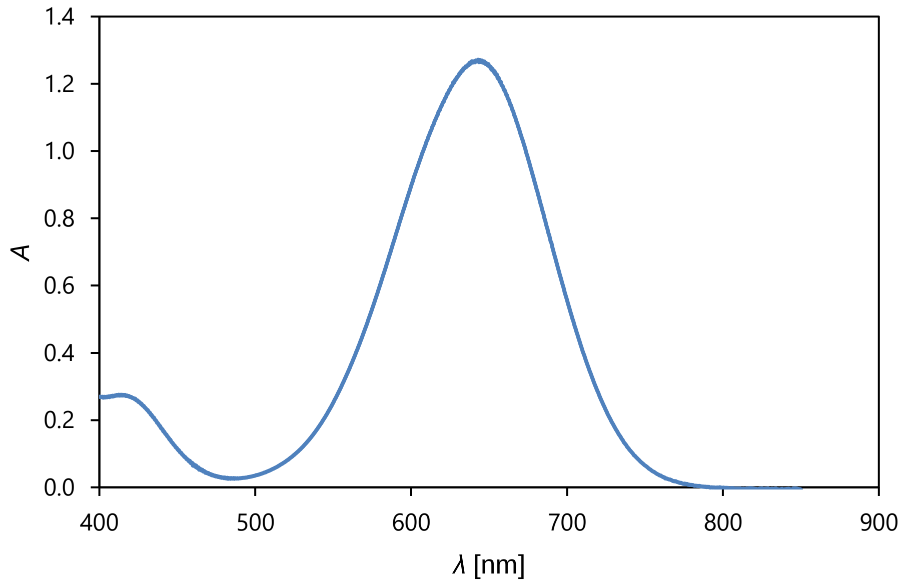

The biradicaloid **2**Dmp exhibits a broad absorption maximum centered at 643 nm with a full width at half maximum (FWHM) of approx. 120 nm (Figure S1). Hence, it absorbs light in the red region of the visible spectrum and therefore appears blue. The molar extinction coefficient ε was determined from differently concentrated solutions by linear regression (Figure S2) following the Beer-Lambert law,

$$A = \varepsilon \cdot c \cdot L$$

where A is the absorbance, c the concentration of the analyte, and L the optical path length.

Figure S2: Absorbance at λ_{max} *vs.* concentration according to the Beer-Lambert law.

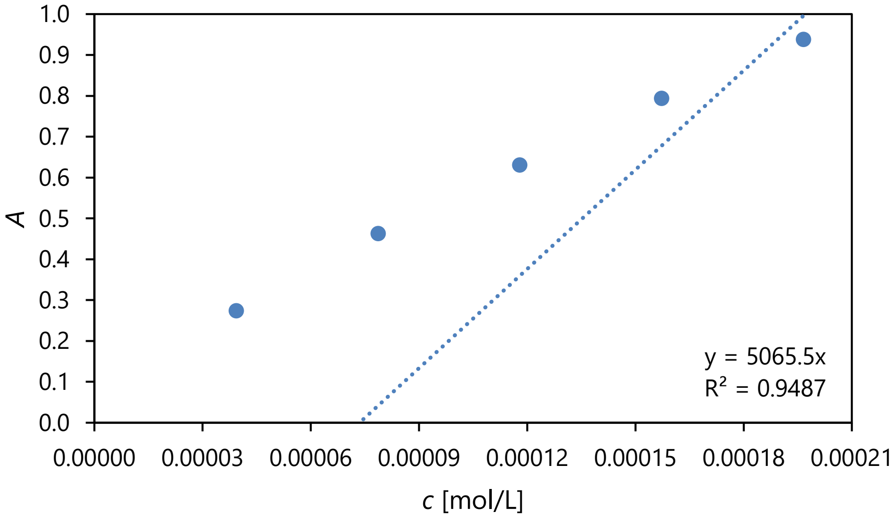

Due to the small concentrations and high sensitivity of the biradical towards moisture, the margin of error is rather large. The extinction coefficient at 643 nm (absorption maximum) was determined as ε_{643} = 5.1(2)×10^5 L mol^{-1}m^{-1} and at 633 nm (laser wavelength used for irradiation experiments, *vide infra*) as ε_{633} = 5.0(2)×10^5 L mol^{-1}m^{-1}.

The ^{t}Bu derivative **2**^{t}Bu could be observed in solution in equilibrium with **4**^{t}Bu$_2$. The absorption maximum was determined at 663 nm (Figure S3), *i.e.* the excitation energy is somewhat smaller than in case of **2**Dmp.

Figure S3: UV-Vis absorption spectrum of a solution of **4**^{t}Bu$_2$ in benzene (λ_{max} = 663 nm).

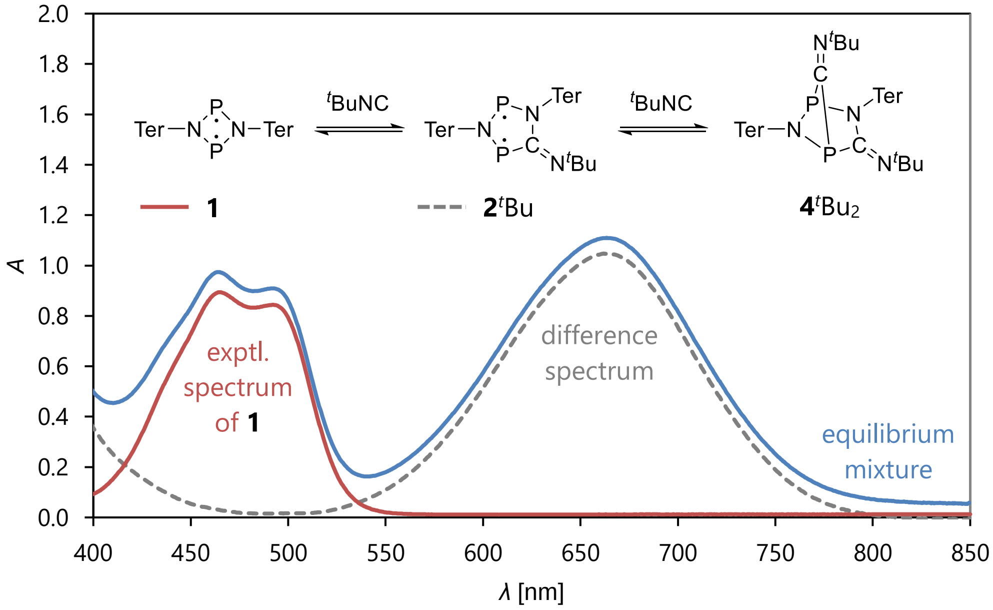

5.2 NMR spectroscopy under irradiation

To facilitate NMR spectroscopy under irradiation, we adopted a setup previously published by the Gschwind group,[12] who used a fibre-coupled light emitting diode (LED) to direct light into the NMR spectrometer.

In the original publication, the optical fibre was coupled with an LED by placing the polished end of the fibre directly on top of the diode. Unlike commercial solutions, this simple setup does not depend on sophisticated lens systems to focus light into the optical fibre; nonetheless, it offers efficient coupling since the area of the diode is almost completely covered by the cross section of the optical fibre.

Instead of an LED, we decided to use a laser diode (Oclaro HL63193MG, 638 nm, 700 mW), primarily due to the high optical output power at a low price (≈ 80 €) and secondly due to the narrow emission spectrum. Since the opening of the laser diode was only somewhat larger than the cross section of the optical fibre (10 m multimode fibre, 0.39 NA, high OH, 1000 µm core diameter, ThorLabs FT1000UMT), we decided to use the same optical coupling between the diode and the optical fibre as described above (Figure S4).

Figure S4: Schematic view of the laser diode and the optical fibre. Efficient coupling was achieved by mounting the fibre directly onto the glass window of the diode.

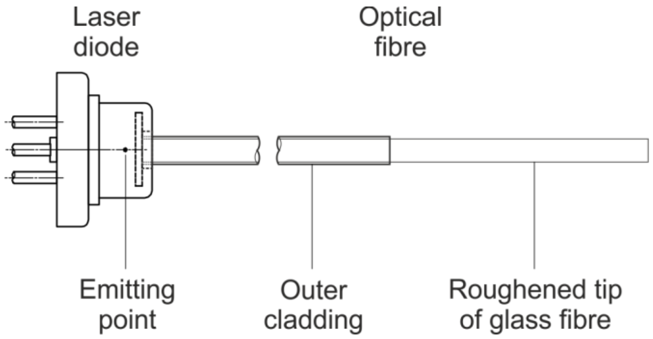

The diode was mounted in a metal casing to dissipate heat. The casing accepts a screw-on brass cylinder that can be used to fix the optical fibre directly on top of the diode

and that also serves as an additional heat sink. Moreover, the cylinder may be inserted into an aluminium block (70×70×30 mm^3) to further dissipate heat (Figure S5).

Figure S5: Photo (left) and schematic depiction (right) of the coupling between laser diode and optical fibre (A: laser diode, B: metal casing, C: bass cylinder with a PTFE insert to hold the optical fibre, D: aluminium block, E: optical fibre).

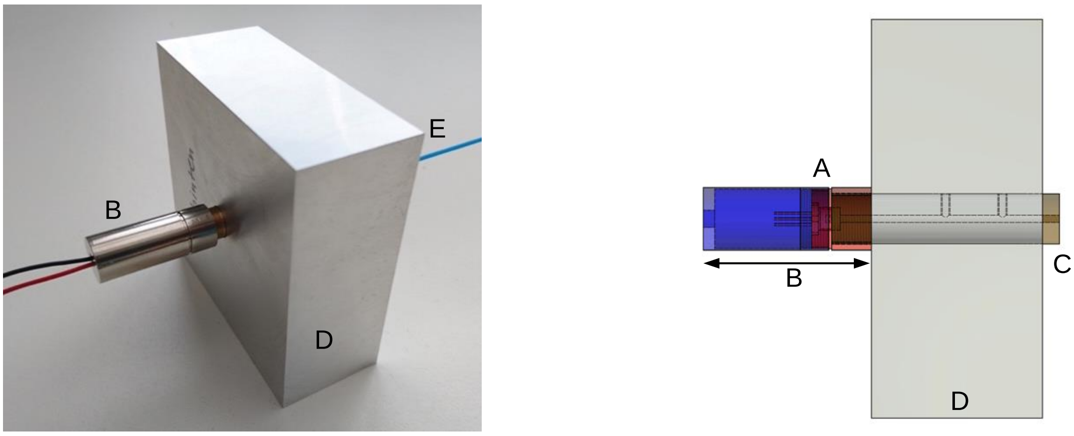

Figure S6: Optical output power measured at the diode (P_{diode}) and at the end of the optical fibre (P_{fibre}).

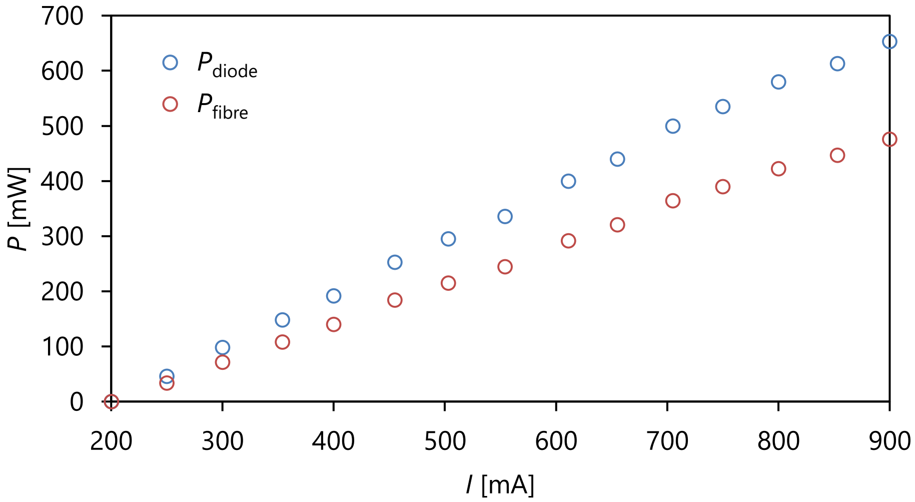

The optical coupling factor was determined by the ratio of the optical output power at the end of the optical fibre and directly at the diode (neglecting absorption/scattering within the optical fibre). With a value of 0.73, the coupling is quite efficient. The output power of the optical fibre was measured to be about 480 mW at a maximum current of 900 mA (Figure S6). The diode can run continuously at 500 mA (215 mW optical

output) for more than one hour. At currents above 700 mA, heat quickly becomes a problem and the diode can only be switched on for short intervals of time.

As described in the original publication of the Gschwind group,[12] the outer cladding of the optical fibre was removed at the tip and the glass surface was roughened to allow uniform irradiation of the sample within the spectrometer. The fibre was inserted into an NMR tube with a coaxial insert (Figure S7). To ensure inert conditions, all samples were prepared in a glovebox and the tubes were sealed with custom-made PTFE caps as well as 2–3 layers of PTFE tape.

Figure S7: Schematic view of an NMR tube with coaxial insert and optical fibre.

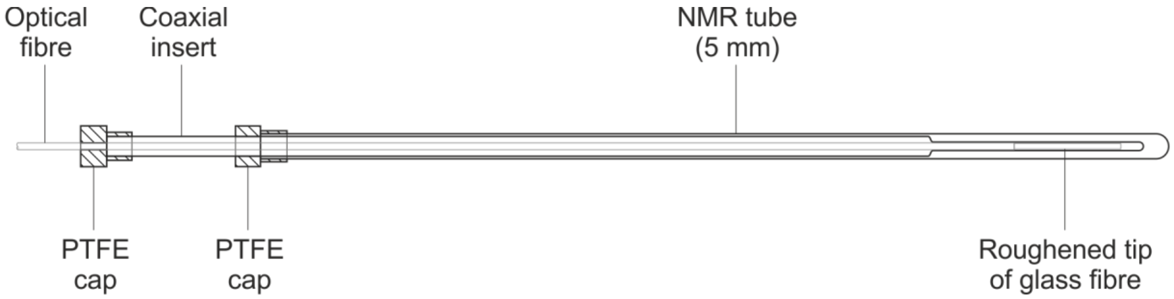

Figure S8: The sample inlet is equipped with a cap which ensures that the optical fibre is centred above the NMR tube.

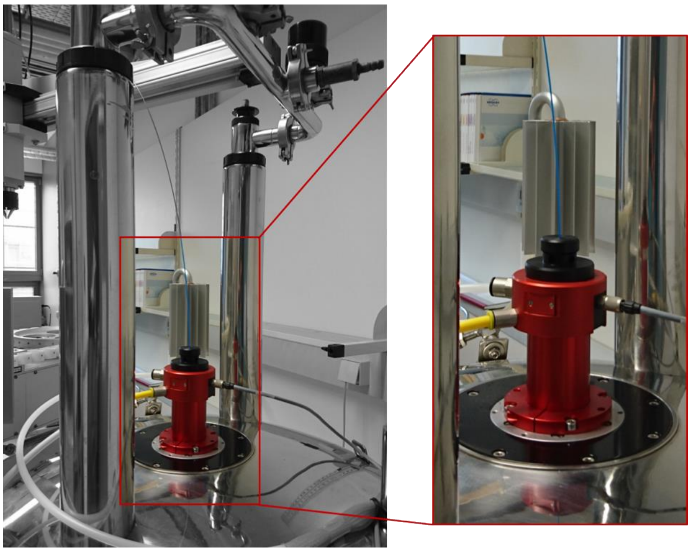

To ensure that the optical fibre cannot exert lateral forces and thereby tilt the NMR tube in the spectrometer, a cap with a central bore was placed on top of the

spectrometer's sample inlet. The optical fibre is passed through the bore to keep it centred above the NMR tube (Figure S8).

5.3 Photoisomerization

To study the photoisomerization process, crystals of **2**Dmp (21 mg) were dissolved in THF-d_8 (0.45 mL). PPh_3 (11 mg, 0.06 mmol) was added as internal standard. The sample solution was filled in an NMR tube with a coaxial insert (*vide supra*). At first, NMR spectra were recorded in the dark, showing that only the biradical species **2**Dmp was present (Figure S9, top).

Figure S9: ^{31}P NMR spectra of **2**Dmp recorded in the dark (top) and under irradiation (bottom).

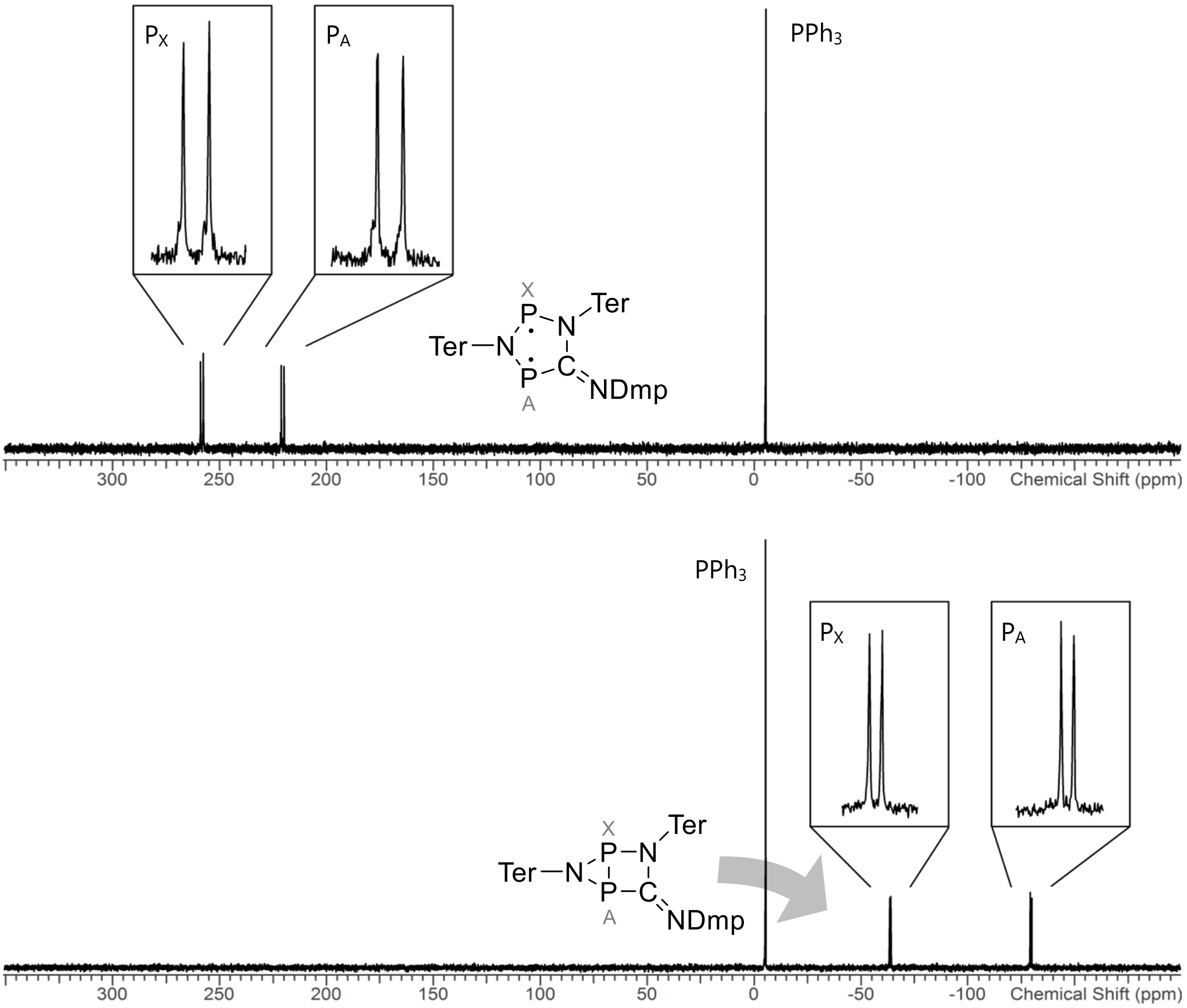

The sample was then irradiated in the spectrometer (638 nm, 500 mA) for approx. two minutes. Subsequently, ^{1}H and ^{31}P NMR spectra were measured under irradiation, evidencing that the biradical had quantitatively isomerized to the housane-type species **3**Dmp (Figure S9, bottom).

In a next set of experiments, the behaviour of the ^{t}Bu derivative **2**^{t}Bu under irradiation was investigated. Since it is formed in equilibrium from the adduct **4**^{t}Bu$_2$, crystals of the latter (14.9 mg, 0.02 mmol) were dissolved in THF-d_8 (0.45 mL). PPh_3 (11.5 mg, 0.06 mmol) was added as internal standard. The blue solution was then irradiated, resulting in quantitative formation of the housane species **3**^{t}Bu (Figure S10), *i.e.* both **2**^{t}Bu and **4**^{t}Bu$_2$ were fully consumed.

Figure S10: ^{31}P NMR spectra of **4**^{t}Bu$_2$ recorded in the dark (top) and under irradiation (bottom).

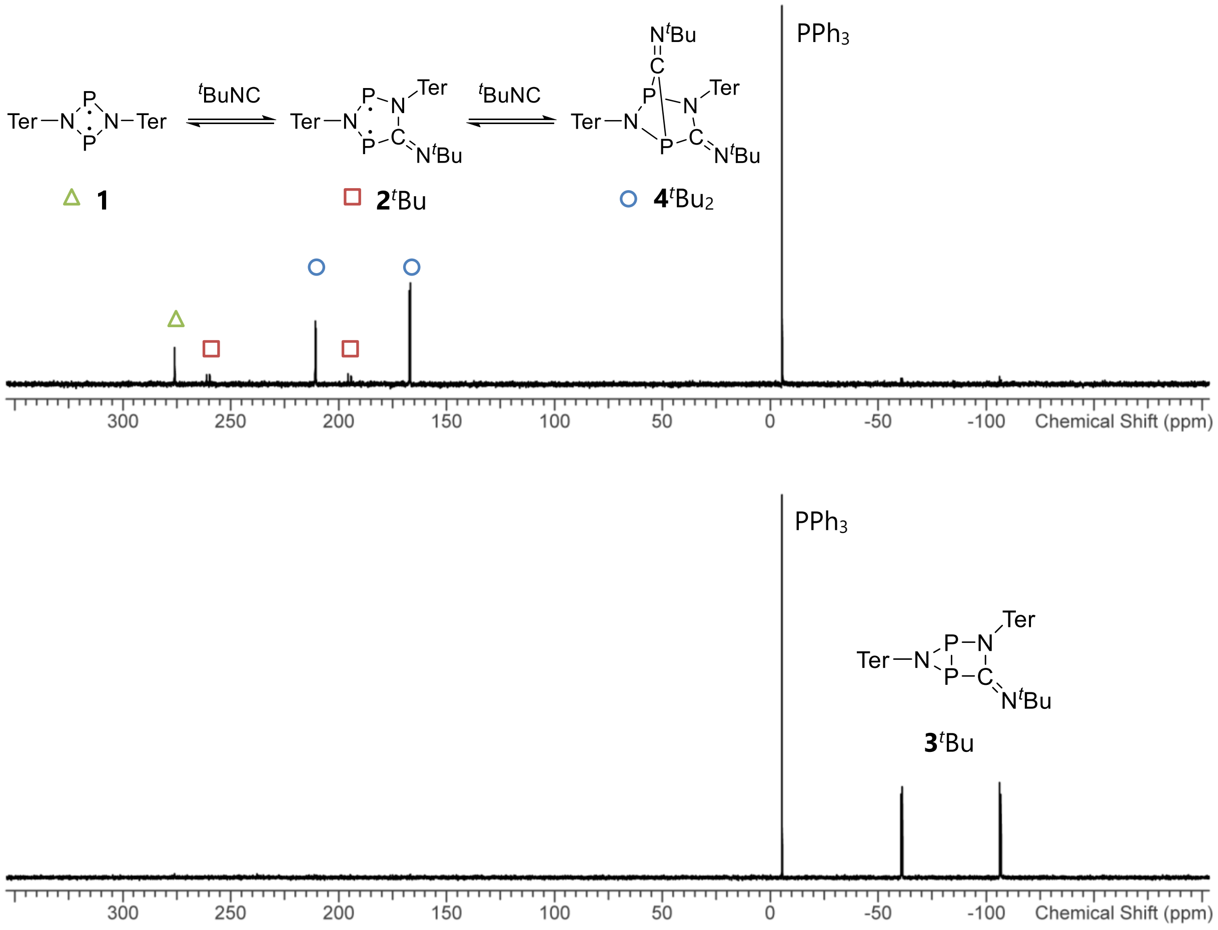

5.4 Thermal equilibration

To investigate the thermal reverse reaction **3**Dmp → **2**Dmp, a solution of the biradical **2**Dmp was irradiated in the NMR spectrometer as described above. After full conversion of the biradical to the housane **3**Dmp, the laser diode was turned off and the thermal equilibration (Scheme S1) was traced by *in-situ* ^{31}P NMR spectroscopy. Initially, a spectrum was collected every three minutes (75 scans).

Scheme S1: Isomerization of **2**Dmp under irradiation and thermal reverse reaction of **3**Dmp.

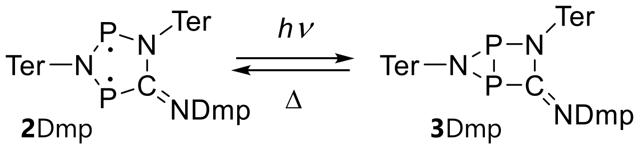

At 298 K; the reverse reaction was complete within 30 minutes, with a half-life of about 7 minutes (Figure S11).

Figure S11: Time-resolved ^{31}P NMR spectra recorded after irradiation.

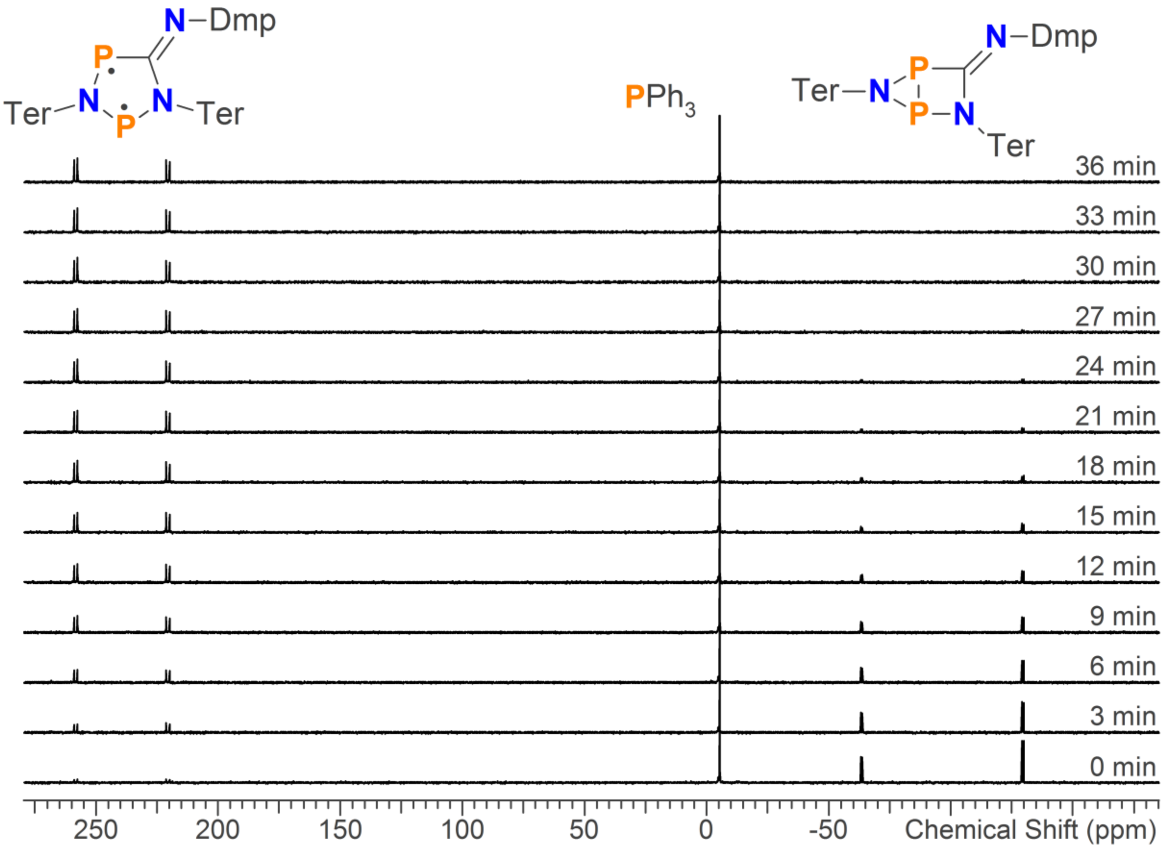

The time-dependent concentration data could be fitted as a first-order reaction according to the integrated time laws,

$$[\mathrm{A}] = [\mathrm{A}]_0 \cdot \exp(-kt)$$

$$[\mathrm{B}] = [\mathrm{A}]_0 \cdot [1 - \exp(-kt)] = [\mathrm{A}]_0 - [\mathrm{A}]$$

where $[\mathrm{A}]$ and $[\mathrm{B}]$ are the concentrations of **3**Dmp and **2**Dmp, respectively. $[\mathrm{A}]_0$ is the initial concentration at $t = 0$, and t is fitted as $t + t_{\mathrm{offset}}$ to account for the time interval t_{offset} between switching off the light source and completed acquisition of the first spectrum.

The experiment was repeated at different temperatures (Figure S12 ff) to determine the temperature dependence of the rate constant and thus the enthalpy and entropy of activation, $\Delta H^{\ddagger}$ and $\Delta S^{\ddagger}$, according to the Eyring theory:

$$k = \kappa \frac{k_{\mathrm{B}} T}{h} \exp\left(-\frac{\Delta G^{\ddagger}}{RT}\right)$$

$$\Rightarrow \quad \ln\left(\frac{k}{T}\right) = -\frac{\Delta H^{\ddagger}}{R} \cdot \frac{1}{T} + \frac{\Delta S^{\ddagger}}{R} + \ln\left(\frac{k_B}{h}\right)$$

where κ is assumed to be 1 in the second equality.

Figure S12: Thermal reverse reaction at −5 °C (A = **3**Dmp, B = **2**Dmp; k = 3.3(2)×10^{-5} s^{-1}).

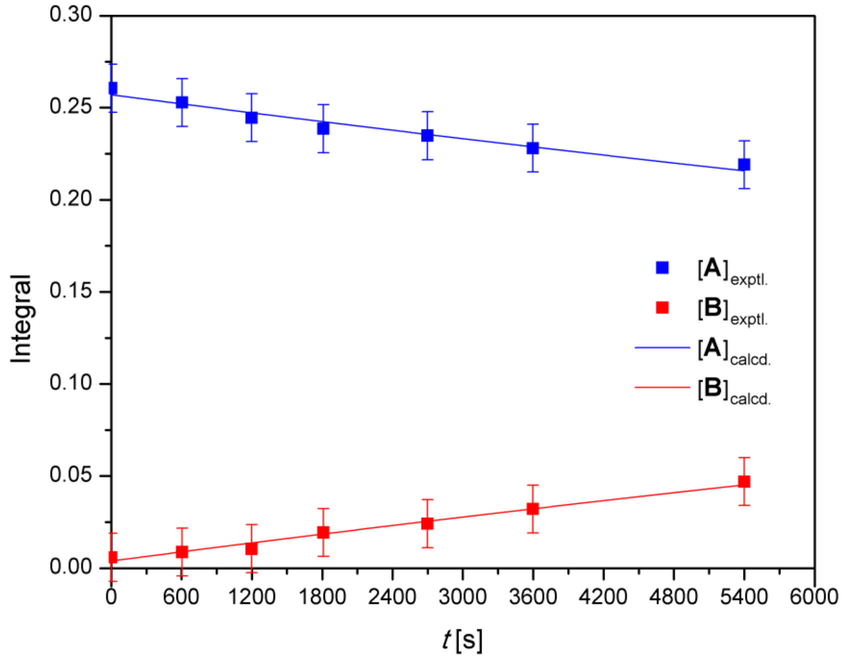

Figure S13: Thermal reverse reaction at +5 °C (A = **3**Dmp, B = **2**Dmp; k = 1.10(2)×10^{-4} s^{-1}).

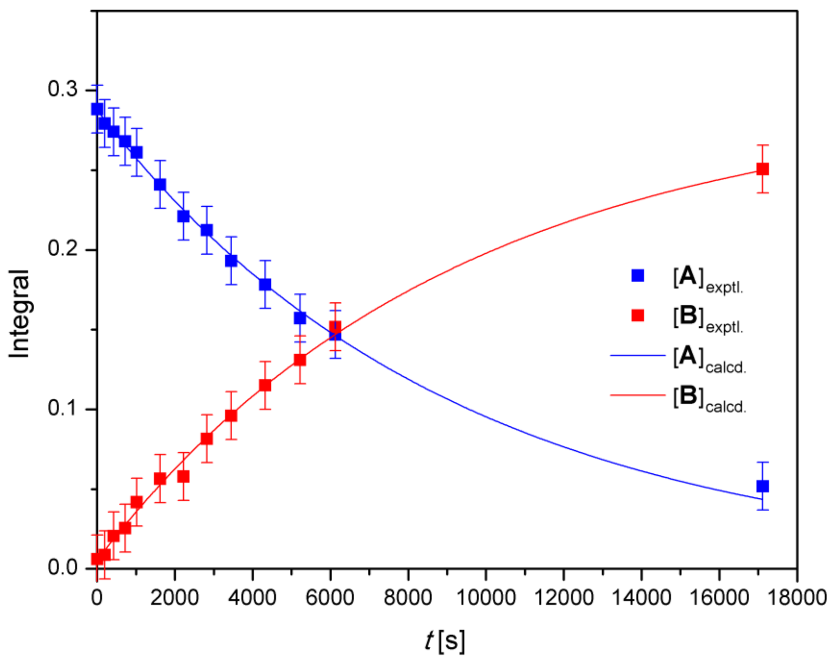

Figure S14: Thermal reverse reaction at +15 °C (A = **3**Dmp, B = **2**Dmp; k = 4.13(5)×10^{-4} s^{-1}).

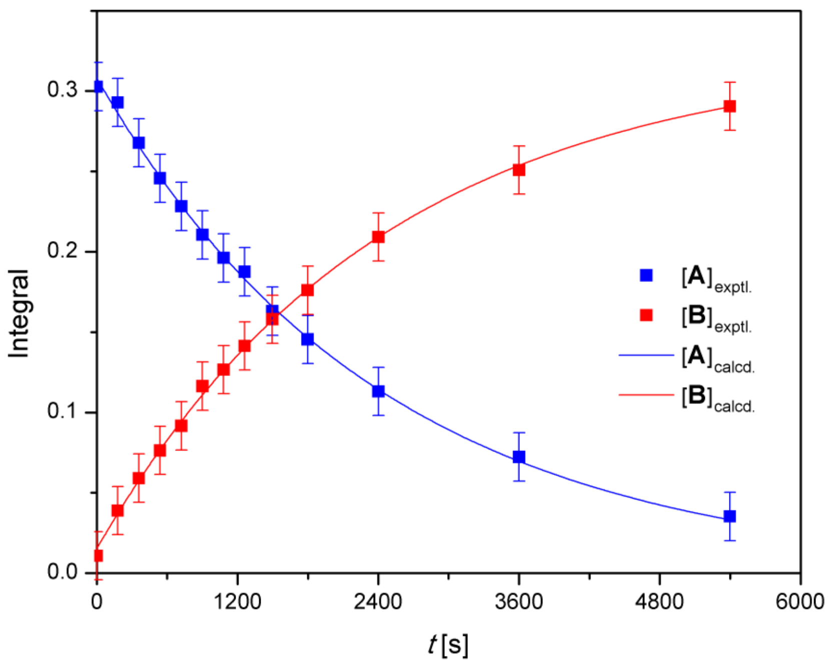

Figure S15: Thermal reverse reaction at +25 °C (A = **3**Dmp, B = **2**Dmp; k = 1.73(3)×10^{-3} s^{-1}).

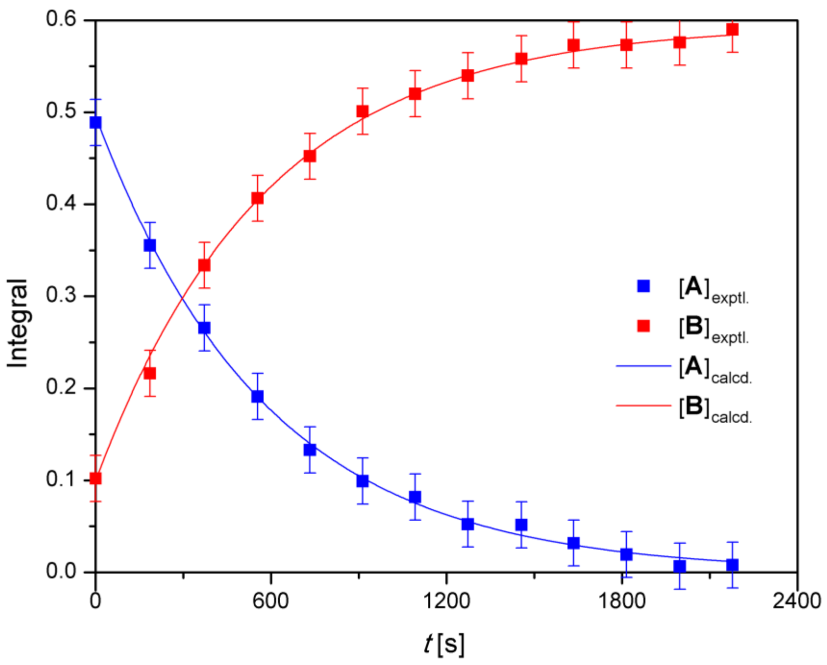

Figure S16: Thermal reverse reaction at +35 °C (A = **3**Dmp, B = **2**Dmp; k = 7.6(5)×10^{-3} s^{-1}).

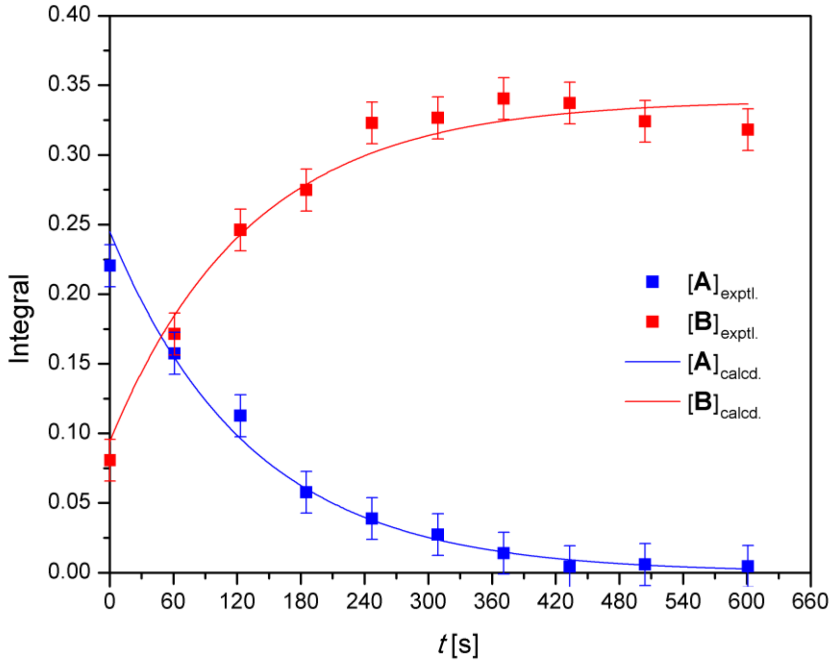

Figure S17: Eyring plot ($\Delta H^{\ddagger}$ = 91.5±0.3 kJ/mol; $\Delta S^{\ddagger}$ = 10.7±10.5 J·mol^{-1}·K^{-1}).

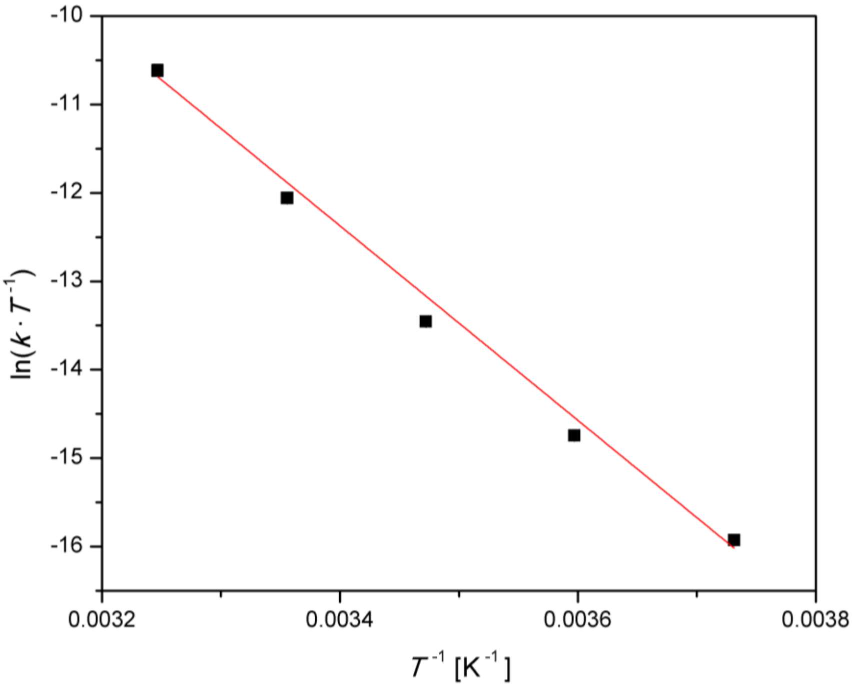

Hence, the Gibbs free energy of activation $\Delta G^{\ddagger}$ at 25 °C amounts to 88(4) kJ/mol.

A similar experiment was carried out in the UV-Vis spectrometer at ambient temperature: A freshly prepared, blue solution of **2**Dmp in benzene was irradiated with white light (Xe lamp), resulting in discolouration of the solution. The sample cuvette was placed into the spectrometer and the absorbance at 643 nm was measured once every second. The plot of absorbance *vs.* time nicely corresponds to the rate law of product formation of a first order reaction, $[\mathrm{B}] = [\mathrm{A}]_0 \cdot [1 - \exp(-kt)]$ (Figure S18).

Figure S18: Time-dependent absorbance (643 nm) at ambient temperature. Non-linear regression gave k = 2.395(4)×10^{-3} s^{-1}.

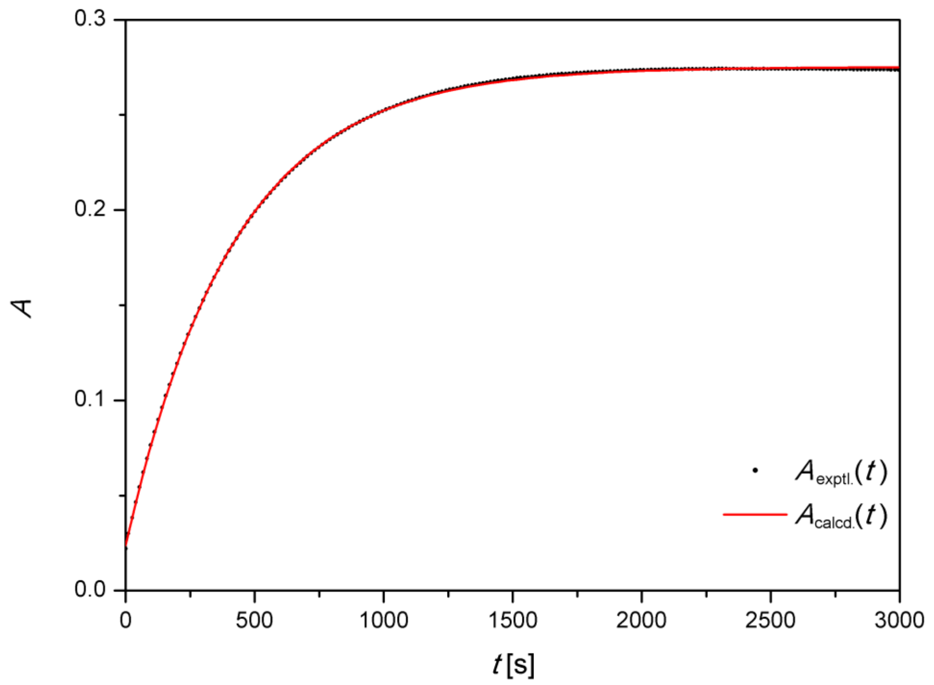

As described before, dissolving crystals of **4**^{t}Bu$_2$ yielded an equilibrium mixture of **1**, **2**^{t}Bu and **4**^{t}Bu$_2$. Irradiation could shift the complete equilibrium towards the photoactivation product **3**^{t}Bu, whose thermal reverse reaction (Scheme S2) was monitored by ^{31}P NMR spectroscopy. As in case of **3**Dmp, the reverse reaction was found to be a first-order reaction. The species **1**, **2**^{t}Bu and **4**^{t}Bu$_2$ were recovered in their equilibrium ratios, *i.e.* the thermal reverse reaction of **3**^{t}Bu is the rate determining step (Figure S19).

Scheme S2: **2**^{t}Bu is continuously removed from the equilibrium by irradiation, leading to quantitative formation of the housane **3**^{t}Bu. When the light source is switched off, the equilibrium mixture between **1**, **2**^{t}Bu and **4**^{t}Bu$_2$ is fully recovered.

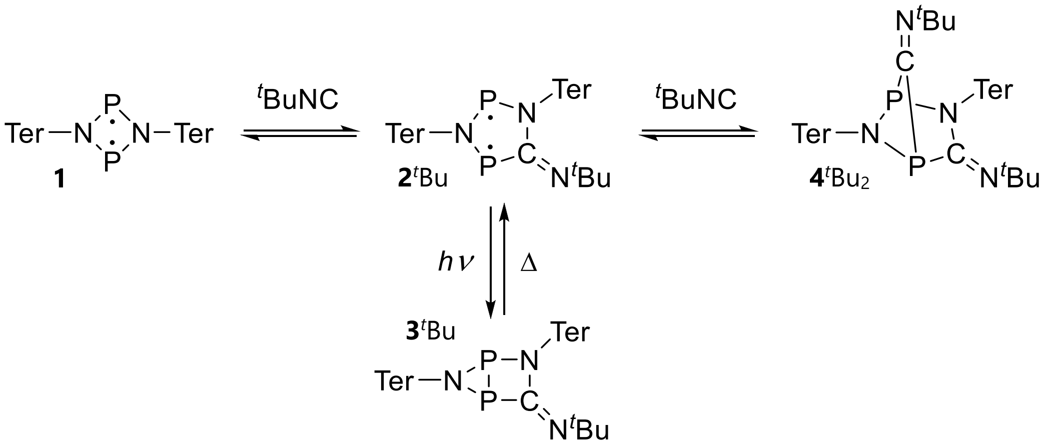

Figure S19: Thermal reverse reaction at +25 °C (k = 2.03(4)×10^{-4} s^{-1}, $t_{1/2}$ ≈ 57 min).

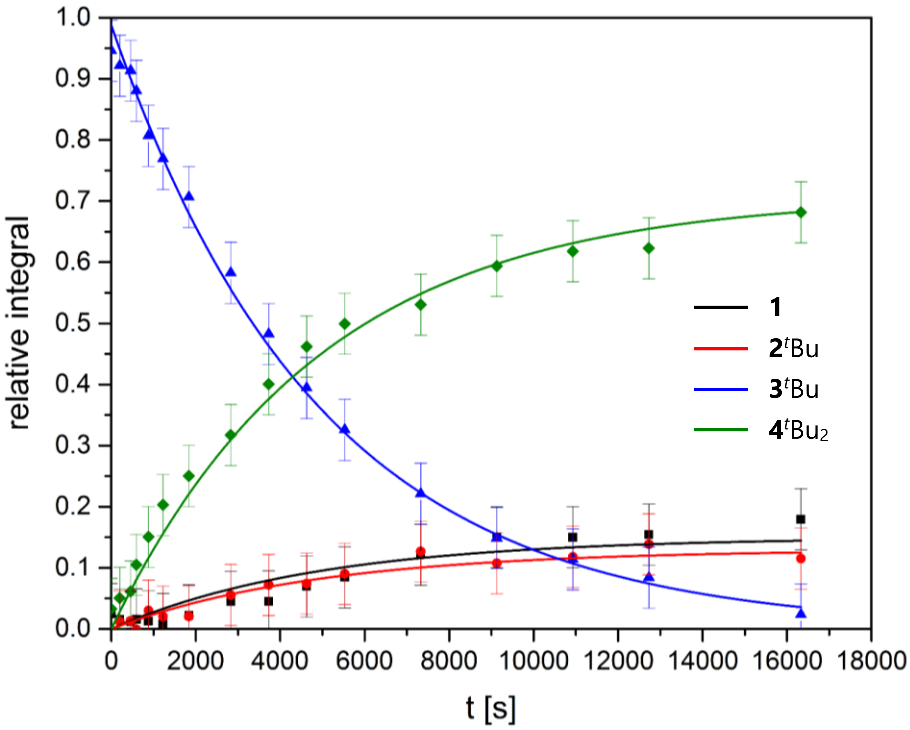

When a solution of **2**Dmp in THF-d_8 was treated with ^{t}BuNC, an equilibrium mixture of **2**Dmp and **4**Dmp^{t}Bu was obtained (*cf.* chapter 4.5). Intriguingly, it was not possible to generate **3**Dmp quantitatively. Even after prolonged irradiation (ca. 20 minutes), some amounts of **2**Dmp and **4**Dmp^{t}Bu remained in solution (Figure S20). Furthermore, as

soon as the light was switched off, the housane species **3**Dmp completely disappeared, and the equilibrium distribution of **2**Dmp and **4**Dmp^{t}Bu was recovered. We attributed this behaviour to a catalytic influence of ^{t}BuNC on the thermal reverse reaction of the housane **3**Dmp (Scheme S3).

Scheme S3: The thermal equilibration of **3**Dmp is most likely catalysed by ^{t}BuNC.

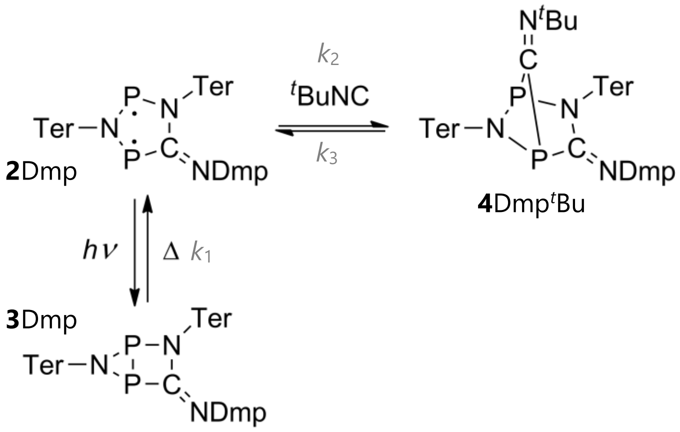

Figure S20: ^{31}P NMR spectra of **2**Dmp + ^{t}BuNC recorded in the dark (top) and under irradiation (bottom).

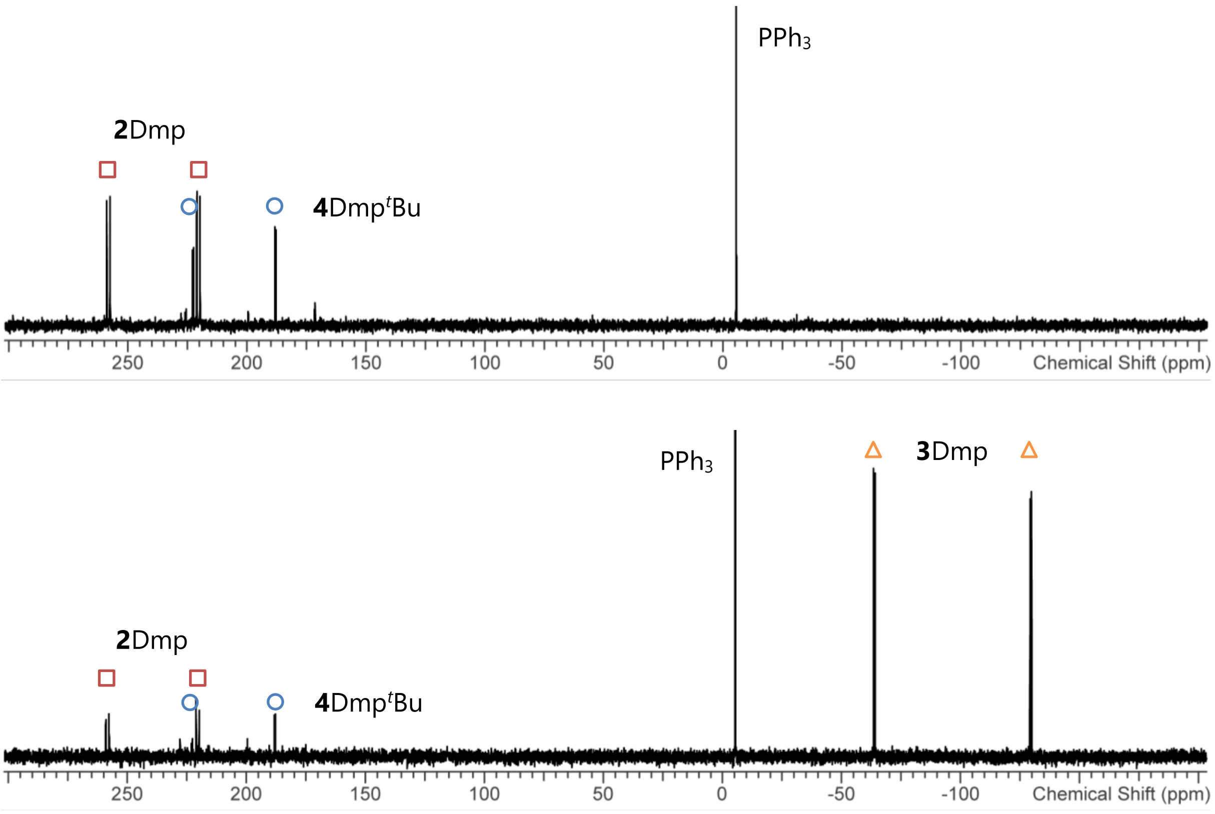

To further validate this theory, a solution of **2**Dmp (30 mg, 0.036 mmol) and PPh_3 (internal standard, 10.8 mg, 0.041 mmol) in THF-d_8 (0.45 mL) was treated with 1 equiv. of ^{t}BuNC (4.1 mg, 0.036 mmol), which led to partial formation of **4**Dmp^{t}Bu (ca. 30 %). The sample was cooled to −20 °C and irradiated. At this lower temperature, full conversion of **2**Dmp to **3**Dmp was observed. The light source was switched off and the reverse reaction was monitored by ^{31}P NMR spectroscopy as outlined before (Figure S21).

Figure S21: Thermal reverse reaction at −20 °C. The data was fitted according to the model described above (*cf.* Scheme S3).

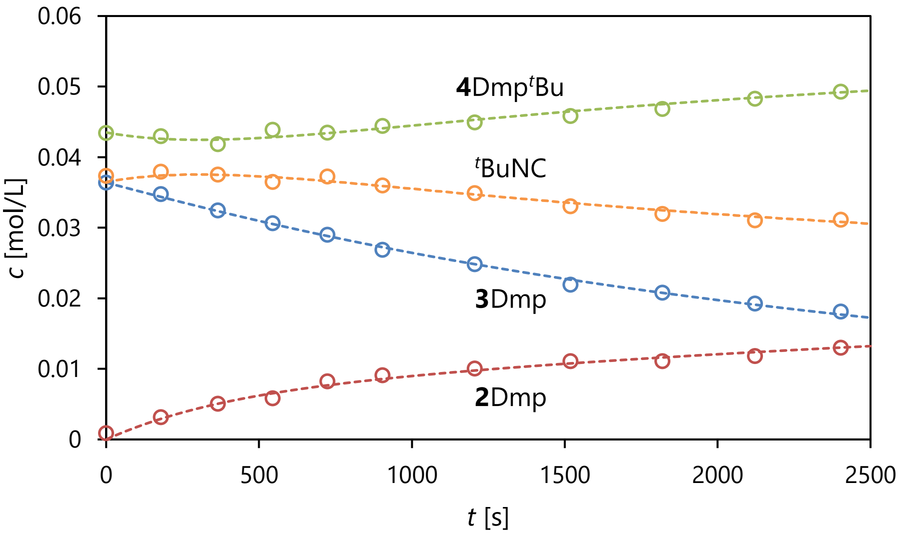

The reverse reaction proceeded much faster than in the absence of ^{t}BuNC. Our results indicate that $k_1 \approx 8.4\times10^{-3}$ L mol^{-1}s^{-1}, *i.e.* it is a bimolecular reaction between ^{t}BuNC and **3**Dmp (*cf.* Scheme S3). Since no further intermediate was observed, it is likely that the addition of ^{t}BuNC to the housane **3**Dmp is the rate determining step and that the subsequent elimination of ^{t}BuNC leading to the biradical is much faster. However, it should be noted further research is needed to fully understand the mechanism.

5.5 Determination of the quantum yield of the photoisomerization

To determine the quantum yield of the photoisomerization process, a fresh solution of the biradical **2**Dmp in benzene is filled in a cuvette with an optical path length L of 1 cm. The sample is then irradiated with a HeNe-laser operating at 633 nm and the transmitted laser intensity is monitored by a photodiode behind the sample. During irradiation, the concentration of the biradical decreases due to photoisomerization and the transmission at the laser wavelength increases, since the photoproduct does not absorb light in the visible spectral range. The increase in transmission is a measure for the number of converted molecules and the quantum yield is determined as the ratio of this number to the number of absorbed photons. Since the excitation intensity varies within the sample due to its absorption, the dependence of the process on the x coordinate along the sample must be considered as follows.

The concentration $c_\mathrm{b}(x,t)$ of the biradical **2**Dmp obeys rate equation (1):

$$\frac{d}{dt}c_\mathrm{b}(x,t) = -\phi \cdot \left(-\frac{d}{dx}\frac{I(x,t)}{N_\mathrm{A} \cdot h\nu}\right) + k_\mathrm{r} \cdot c_\mathrm{h}(x,t) \qquad (1)$$

ϕ is the quantum yield, $I(x,t)$ the excitation intensity within the sample, N_A the Avogadro constant, h the Planck constant, ν the optical frequency of the excitation photons, k_r the rate of the thermal reverse reaction, and c_h the concentration of the photo product **3**Dmp. To simplify the situation, the laser intensity is chosen high enough to guarantee that the photo conversion is much faster than the reverse reaction. In this case, the last term in eq. (1) can be neglected. For the measurement, the quantities integrated over the optical path in the sample are the relevant ones. Introducing the integrated column density $\rho_\mathrm{b}(t) \coloneqq \int_0^L c_\mathrm{b}(x,t)\,dx$ and neglecting the reverse reaction, eq. (1) can be rewritten as:

$$\frac{d}{dt}\rho_\mathrm{b}(t) = -\frac{\phi}{N_\mathrm{A} \cdot h\nu} \cdot \left(I(0,t) - I(L,t)\right) = -\frac{\phi}{N_\mathrm{A} \cdot h\nu} \cdot I_0 \cdot \left(1 - 10^{-A(t)}\right)$$
$$= \frac{\phi}{N_\mathrm{A} \cdot h\nu} \cdot I_0 \cdot \left(10^{-\varepsilon \cdot \rho_\mathrm{b}(t)} - 1\right) \qquad (2)$$

$I_0 = I(0)$ is the incident and temporal constant laser intensity, ε the molar extinction coefficient at 633 nm (5.0(2)×10^5 L mol^{-1}m^{-1}), and $A(t) = \int_0^L \varepsilon \cdot c_\mathrm{b}(x,t)\,dx = \varepsilon \cdot \rho_\mathrm{b}(t)$ the absorbance. Eq. (2) is integrated as follows:

$$\frac{d\rho_\mathrm{b}(t)}{10^{-\varepsilon \cdot \rho_\mathrm{b}(t)} - 1} = \frac{\phi}{N_\mathrm{A} \cdot h\nu} I_0 dt$$

$$\left[\frac{1}{ln(10)\varepsilon} ln\left(\frac{1}{1 - 10^{\varepsilon \cdot \rho_\mathrm{b}(t)}}\right)\right]_0^t = ln\left(\frac{1 - 10^{\varepsilon \cdot \rho_\mathrm{b}(0)}}{1 - 10^{\varepsilon \cdot \rho_\mathrm{b}(t)}}\right)\frac{1}{ln(10)\varepsilon} = \frac{\phi}{N_\mathrm{A} \cdot h\nu} I_0 t$$

$$\frac{1 - 10^{\varepsilon \cdot \rho_\mathrm{b}(0)}}{1 - 10^{\varepsilon \cdot \rho_\mathrm{b}(t)}} = 10^{\frac{\phi \varepsilon I_0}{N_\mathrm{A} \cdot h\nu} t}$$

The signal $sig(t)$ of the photodiode is proportional to the transmitted laser intensity $I_0 \cdot 10^{-\varepsilon \cdot \rho_\mathrm{b}(t)}$ and with the proportionality constant a defined by $10^{-\varepsilon \cdot \rho_\mathrm{b}(t)} = a \cdot sig(t)$ one obtains eq. (3):

$$1 - 10^{\varepsilon \cdot \rho_\mathrm{b}(t)} = \left(1 - 10^{\varepsilon \cdot \rho_\mathrm{b}(0)}\right) 10^{-\frac{\phi \varepsilon I_0}{N_\mathrm{A} \cdot h\nu} t}$$

$$1 - \frac{1}{a \cdot sig(t)} = \left(1 - \frac{1}{a \cdot sig(0)}\right) 10^{-\frac{\phi \varepsilon I_0}{N_\mathrm{A} \cdot h\nu} t}$$

$$\frac{1}{sig(t)} = a + \left(\frac{1}{sig(0)} - a\right) 10^{-\frac{\phi \varepsilon I_0}{N_\mathrm{A} \cdot h\nu} t} \qquad (3)$$

Fitting eq. (3) to the measured time-dependent signal of the photodiode gives the exponential constant $b = \phi \varepsilon I_0 / (N_\mathrm{A} h\nu)$ and thus the quantum yield.

Figure S22: Experimental setup for the determination of the quantum yield.

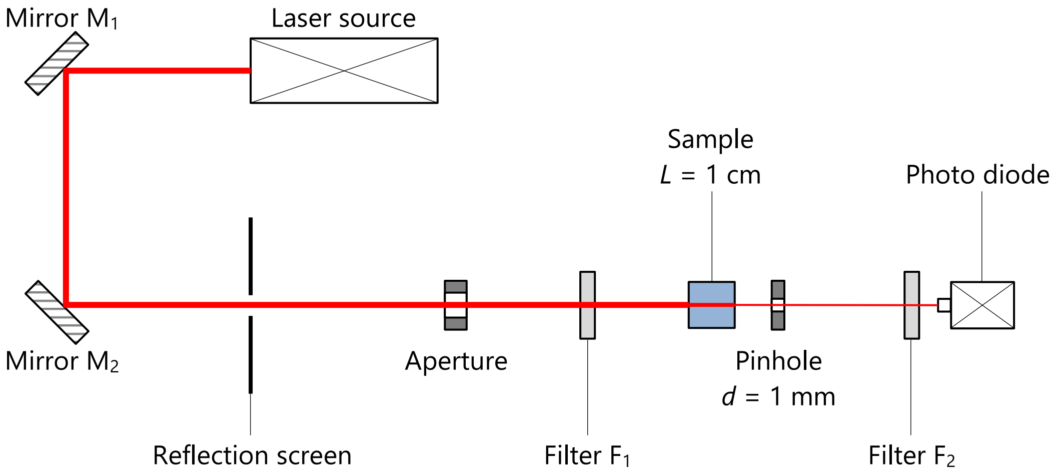

The experimental setup is shown in Figure S22. The incident laser intensity I_0 is derived by measuring the optical power output of the laser behind the pinhole (d = 1 mm), initially without a sample in the beam path (P_{laser}), and again after placing a cuvette filled with pure benzene in the sample holder (P_{blank}). The decrease of the measured power is mostly attributed to partial reflection of the laser light upon passing through the front and back windows of the cuvette. The incident intensity I_0 at the location of the sample solution is therefore approximated as

$$I_0 = \left(P_{\text{laser}} - \frac{P_{\text{laser}} - P_{\text{blank}}}{2}\right) \cdot \frac{4}{\pi d^2} \tag{4}$$

Using a pinhole with a diameter smaller than the profile of the laser beam ensures that diffusion perpendicular to the beam bath can be neglected. The photoconversion is only monitored in the centre of the actual beam; thus, the concentration gradient at the edges of the examined cross section is small.

By changing the filter at position F_1, the rate of conversion can be controlled. It is essential to minimize the duration of each measurement, so the reverse reaction as well as diffusion and convection within the sample can be neglected (*vide supra*). The time resolution Δt is chosen accordingly. The signal of the diode is sampled at 2 kHz and the measured output voltage of the photodiode U is averaged over Δt. The filter F_2 is chosen such that the photo diode is not saturated. To reduce statistical uncertainty, each measurement is repeated several times and the data are merged for evaluation. An overview of the collected data sets is given in Table S3; Figure S23 and Figure S24 show the plots of $1/U = 1/sig(t)$ versus t and the fitted curves according to eq. (3) for each data set.

Table S3: Collected data sets (overview).

#	A (633 nm)	Filter F_1	P_{Laser} [μW]	P_{blank} [μW]	I_0 [W/m^2]	Filter F_2	t [s]	Δt **[s]**	Data points	Φ [%]
1	0.18	–	262	232	315	D0.6	15	0.25	458	24.2±1.2
2	0.33	–	265	234	318	D0.6	15	0.25	407	24.9±1.2

Figure S23: Plot of $1/U = 1/sig(t)$ versus t for data set #1. Fit parameters: $a = 5.500(2)\ \mathrm{V}^{-1}$, $b = 0.200(2)\ \mathrm{s}^{-1}$, $1/sig(0)\ = 7.75(1)\ \mathrm{V}^{-1}$.

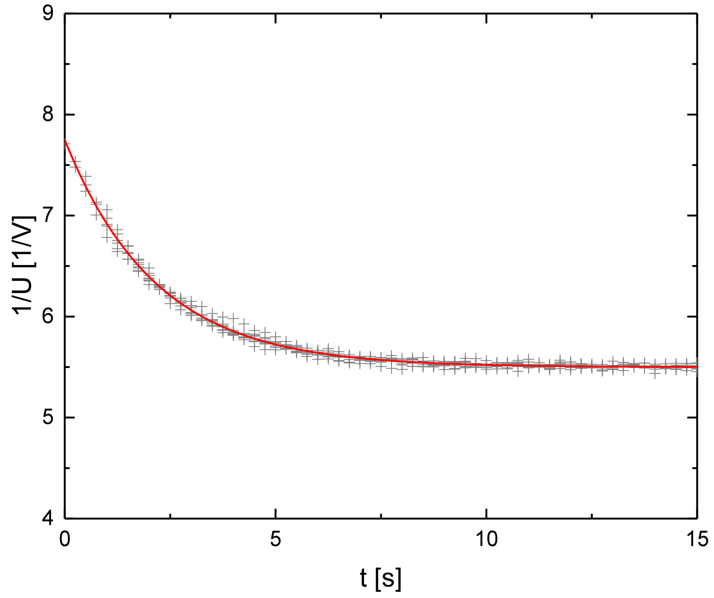

Figure S24: Plot of $1/U = 1/sig(t)$ versus t for data set #2. Fit parameters: $a = 6.041(4)\ \mathrm{V}^{-1}$, $b = 0.208(1)\ \mathrm{s}^{-1}$, $1/sig(0)\ = 10.86(2)\ \mathrm{V}^{-1}$.

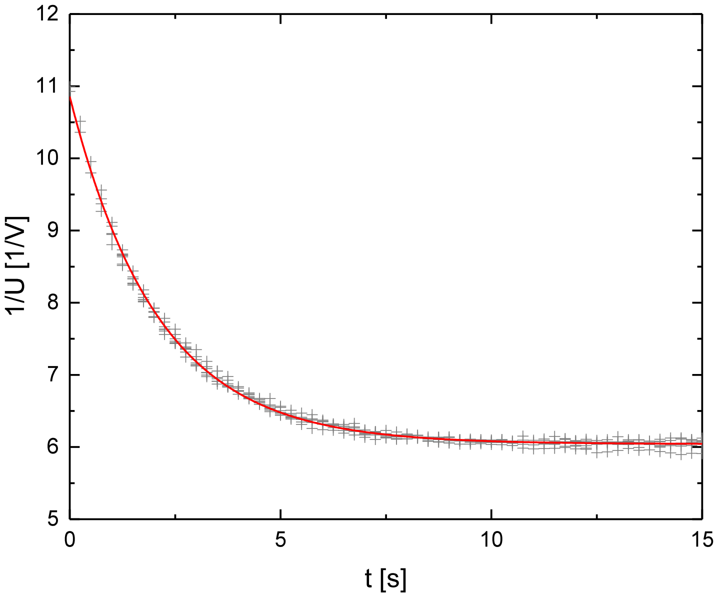

5.6 Raman microscopy

Recording Raman spectra of **2**Dmp and **3**Dmp was anything but trivial. Due to light-induced isomerization, samples of **2**Dmp could not be excited using visible light (otherwise, a spectrum of a mixture of both isomers would be obtained). Therefore, we decided to use a 1064 nm laser, which should not be able to trigger isomerization. To acquire a spectrum of **3**Dmp, a single crystal of **2**Dmp was irradiated with a red laser at −80 °C, resulting in isomerization of the biradical to the housane. Raman spectra were then recorded using a red (633 nm) or an infrared (785 nm) laser. Still, it cannot be ruled out that small amounts of **2**Dmp were detected in the spectrum of **3**Dmp (Figure S25).

Figure S25: Details of the Raman spectra of **2**Dmp (bottom) and **3**Dmp (top). In the upper spectrum, the C=N stretch of the former isonitrile group is discernible at 1640 cm^{-1}. In the biradical, the CN double bond is partly delocalized into the π system, so it resonates at lower wave numbers and is superimposed by vibrations of the Ter and Dmp substituents.

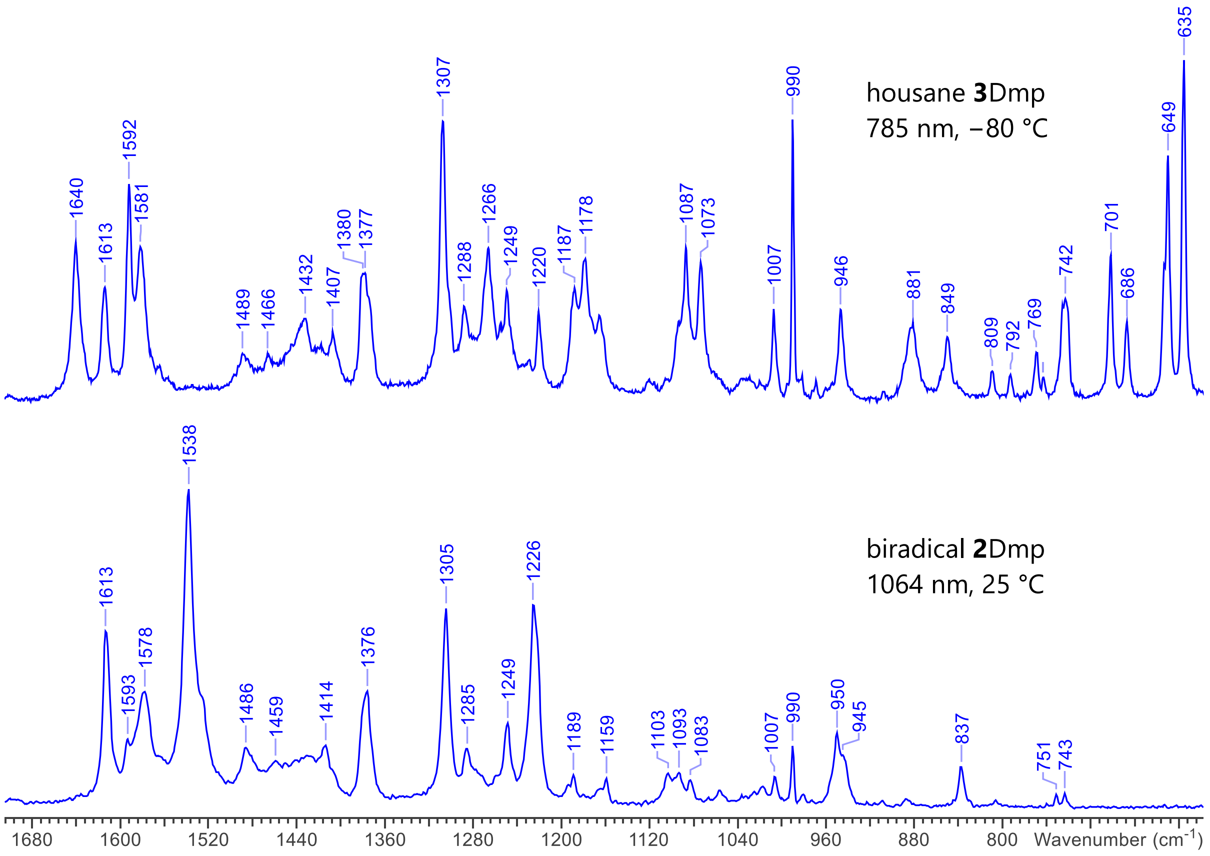

5.7 Fluorescence spectroscopy

We attempted to record fluorescence spectra of a solution of **2**Dmp in benzene. However, no fluorescence was observed, in accordance with our calculations that predict a conical intersection between the S_0 and S_1 surfaces (*cf.* chapter 6.2) and thus radiationless deactivation of the first excited singlet state.

6 Computational details

Electronic structure computations were carried out using Gaussian 09[13] or ORCA 4.0.1.[14,15] NBO analyses were performed using NBO 6.0.[16–19]

Since biradicals require multi-determinantal wave functions, they cannot be properly described by typical single-reference methods such as HF, DFT or coupled cluster theory. A qualitatively correct wave function can be obtained by multi-configurational SCF (MCSCF) methods which describe non-dynamic correlation, *e.g.* Complete Active Space SCF (CASSCF);[20–28] better descriptions including dynamic correlation require expensive multi-reference perturbation (MRPT) or multi-reference configuration-interaction (MRCI) calculations, which limit the size of the systems that can be computed.

Nonetheless, previous investigations have shown that DFT methods can give reasonable results if the multi-configurational character is not too large,[29] *i.e.* the contribution of the leading reference structure is at least 75 %. Thus, we were interested in the performance of different DFT methods,* especially with respect to the prediction of experimental data such as NMR spectra or UV-Vis absorption maxima. Furthermore, we used a model system to compare the results of different DFT functionals with accurate MRCI calculations (*vide infra*).

6.1 Prediction of experimental properties using DFT

In general, contrary to what is observed for "single-reference" molecules, pure density functionals outperformed hybrid DFT methods, as HF-type exchange tends to decrease

* The following functionals were tested: GGAs: PBE, BLYP, BP86; mGGAs: M06-L, TPSS, VSXC, τHCTH; Hybrids: PBE0, B3LYP, M06-HF, M06-2X; Double Hybrids: DSD-PBEP86, PBE0-DH, PBEQIDH. For a detailed set of data, please contact the authors.

the robustness towards non-dynamic correlation effects. This agrees with previously reported findings.[30] Of all functionals tested, the PBE functional was exceptionally well suited to reproduce experimental ^{31}P chemical shifts and coupling constants (GIAO method).[31–35] All functionals performed reasonably well in predicting molecular structures (for exemplary results see Table S4) as well as first excitation energies. However, the calculated reaction (free) energies were notoriously bad, mostly due to underestimated stability of the biradical species (Table S5). Inclusion of dispersion (DFT-D3)[36,37] is paramount to produce at least qualitatively correct results. As becomes obvious from the calculated data, neglect of dispersion can to some degree cancel the errors made in the biradicals' energies; however, this is just serendipitous and does not mean that the "uncorrected" DFT functionals should be preferred to the D3 versions.

Table S4: Comparison of selected experimental and calculated structural parameters.

Compound	Parameter	exptl.[a]	PBE-D3/def2-SVP	B3LYP-D3/def2-SVP
**1**	N1–P1	1.718(1) [b]	1.765	1.751
	N1–P2	1.715(1) [b]	1.765	1.750
	P1···P2	2.6186(8) [b]	2.724	2.690
	N1–P1–N1′	80.48(8) [b]	78.97	79.60
	P1–N1–P2	99.44(6) [b]	101.03	100.40
	N1–P1–P2–N1′	180.0 [b]	179.86	180.00
**2**Dmp	N1–P1	1.729(1)	1.753	1.752
	N1–P2	1.649(1)	1.696	1.676
	P1–C49	1.792(2)	1.810	1.801
	P2–N2	1.680(1)	1.720	1.706
	N2–C49	1.430(2)	1.431	1.429
	N3–C49	1.287(2)	1.297	1.287
	P1···P2	2.9437(7)	3.003	2.978
	N1–P1–C49	92.38(6)	92.62	92.70
	N1–P2–N2	94.57(6)	93.35	94.06
	N1–P1–P2–N2	178.5(1)	178.25	177.77

[a] from singe crystal X-ray diffraction data; [b] literature data[9]

Table S5: Selection of computed Gibbs free energies of the reactions **1**+ ^{t}BuNC ⇌ **2**^{t}Bu (1) and **2**^{t}Bu + ^{t}BuNC ⇌ **4**^{t}Bu$_2$ (2). Standard state: *c* = 1 mol/L.

Method	ΔG_r°(1) [kJ/mol]	ΔG_r°(2) [kJ/mol]
B3LYP/def2-SVP	+8.6	+25.8
τHCTH/def2-SVP	−2.0	+18.8
**exptl.** [b]	**−8.9(3)**	**−13.1(3)**
PBE/def2-SVP	−36.1	−18.5
PBE0/def2-SVP	−34.7	−23.9
B3LYP-D3/def2-TZVP//B3LYP-D3/def2-SVP [a]	−37.4	−24.8
DSD-PBEP86/def2-SVP//PBE-D3/def2-SVP [a]	−51.7	−30.0
PBE-D3/def2-TZVP//PBE-D3/def2-SVP [a]	−53.9	−44.4
B3LYP-D3/def2-SVP	−68.1	−44.8
PBE-D3/def2-SVP	−83.5	−61.7
PBE0-D3/def2-SVP	−72.1	−75.8

[a] *method1//method2*: Structure optimizations and estimation of the thermal corrections to Gibbs free energies were done using *method2*; the absolute energies E_{tot} were then recomputed using *method1*.
[b] Experimental values derived from NMR integrals at thermal equilibrium.

6.2 Model system

To gain further insight in the utility of DFT methods as well as to study the mechanism of the photochemical switching process, we performed a series of DFT, CAS and MRCI calculations on the model system **2**H, *i.e.* all organic substituents were replaced by hydrogen atoms.

Firstly, the S_0 and S_1 potential energy surfaces were studied by CAS(8,6) calculations applying the def2-TZVP[38] basis set. The active space was chosen such that all π-type orbitals (A′′ symmetry, point group C_S, Figure S26) of **2**H were included. The biradical **2**H represents the global minimum on the S_0 surface, while the housane-type species **3**H corresponds to a local minimum. Both minima are connected by a single transition state (**TS**), *i.e.* the P-P bond formation/breaking as well as the folding of the ring system occur simultaneously. The electronic excitation of the biradical **2**H to the S_1 surface is

in the visible range of the electromagnetic spectrum (463 nm). Distortion of the ring system leads to a conical intersection (**CI**) between S_1 and S_0 surface which allows radiationless deactivation of the excited state (Figure S27).

Figure S26: CAS(8,6) orbitals of **2**H (left), the transition state (centre), and **3**H (right). The orbital occupation numbers of the CAS reference are given.

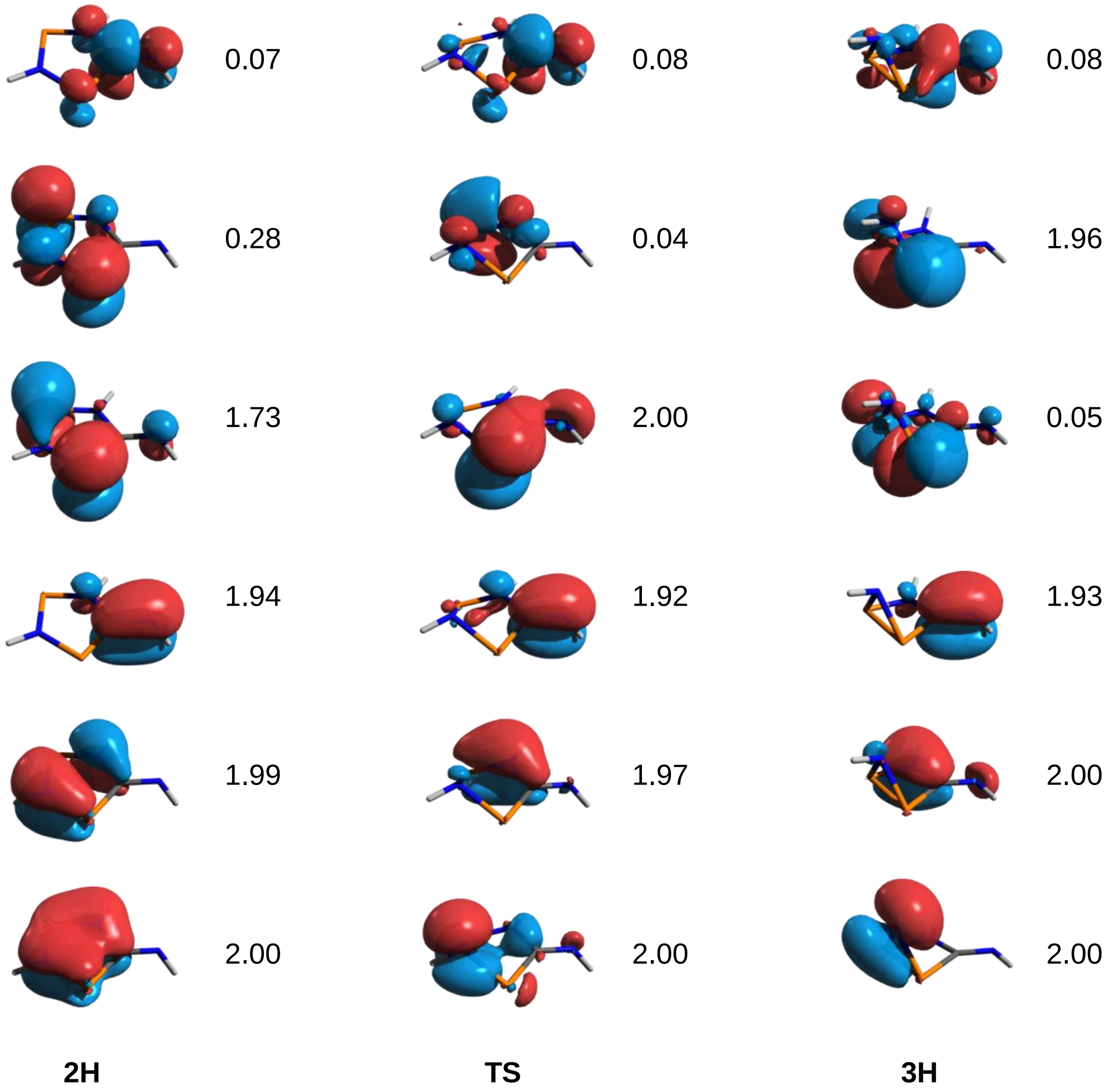

A scan of the ground state PES using DFT methods led to similar results. Likewise, the electronic excitation energy calculated by TD-DFT agreed reasonably with the CAS results (Table S6).

Figure S27: PES at the CAS(8,6)/def2-TZVP level of theory.

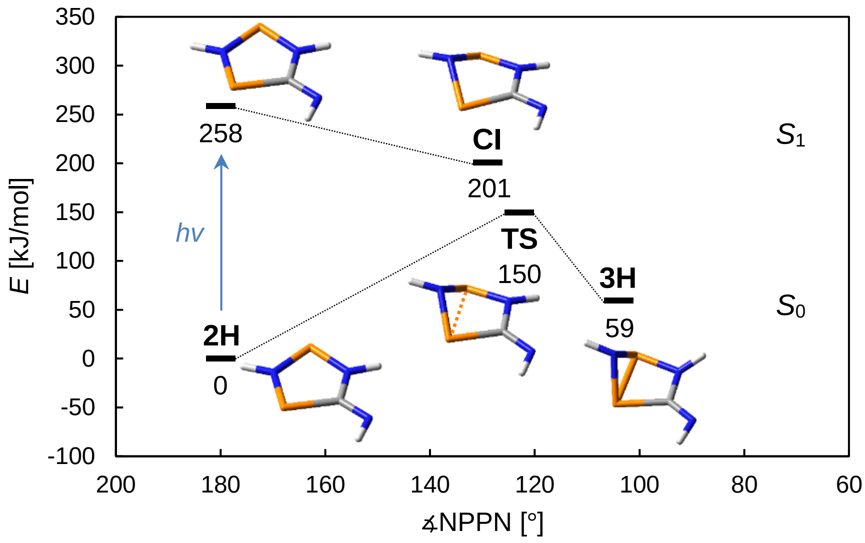

To obtain more reliable estimates of the relative energies discussed above, multi-reference CISD single point calculations (including the *Davidson1* correction for the disconnected quadruples, MRCI+Q) were carried out, using the PBE/def2-TZVP geometries. The CAS(8,6) wave function was chosen as reference (Figure S26). The agreement with the CAS results is reasonable; however, the correlated treatment lowers the first excitation energy by some 30 kJ/mol. Taking the MRCI+Q energies as benchmark values, it becomes clear that most density functionals underestimate the stability of the biradical **2**H (Table S6). Nonetheless, the pure meta-GGA functionals τHCTH,[39] VSXC,[40] and, curiously, the hybrid functional B3LYP[41–46] (as implemented in Gaussian) perform remarkably well.

Table S6: Relative energies (E_{tot}, kJ/mol) of **2**H, **3**H, and the TS structure, as well as the first excitation energy of **2**H. All calculations applied the def2-TZVP basis set. All structures were fully optimized.

Method	**2**H	**3**H	TS	$S_0 \rightarrow S_1$
PBE	0.0	43.8	152.2	210.1
TPSS	0.0	43.6	152.3	212.8
τHCTH	0.0	50.9	156.1	213.2
VSXC	0.0	52.5	156.0	216.4
B3LYP	0.0	56.2	153.9	226.9

Method	**2**H	**3**H	TS	$S_0 \rightarrow S_1$
PBE0	0.0	33.4	150.7	234.5
M06-2X	0.0	33.0	147.0	245.5
DSD-PBEP86	0.0	41.4	153.8	– [b]
PBE0-DH	0.0	29.2	149.0	– [b]
PBEQIDH	0.0	31.5	151.3	– [b]
CAS(8,6)	0.0	59.2	149.7	258.4
**MRCI+Q** [a]	**0.0**	**50.4**	**164.1**	**224.9**

[a] Single point calculation at PBE/def2-TZVP geometry [b] TD-DFT not available for Double Hybrids

Table S7: Overview of the three lowest-lying singlet and triplet states of **2**H.

State	Multiplicity	Irrep	E_{tot} [eV]	λ [nm]
S_0	1	A′	0.0	
T_1	3	A′	0.82	(1508) [a]
S_1	1	A′	2.47	502
T_2	3	A″	3.22	(385) [a]
S_2	1	A″	3.41	364
T_3	3	A′	4.13	(301) [a]

[a] Spin-forbidden transition

The ordering of the (excited) singlet and triplet states of **2**H was investigated by a different set of MRCI+Q calculations. From a preliminary closed-shell HF/def2-TZVP calculation, the six highest-lying occupied MOs and the six lowest-lying improved virtual orbitals (IVOs) were chosen as reference space (*i.e.* formal CAS(6,6) reference including four orbitals of A′′ and two orbitals of A′ symmetry). The three lowest-lying singlet and triplet states were computed. The results show that the singlet biradical is the true electronic ground state (S_0). The first triplet state (T_1) is 79.3 kJ/mol higher in energy and thus in between the ground and first excited singlet state (S_1, 238.5 kJ/mol). Note that the energy of the S_1 state differs somewhat from the (more accurate) results described above; this is due to the CAS(6,6) reference that was chosen as a compromise between accuracy and computational effort. The description of the S_1 state is somewhat

less complete in this set of computations as the reference space includes only four of the six orbitals of A′′ symmetry. However, this is more of a technicality and does not influence the overall results. More excitation energies can be found in Table S7.

6.3 Biradical character

The wave function of the biradical **2** must be described by at least two determinants, since the frontier orbitals are nearly degenerate. In case of the singlet ground state, the CAS(8,6) wave function (including all π-type orbitals in the active space) is dominated by the contributions of two configuration state functions (CSFs), namely the two configurations where either the formal HOMO (π_4) or LUMO (π_5) are doubly occupied:

$$\Psi_{\mathrm{CAS(8,6)}} \approx 0.910|\dots\pi_1^2\pi_2^2\pi_3^2\pi_4^2\rangle - 0.367|\dots\pi_1^2\pi_2^2\pi_3^2\pi_5^2\rangle$$

Hence, the dominant configuration has a relative weight of $w_1 = c_1^2 = 83$ %. According to the scale of Miliordos *et a*l,[47] this corresponds to a biradical character of

$$\beta = \frac{2c_2^2}{c_1^2 + c_2^2} = 28\ \%$$

Note that the value of the biradical character is strongly dependent on the method used. If we consider an MRCI+Q calculation using the same CAS(8,6) reference, the inclusion of substituted determinants describing dynamic correlation lowers the relative weight of each single reference determinant. While the nature of the two dominant configurations remains unchanged and all other determinants have relative weights below 1 %, the overall constitution of the wave function changes significantly:

$$\Psi_{\mathrm{MRCI}} \approx 0.879|\dots\pi_1^2\pi_2^2\pi_3^2\pi_4^2\rangle - 0.206|\dots\pi_1^2\pi_2^2\pi_3^2\pi_5^2\rangle$$

Hence, the biradical character is calculated as $\beta = 10$ %. The values are therefore only comparable if they have been computed using the same methodology.

6.4 Electronic structure of S_0 and S_1 state

As discussed before, the dominant configuration of the ground state CAS/MRCI expansion contains two electrons in orbital π_4 (Figure S28 left), which is transannularly antibonding between the two P atoms. Despite contributions from the second CSF, which places two electrons into orbital π_5 (net orbital occupations: π_4 1.73 *vs.* π_5 0.28), the interaction between those atoms can be classified as mostly antibonding in the ground state.

Figure S28: Schematic depiction of the relevant state-specific CAS(8,6) orbitals of **2**H.

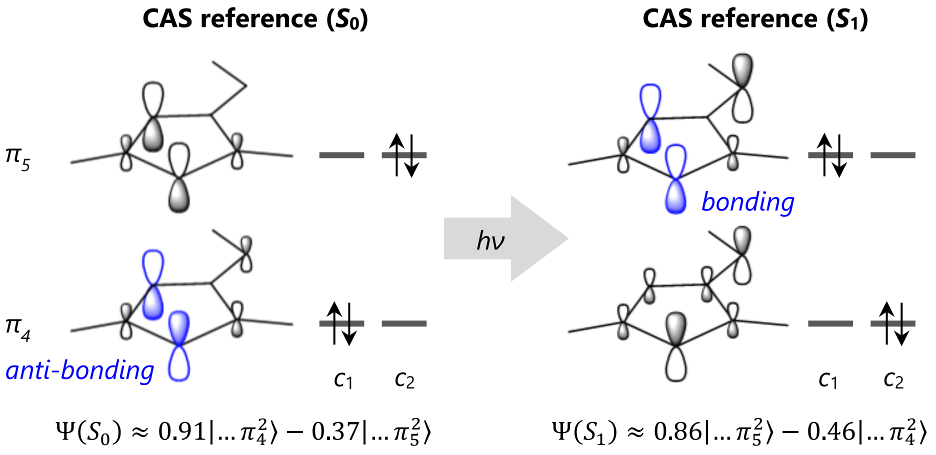

The first excited singlet state can be described in several ways, depending on the method and reference wave function used: In a state-specific CAS calculation, the orbitals are variationally optimized for the first excited singlet state (*i.e.* the 2nd root of the singlet CI matrix), leading to a unique set of orbitals which optimally describes the electronic structure of that state (Figure S28 right). Comparable to the ground state, the excited-state CAS expansion contains two dominant CSFs. They are similar to the ground-state CSFs but with approximately interchanged coefficients. Hence, the configuration that contains two electrons in the transannularly bonding orbital π_5 now has the largest weight and results in a *bonding* interaction between the P atoms.

While state-specific CAS calculations return optimal orbital shapes for each state of interest, these (approximate) wave functions, though easy to interpret, are not

necessarily orthogonal as they should be. This may be remedied by a state-averaged CAS calculation, yielding orbitals that are equally adequate to describe all states in question; however, these orbitals may be difficult to analyse in a chemically intuitive way.

A different approach is to use the occupied orbitals and improved virtual orbitals (IVOs) of a closed-shell HF calculation in an MRCI calculation. That is, the CAS reference is generated without re-optimization of the MOs/IVOs, and dynamic correlation is included by a CISD approach. The same orbitals are used for every state, thus making sure that all states are orthogonal to each other. Moreover, the orbitals are chemically interpretable. The drawback of this method is the high computational effort, making it only feasible for small (model) systems.

In case of **2**H, the MRCI results for the ground state draw a very similar picture to the state-specific CAS calculation. The main contribution to the wave function comes from the CSF that places two electrons into the anti-bonding orbital π_4. The S_1 state is mainly described by an open-shell singlet CSF, with one electron residing in orbital π_4 and the other one in orbital π_5. This nicely agrees with the notion that one electron is promoted from the “HOMO” to the “LUMO” upon excitation (Figure S29). However, it is important to note that other reference functions are needed to fully describe both species!

Figure S29: Schematic depiction of the relevant orbitals and configurations of **2**H in an MRCI calculation. Only contributions larger than 1 % are shown.

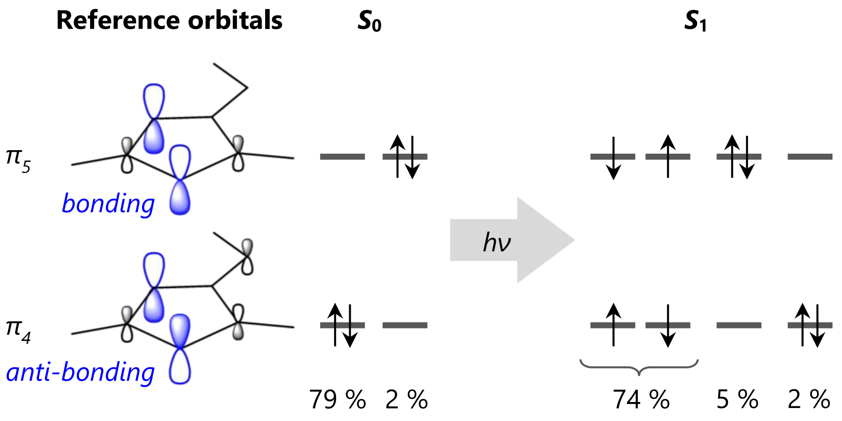

All approaches described in this section give comparable results concerning the energy difference between the S_0 and S_1 states (cf. chapter 6.2).

6.5 NMR data

Chemical shifts and coupling constants were calculated at the PBE-D3/def2-SVP level of theory using the GIAO method.[31–35] The calculated absolute shifts ($\sigma_{calc,X}$) were referenced to the experimental absolute shift of 85 % H_3PO_4 in the gas phase ($\sigma_{ref,1}$ = 328.35 ppm),[48] using PH_3 ($\sigma_{ref,2}$ = 594.45 ppm) as a secondary standard:[49]

$$\delta_{\mathrm{calc,X}} = \left(\sigma_{\mathrm{ref,1}} - \sigma_{\mathrm{ref,2}}\right) - \left(\sigma_{\mathrm{calc,X}} - \sigma_{\mathrm{calc,PH_3}}\right)$$

$$= \sigma_{\mathrm{calc,PH_3}} - \sigma_{\mathrm{calc,X}} - 266.1\ \mathrm{ppm}$$

At the PBE-D3/def2-SVP level of theory, $\sigma_{\mathrm{calc,PH_3}}$ amounts to +617.22 ppm.

Table S8: Experimental (calculated) ^{31}P NMR data of **2**Dmp (AX spin system, THF-d_8).

	δ [ppm]	J [Hz]
P_A (N–*P*–C)	+221.5 (+240.3)	
P_X (N–*P*–N)	+258.1 (+259.1)	J_{AX} = +135 (+163)

Table S9: Experimental (calculated) ^{31}P NMR data of **3**Dmp (AX spin system, THF-d_8).

	δ [ppm]	J [Hz]
P_A (N–*P*–C)	−129.2 (−125.1)	
P_X (N–*P*–N)	−63.4 (−45.7)	J_{AX} = −65 (−19)

Table S10: Experimental (calculated) ^{31}P NMR data of **4**Dmp^{t}Bu (AX spin system, THF-d_8).

	δ [ppm]	J [Hz]
P_A (N–*P*–C)	+188.6 (+201.8)	
P_X (N–*P*–N)	+233.1 (+208.4)	J_{AX} = −31 (−33)

Table S11: Experimental (calculated) ^{31}P NMR data of **2**^{t}Bu (AX spin system, THF-d_8).

	δ [ppm]	*J* [Hz]
P_A (N–*P*–C)	+194.9 (+202.9)	
P_X (N–*P*–N)	+259.9 (+252.0)	J_{AX} = +144 (+151)

Table S12: Experimental (calculated) ^{31}P NMR data of **3**^{t}Bu (AX spin system, THF-d_8).

	δ [ppm]	*J* [Hz]
P_A (N–*P*–C)	−106.6 (−94.9)	
P_X (N–*P*–N)	−60.9 (−51.9)	J_{AX} = −64 (−21)

Table S13: Experimental (calculated) ^{31}P NMR data of **4**^{t}Bu$_2$ (AX spin system, THF-d_8).

	δ [ppm]	*J* [Hz]
P_A (N–*P*–C)	+166.5 (+174.6)	
P_X (N–*P*–N)	+210.6 (+180.5)	J_{AX} = −33 (−31)

6.6 Optimized structures (.xyz files)

6.6.1 PH_3 (PBE-D3/def2-SVP)

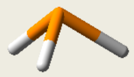

```
4
Phosphane (NMR standard)
P          0.00000        0.00000        0.13355
H          0.00000        1.19789       -0.66776
H          1.03740       -0.59894       -0.66776
H         -1.03740       -0.59894       -0.66776
```

6.6.2 Model system 2H (PBE/def2-TZVP)

```
9
Biradical @ PBE/def2-TZVP geometry
C          0.09589        1.17428        0.00000
N          0.05049        2.46477        0.00000
N         -1.13086        0.46035        0.00000
N          0.43159       -1.36086        0.00000
P          1.49329        0.00000        0.00000
P         -1.23243       -1.21550        0.00000
H          0.83327       -2.29769        0.00000
H          0.99062        2.87138        0.00000
H         -1.96690        1.04750        0.00000
```

6.6.3 Model system 3H (PBE/def2-TZVP)

```
9
Housane @ PBE/def2-TZVP geometry
C          -1.10458         0.43957        -0.26251
N          -2.34510         0.67123        -0.42212
N          -0.14527         0.45574        -1.27030
N           0.61517        -1.43229         0.29109
P           0.00000        -0.00000         1.24063
P           1.33991        -0.00000        -0.47524
H          -2.85556         0.63292         0.46624
H          -0.39943         0.31611        -2.24962
H           1.30131        -1.94418         0.86503
```

6.6.4 Transition state TS (PBE/def2-TZVP)

```
9
TS @ PBE/def2-TZVP geometry
C          -1.13124         0.23374         0.05693
N          -2.35965         0.62545        -0.02101
N          -0.43977         0.12566        -1.20387
N           1.22226        -0.96403         0.30283
P          -0.00000        -0.00000         1.44087
P           1.20201         0.00000        -1.03322
H          -2.76924         0.72564         0.91631
H          -0.92477         0.47412        -2.03150
H           2.15402        -1.02617         0.72322
```

6.6.5 Conical intersection (CAS(8,6)/def2-TZVP)

```
9
Conical Intersection @ CAS(8,6)/def2-TZVP geometry
C          -1.09335        -0.25875         0.07714
N          -2.30030        -0.65404         0.10901
N          -0.08444        -1.25549         0.06799
N           1.24520         0.73916         0.51905
P          -0.36213         1.38868        -0.17556
P           1.41962        -0.69618        -0.18410
H          -2.93813         0.11932         0.08085
H          -0.36785        -2.20130        -0.06633
H           2.01652         1.36800         0.45643
```

6.6.6 tBuNC (PBE-D3/def2-SVP)

```
15
tBuNC
C           0.00000         1.46357        -0.73634
C           0.00000         0.00000        -0.25481
H          -0.89822         1.99681        -0.36870
H           0.89822         1.99681        -0.36870
H           0.00000         1.49411        -1.84369
C           1.26749        -0.73178        -0.73634
C          -1.26749        -0.73178        -0.73634
H           1.28018        -1.77629        -0.36870
H           1.29394        -0.74706        -1.84369
```

```
H           2.17840       -0.22053       -0.36870
H          -1.29394       -0.74706       -1.84369
H          -1.28018       -1.77629       -0.36870
H          -2.17840       -0.22053       -0.36870
C           0.00000        0.00000        2.37162
N           0.00000        0.00000        1.18523
```

6.6.7 DmpNC (PBE-D3/def2-SVP)

```
19
DmpNC
C           0.00000        1.24571       -0.23889
C           0.00000        0.00000        0.44148
C          -0.00000       -1.24571       -0.23889
C          -0.00000       -1.21630       -1.64399
C          -0.00000        0.00000       -2.34169
C           0.00000        1.21630       -1.64399
H          -0.00000       -2.16899       -2.19548
H          -0.00000        0.00000       -3.44206
H           0.00000        2.16899       -2.19548
N           0.00000        0.00000        1.82414
C           0.00000        0.00000        3.01526
C          -0.00000       -2.53336        0.53709
H           0.88735       -2.60279        1.20067
H          -0.88735       -2.60279        1.20067
H          -0.00000       -3.40837       -0.13967
C           0.00000        2.53336        0.53709
H          -0.88735        2.60279        1.20067
H           0.88735        2.60279        1.20067
H           0.00000        3.40837       -0.13967
```

6.6.8 Biradical 1 (PBE-D3/def2-SVP)

```
102
P2N2 biradical
N           1.12217       -0.00001       -0.01590
N          -1.12217        0.00001       -0.01590
P          -0.00020       -1.36211       -0.01451
P           0.00020        1.36211       -0.01451
C          -2.52566       -0.00218       -0.02302
C          -3.24509       -1.16088        0.38976
C          -3.24533        1.15397       -0.44301
C          -4.65178       -1.14406        0.36796
C          -4.65178        1.12771       -0.44955
C          -5.36232       -0.01136       -0.04965
H          -5.18642       -2.04768        0.69907
H          -5.18615        2.02917       -0.78704
H          -6.46229       -0.01559       -0.06117
C           2.52566        0.00218       -0.02302
C           3.24533       -1.15397       -0.44301
C           3.24509        1.16088        0.38976
C           4.65178       -1.12771       -0.44955
C           4.65178        1.14406        0.36796
C           5.36232        0.01136       -0.04965
H           5.18615       -2.02917       -0.78704
H           5.18642        2.04768        0.69907
H           6.46229        0.01559       -0.06117
```

C	2.54230	2.38351	0.88274
C	2.38727	3.51412	0.04015
C	2.04476	2.40858	2.21542
C	1.73846	4.65367	0.55030
C	1.40130	3.56628	2.67857
C	1.23253	4.69891	1.85961
H	1.60956	5.52886	-0.10568
H	1.00908	3.58045	3.70869
C	2.53932	-2.38841	-0.90002
C	2.40070	-3.49602	-0.02450
C	2.01959	-2.44883	-2.22222
C	1.73823	-4.64672	-0.48909
C	1.36833	-3.61892	-2.64248
C	1.21062	-4.72718	-1.78866
H	1.61762	-5.50239	0.19372
H	0.95929	-3.66158	-3.66526
C	-2.53932	2.38841	-0.90002
C	-2.40070	3.49602	-0.02450
C	-2.01959	2.44883	-2.22222
C	-1.73823	4.64672	-0.48909
C	-1.36833	3.61892	-2.64248
C	-1.21062	4.72718	-1.78866
H	-1.61762	5.50239	0.19372
H	-0.95929	3.66158	-3.66526
C	-2.54230	-2.38351	0.88274
C	-2.04476	-2.40858	2.21542
C	-2.38727	-3.51412	0.04015
C	-1.40130	-3.56628	2.67857
C	-1.73846	-4.65367	0.55030
C	-1.23253	-4.69891	1.85961
H	-1.00908	-3.58045	3.70869
H	-1.60956	-5.52886	-0.10568
C	-2.90038	3.41963	1.39619
H	-2.73177	4.37297	1.93314
H	-3.98088	3.17771	1.44421
H	-2.37189	2.61565	1.95102
C	-2.12974	1.25530	-3.13558
H	-3.17529	0.89408	-3.21247
H	-1.76117	1.48954	-4.15254
H	-1.53158	0.40566	-2.74100
C	-0.48357	5.96029	-2.26263
H	0.56922	5.72563	-2.52643
H	-0.95388	6.38444	-3.17419
H	-0.47037	6.75068	-1.48732
C	-2.82564	-3.47188	-1.40196
H	-2.79643	-4.47895	-1.86124
H	-3.84635	-3.05963	-1.52070
H	-2.14331	-2.81395	-1.98332
C	-2.16775	-1.19151	3.09583
H	-1.54955	-0.35774	2.69761
H	-3.21112	-0.81832	3.13582
H	-1.82873	-1.40449	4.12764
C	-0.51468	-5.91883	2.37941
H	0.53555	-5.67838	2.64880
H	-0.99657	-6.31439	3.29772
H	-0.49329	-6.73233	1.62854
C	0.48357	-5.96029	-2.26263
H	-0.56922	-5.72563	-2.52643
H	0.95388	-6.38444	-3.17419
H	0.47037	-6.75068	-1.48732

```
C          0.51468       5.91883       2.37941
H         -0.53555       5.67838       2.64880
H          0.99657       6.31439       3.29772
H          0.49329       6.73233       1.62854
C          2.16775       1.19151       3.09583
H          3.21112       0.81832       3.13582
H          1.82873       1.40449       4.12764
H          1.54955       0.35774       2.69761
C          2.12974      -1.25530      -3.13558
H          3.17529      -0.89408      -3.21247
H          1.76117      -1.48954      -4.15254
H          1.53158      -0.40566      -2.74100
C          2.90038      -3.41963       1.39619
H          2.73177      -4.37297       1.93314
H          3.98088      -3.17771       1.44421
H          2.37189      -2.61565       1.95102
C          2.82564       3.47188      -1.40196
H          2.79643       4.47895      -1.86124
H          3.84635       3.05963      -1.52070
H          2.14331       2.81395      -1.98332
```

6.6.9 Biradical 2Dmp (PBE-D3/def2-SVP)

```
121
Biradical Dmp
C         -5.95728      -0.48562       3.18325
C         -4.46120      -0.65588       3.09826
C         -3.89348      -1.72738       2.38754
C         -3.59227       0.24343       3.74237
C         -5.25041      -3.33989      -1.91111
C         -2.50363      -1.92828       2.32044
C         -4.76393      -1.04424      -0.95217
C         -2.19699       0.07185       3.72151
C         -4.63398      -1.96679      -2.00416
C         -1.93427      -3.10126       1.56896
C         -4.28299       1.16141       0.18230
C         -1.64953      -1.02705       3.00556
C         -1.30160       1.04218       4.45255
C         -4.12988       0.21075      -0.97804
C          0.24717      -1.94013       4.30822
C          0.39174      -4.96244      -0.51006
C         -3.89073      -1.57962      -3.13394
C         -0.18578      -1.32049       3.11681
C          1.59355      -2.25546       4.51210
C         -3.35320       0.55747      -2.11118
C          1.76334      -5.11081      -2.62816
C          1.27100      -4.34431      -1.56307
C         -3.26279      -0.32474      -3.21834
C          0.77300      -1.01849       2.11455
C          2.58451      -4.54122      -3.61387
C         -2.67669       1.88941      -2.13955
C         -3.28847       2.95695      -2.82348
C          1.59428      -2.95153      -1.47998
C          2.53243      -1.92352       3.52924
C         -0.97917       4.67483       1.26232
C          0.55257      -1.12157      -0.36311
C         -2.50974       0.06812      -4.46476
C          2.15246      -1.29417       2.32762
```

C	2.91825	-3.18667	-3.52136
C	-2.73285	4.24203	-2.80560
C	-1.46646	2.13522	-1.43518
C	2.44787	-2.36976	-2.47264
C	2.72280	1.57348	1.96303
C	-1.55218	4.47313	-2.09181
C	-0.89480	3.43384	-1.39962
C	2.88788	-0.92979	-2.42902
C	0.33495	4.43737	0.55909
C	3.24133	-0.86682	1.40047
C	3.52024	0.51581	1.24575
C	0.38086	3.77760	-0.69522
C	1.53922	4.87957	1.13960
C	3.85003	-3.31602	0.96761
C	4.08181	-1.83648	0.78483
C	4.60210	0.90570	0.43130
C	1.62423	3.57040	-1.34917
C	2.78069	4.69574	0.50856
C	1.68178	2.89351	-2.69404
C	5.15293	-1.39675	-0.00994
C	2.79886	4.03151	-0.73216
C	5.42595	-0.03083	-0.21152
C	4.06359	5.17314	1.14064
C	6.54671	0.40659	-1.12009
H	-6.36603	-0.99135	4.08464
H	-6.24482	0.58245	3.25272
H	-6.47102	-0.92500	2.30501
H	-6.16998	-3.33610	-1.29262
H	-4.55157	-2.44251	1.86898
H	-4.01236	1.10394	4.28836
H	-5.50549	-3.74583	-2.90990
H	-5.37821	-1.30571	-0.07696
H	-4.54303	-4.05719	-1.44021
H	-5.18604	0.92527	0.77504
H	-1.88342	1.88445	4.87354
H	-1.44927	-3.82272	2.25983
H	-2.72216	-3.63803	1.00636
H	-0.50555	-2.17955	5.07458
H	0.36369	-6.06525	-0.60644
H	-4.34746	2.21537	-0.15205
H	-0.75585	0.55000	5.28347
H	0.73986	-4.69602	0.50914
H	-3.41823	1.08735	0.87676
H	-1.14578	-2.79415	0.85490
H	-0.52279	1.46447	3.78260
H	-3.79506	-2.27638	-3.98239
H	1.49773	-6.17900	-2.68072
H	1.91445	-2.74485	5.44395
H	-0.65055	-4.58689	-0.58257
H	-4.22958	2.76180	-3.35941
H	2.97110	-5.15521	-4.44129
H	-1.64485	5.33911	0.67373
H	-1.54126	3.72859	1.41023
H	-0.82342	5.13659	2.25599
H	2.29534	1.19993	2.91359
H	-2.46703	-0.76994	-5.18618
H	-3.22383	5.06626	-3.34451
H	1.87488	1.94167	1.34323
H	3.60116	-2.12547	3.69285
H	-2.98408	0.93392	-4.97176

```
H          3.57987       -2.73376       -4.27790
H          3.90305       -0.82021       -2.86002
H         -1.46810        0.36948       -4.23012
H          3.34718        2.45803        2.18842
H          2.20655       -0.27494       -3.01809
H         -1.10248        5.47737       -2.06901
H          1.50104        5.38766        2.11687
H          2.77413       -3.53057        1.10061
H          4.39220       -3.70639        1.85546
H          2.90865       -0.52357       -1.40162
H          4.81016        1.98121        0.31194
H          4.20707       -3.87915        0.08391
H          1.29662        1.85348       -2.63273
H          1.05179        3.42108       -3.43964
H          4.74485        4.32329        1.36069
H          3.87805        5.70934        2.09128
H          5.78562       -2.15196       -0.50405
H          2.71794        2.84955       -3.07975
H          3.76037        3.87241       -1.24678
H          4.61780        5.85656        0.46447
H          6.81581        1.46881       -0.95770
H          6.25508        0.29797       -2.18753
H          7.45786       -0.20803       -0.97336
N          1.15515       -2.27039       -0.34902
N          0.33034       -0.43477        0.87295
N         -0.79587        1.03273       -0.78715
P         -0.19349       -0.27905       -1.78110
P         -0.55791        1.03819        0.89256
```

6.6.10 Housane 3Dmp (PBE-D3/def2-SVP)

```
121
Housane Dmp
C          3.45103       -2.76421       -4.67657
C          2.43144       -2.97503       -3.58613
C          2.74264       -3.73567       -2.44448
C          1.15952       -2.38027       -3.65388
C          6.15688        2.90243        1.01610
C          1.83708       -3.89456       -1.38017
C          4.28104        2.83112       -0.69537
C          0.21703       -2.51853       -2.61833
C          4.72485        3.12689        0.60484
C          2.23330       -4.71329       -0.17672
C          2.49764        2.69877       -2.49236
C          0.56639       -3.26438       -1.46326
C         -1.15518       -1.91368       -2.76332
C          2.93769        2.99471       -1.08020
C         -0.97385       -4.80169       -0.27208
C          3.29129       -0.44614       -1.13448
C          3.78405        3.62188        1.52725
C         -0.46462       -3.49329       -0.40840
C         -2.00588       -5.10475        0.62147
C          2.00381        3.47089       -0.12560
C          5.25249       -0.54301        0.46996
C          3.89012       -0.77371        0.20697
C          2.43616        3.81256        1.18497
C         -1.01752       -2.46325        0.40299
C          5.83405       -0.90241        1.69373
```

C	0.59668	3.73713	-0.55707
C	0.28414	5.06562	-0.90602
C	3.10430	-1.39096	1.22282
C	-2.56959	-4.07719	1.38623
C	-1.39323	1.41474	-3.58967
C	0.85746	-0.86935	0.74065
C	1.45549	4.33606	2.20250
C	-2.09223	-2.75744	1.28881
C	5.04965	-1.51841	2.67937
C	-0.97527	5.42370	-1.39776
C	-0.39770	2.71906	-0.69331
C	3.68686	-1.78086	2.46044
C	-4.67227	-1.75337	0.41217
C	-1.93473	4.42021	-1.56604
C	-1.67072	3.07779	-1.23106
C	2.83576	-2.44737	3.50742
C	-2.62941	1.31164	-2.73221
C	-2.75573	-1.68012	2.08135
C	-4.01207	-1.18188	1.64053
C	-2.74149	2.08581	-1.54710
C	-3.70298	0.48698	-3.11003
C	-0.86738	-1.74770	3.78026
C	-2.15038	-1.15879	3.25421
C	-4.61020	-0.12929	2.35203
C	-3.93103	2.03831	-0.77341
C	-4.89325	0.42494	-2.36170
C	-4.05686	2.84398	0.49411
C	-2.78053	-0.09460	3.92545
C	-4.98542	1.21206	-1.20042
C	-4.00162	0.44536	3.48403
C	-6.02304	-0.47856	-2.78692
C	-4.63585	1.61856	4.18687
H	4.09966	-3.65297	-4.80893
H	2.97216	-2.53499	-5.64893
H	4.11831	-1.90966	-4.42974
H	6.82057	2.77847	0.13807
H	3.73519	-4.20874	-2.36729
H	0.88215	-1.79791	-4.54793
H	6.54292	3.74357	1.62682
H	5.00438	2.46198	-1.44033
H	6.24342	1.98093	1.63033
H	3.33637	2.31567	-3.10434
H	-1.33531	-1.58203	-3.80274
H	1.80457	-5.73687	-0.21277
H	3.33400	-4.81505	-0.11504
H	-0.55039	-5.58924	-0.91295
H	4.06765	-0.07861	-1.83179
H	2.09262	3.60753	-2.98373
H	-1.95069	-2.63370	-2.48424
H	2.81143	-1.33803	-1.58073
H	1.68377	1.94524	-2.51308
H	1.87312	-4.23096	0.75203
H	-1.28228	-1.03147	-2.10512
H	4.11174	3.86805	2.55047
H	5.86777	-0.07521	-0.31440
H	-2.38140	-6.13519	0.70877
H	2.50869	0.33352	-1.07360
H	1.07453	5.82418	-0.79467
H	6.90374	-0.71576	1.87383
H	-1.19275	2.46811	-3.87302

```
H          -0.49618        1.05935       -3.04143
H          -1.49274        0.81704       -4.51531
H          -4.84131       -2.84426        0.50752
H           1.95522        4.56631        3.16287
H          -1.19863        6.46641       -1.66701
H          -4.03394       -1.61027       -0.48445
H          -3.39338       -4.28638        2.08578
H           0.93909        5.24979        1.84589
H           5.50031       -1.81324        3.64034
H           2.20717       -3.24461        3.05939
H           0.65715        3.58841        2.40311
H          -5.64391       -1.26490        0.21490
H           3.45563       -2.88750        4.31203
H          -2.92352        4.66014       -1.98702
H          -3.61471       -0.10928       -4.03295
H          -0.07174       -1.77505        3.01118
H          -1.01754       -2.80104        4.09690
H           2.13236       -1.72866        3.97898
H          -5.57848        0.26284        2.00205
H          -0.48988       -1.17749        4.65038
H          -3.30993        2.50502        1.24457
H          -3.86618        3.92194        0.32411
H          -5.79389       -1.54081       -2.55352
H          -6.20177       -0.42191       -3.87971
H          -2.30059        0.32102        4.82625
H          -5.06102        2.73055        0.94531
H          -5.91224        1.18576       -0.60482
H          -6.96838       -0.22357       -2.26943
H          -5.73892        1.51930        4.23243
H          -4.41990        2.56555        3.64640
H          -4.25444        1.73417        5.22000
N           1.76942       -1.73466        0.97152
N          -0.46474       -1.14712        0.39908
N          -0.12241        1.36054       -0.36750
P           0.83034        0.99450        1.15345
P          -1.27623        0.45608        0.70309
```

6.6.11 Adduct 4Dmp^{t}Bu (PBE-D3/def2-SVP)

```
136
Adduct Dmp tBu
C          -4.77613        2.38253        3.59889
C          -2.27276        2.68682        3.27855
C           0.21081        3.11734        2.98371
C          -0.54978        4.62022        0.03510
C           4.45737        4.82449        0.76803
C          -3.36030        1.86279        3.61718
C           1.96033        4.71582        0.32340
C          -0.95132        2.20239        3.26853
C           0.84790        4.26868       -0.40602
C           3.27689        4.41987       -0.07716
C          -3.09405        0.53159        3.98678
C           1.37831        0.38616        4.82794
C          -0.72153        0.84194        3.58081
C           2.69712       -0.06851        4.93055
C          -1.79048        0.00525        3.98744
C           3.45725        3.72900       -1.28637
C           1.05483        3.47957       -1.57296
```

C	0.67494	0.31659	3.60859
C	-0.57223	3.72119	-3.43070
C	-3.60723	2.29914	-0.05556
C	2.37312	3.26890	-2.05888
C	-0.14988	2.98594	-2.30554
C	-1.74645	3.40171	-4.11873
C	3.31396	-0.63652	3.81140
C	-1.51956	-1.40957	4.43533
C	1.31518	-0.23841	2.47017
C	4.37162	0.66143	0.53655
C	-0.92944	1.87740	-1.84883
C	-2.54528	2.36399	-3.62776
C	2.63909	-0.74574	2.57795
C	-3.99001	1.07778	-0.84783
C	-2.17790	1.61025	-2.49470
C	2.65025	2.54276	-3.34894
C	-5.12272	0.33475	-0.48408
C	-3.22014	0.66556	-1.97447
C	4.17739	-0.83373	0.54530
C	3.34125	-1.48419	1.48078
C	-0.14324	-1.22145	0.65216
C	1.34202	-0.31384	-1.34545
C	-5.51671	-0.81990	-1.18609
C	4.86758	-1.61111	-0.40762
C	-3.60123	-0.49565	-2.69685
C	-6.73614	-1.59828	-0.76159
C	3.25583	-2.90650	1.49425
C	2.44324	-3.61266	2.55014
C	-4.73319	-1.22162	-2.27736
C	-2.80857	-1.00955	-3.87151
C	4.77022	-3.00915	-0.44189
C	3.96339	-3.63804	0.52770
C	2.12707	-1.42915	-3.35373
C	3.52003	-1.50669	-3.99759
C	1.10996	-0.86390	-4.36362
C	5.49863	-3.82564	-1.48031
C	1.69434	-2.81955	-2.86314
H	-4.81403	3.46066	3.34977
H	-2.45505	3.74371	3.02417
H	-0.11734	4.16796	2.88202
H	-0.53599	5.37874	0.84038
H	4.23570	5.71726	1.38531
H	1.79567	5.31505	1.23339
H	-5.27534	2.24050	4.57990
H	-5.38989	1.84106	2.84785
H	0.96646	3.06635	3.79387
H	5.35104	5.04017	0.14942
H	-1.14963	5.01429	-0.80961
H	-1.09485	3.73205	0.41457
H	0.74442	2.83462	2.05138
H	4.73492	4.00440	1.46561
H	0.86700	0.82116	5.70033
H	3.24032	0.01011	5.88444
H	-3.92926	-0.12186	4.28785
H	0.04881	4.57171	-3.75155
H	-4.40905	2.58688	0.64974
H	-3.38726	3.16395	-0.71279
H	4.48081	3.54225	-1.65061
H	-2.05686	3.97972	-5.00150
H	-2.69130	2.10509	0.54164

H	-0.83392	-1.42766	5.30754
H	3.88288	1.15935	1.39448
H	5.45123	0.91849	0.55851
H	4.34204	-1.02403	3.87721
H	-2.45419	-1.92725	4.72555
H	3.95641	1.10754	-0.39104
H	3.57108	2.93010	-3.82738
H	-5.72029	0.67351	0.37865
H	-1.02609	-1.99332	3.63031
H	-3.51140	2.13451	-4.10276
H	2.80169	1.46054	-3.14488
H	1.81363	2.62797	-4.06700
H	-7.64496	-0.96097	-0.76068
H	2.79736	-3.35331	3.56862
H	5.51114	-1.09647	-1.13927
H	1.37601	-3.31103	2.49739
H	-6.62471	-1.99138	0.27182
H	-2.21976	-0.21581	-4.36706
H	-2.09036	-1.78727	-3.53062
H	-6.92447	-2.45993	-1.43069
H	3.85117	-0.50266	-4.32935
H	1.41293	0.14311	-4.71052
H	2.50492	-4.71114	2.43320
H	-5.01244	-2.13179	-2.83233
H	0.10192	-0.78351	-3.91215
H	4.26102	-1.88935	-3.27024
H	-3.46811	-1.48157	-4.62620
H	2.38745	-3.18157	-2.07931
H	3.89276	-4.73804	0.54066
H	6.25174	-3.22125	-2.02222
H	0.66936	-2.80579	-2.44027
H	6.01659	-4.69477	-1.02588
H	3.50871	-2.18101	-4.87710
H	1.03978	-1.53136	-5.24589
H	4.79360	-4.23332	-2.23654
H	1.69621	-3.54322	-3.70285
N	-0.51442	1.02410	-0.78834
N	0.65473	-0.21738	1.18642
N	-0.49692	-2.31873	1.22849
N	2.23156	-0.47821	-2.21795
P	1.11924	1.03346	-0.01233
P	-0.55430	-0.76019	-1.13813
C	-1.36363	-3.20020	0.53194
C	-2.76793	-2.97005	0.57369
C	-0.84375	-4.32808	-0.16044
C	-3.62257	-3.84643	-0.11663
C	-1.73809	-5.17001	-0.85007
C	-3.11789	-4.93604	-0.83839
H	-4.70652	-3.65888	-0.08540
H	-1.32993	-6.03194	-1.40194
H	-3.80012	-5.60635	-1.38284
C	0.62334	-4.66166	-0.12734
H	0.85826	-5.29120	0.75703
H	0.92552	-5.23291	-1.02671
H	1.26215	-3.76495	-0.05340
C	-3.31588	-1.81447	1.36121
H	-2.98458	-1.86726	2.41387
H	-2.96689	-0.83670	0.97322
H	-4.42041	-1.80295	1.33997

6.6.12 Biradical 2^{t}Bu (PBE-D3/def2-SVP)

```
117
Biradical tBu
C          -5.49985        0.40298        3.41176
C          -4.21304       -0.32224        3.10580
C          -4.15878       -1.28848        2.08497
C          -3.04078       -0.06784        3.83777
C          -5.88277       -0.28222       -2.25623
C          -2.98072       -1.99416        1.78349
C          -4.45362        1.06813       -0.65414
C          -1.83833       -0.75170        3.57651
C          -4.75587        0.67686       -1.96762
C          -2.97372       -3.06290        0.72341
C          -3.10019        2.30837        1.07560
C          -1.80767       -1.71077        2.53104
C          -0.61420       -0.47799        4.41683
C          -3.35984        1.90249       -0.35490
C          -0.29446       -3.60290        3.12609
C          -3.95528        1.18451       -3.00810
C          -0.55982       -2.49903        2.28824
C           0.86108       -4.37426        2.96422
C          -2.53444        2.34920       -1.41570
C          -2.85381        2.02004       -2.75993
C           0.37171       -2.18500        1.26694
C          -1.35885        3.22022       -1.10840
C          -1.47362        4.61223       -1.28395
C           1.78273       -4.03933        1.96379
C           1.23372        3.62550        2.72211
C          -0.13246       -1.26790       -1.00659
C          -2.02367        2.54406       -3.90484
C           1.56323       -2.94448        1.10838
C          -0.42952        5.47586       -0.93043
C          -0.14767        2.69739       -0.57436
C           3.29133       -0.57173        1.49890
C           0.74225        4.95004       -0.37661
C           0.90760        3.56122       -0.18602
C           2.37429        3.20763        1.82643
C           2.58653       -2.57893        0.08373
C           3.37482       -1.41473        0.25262
C           2.19814        3.10708        0.42220
C           3.64035        2.93141        2.37611
C           2.03778       -4.71393       -1.18679
C           2.78447       -3.41246       -1.04938
C           4.28554       -1.05467       -0.75930
C           3.28497        2.73136       -0.40957
C           4.73892        2.58322        1.57258
C           3.10785        2.60133       -1.90061
C           3.69614       -3.00797       -2.03829
C           4.53639        2.48922        0.18351
C           4.43941       -1.81743       -1.92633
C           6.08500        2.27350        2.17648
C           5.35780       -1.36759       -3.03466
H          -6.22288       -0.26167        3.93116
H          -5.32975        1.28252        4.06292
H          -6.00064        0.75468        2.48604
H          -6.62031       -0.30912       -1.43016
H          -5.06839       -1.51308        1.50429
H          -3.05964        0.68627        4.64144
```

H	-6.41777	-0.02060	-3.19125
H	-5.08572	0.71152	0.17384
H	-5.49298	-1.31551	-2.38622
H	-4.03991	2.31958	1.65986
H	-0.77321	0.39087	5.08415
H	-2.81351	-4.06368	1.17818
H	-3.93429	-3.08734	0.17293
H	-1.02364	-3.84801	3.91325
H	-2.63400	3.31043	1.14424
H	-0.35290	-1.35241	5.04854
H	-2.42291	1.58966	1.58424
H	-2.14490	-2.92431	-0.00456
H	0.28082	-0.27223	3.79362
H	-4.18825	0.91189	-4.05037
H	1.05071	-5.23209	3.62695
H	-2.41607	5.01123	-1.68842
H	0.91802	4.66978	2.51939
H	0.33389	2.99575	2.56291
H	1.51587	3.55308	3.78992
H	2.78294	-1.09841	2.32842
H	-2.38378	2.14714	-4.87331
H	-0.53434	6.56166	-1.07519
H	2.74200	0.37561	1.31299
H	2.71050	-4.61885	1.84628
H	-2.04687	3.65190	-3.95778
H	-0.95885	2.25254	-3.78938
H	4.30185	-0.27836	1.84429
H	1.56779	5.61614	-0.08309
H	3.77174	3.00364	3.46800
H	0.96890	-4.57202	-0.93644
H	2.44251	-5.48942	-0.50300
H	4.88922	-0.14380	-0.62370
H	2.11382	-5.11136	-2.21766
H	2.42675	1.75716	-2.14384
H	2.65378	3.51296	-2.34009
H	6.25036	1.17541	2.23680
H	6.17755	2.67683	3.20361
H	3.83027	-3.64528	-2.92794
H	4.07467	2.41227	-2.40515
H	5.38549	2.22350	-0.46741
H	6.91175	2.68769	1.56504
H	6.16304	-0.70687	-2.65642
H	4.79572	-0.79244	-3.80240
H	5.82875	-2.22599	-3.55424
N	-0.44420	-2.45682	-1.40318
N	0.14955	-1.05928	0.39291
N	0.00141	1.26687	-0.45848
P	0.00087	0.30450	-1.92735
P	0.09223	0.52049	1.05795
C	-0.86301	-2.74105	-2.78730
C	-2.14465	-1.95125	-3.13695
C	0.25971	-2.42063	-3.79403
C	-1.19677	-4.24267	-2.86434
H	-2.96239	-2.21887	-2.43813
H	-1.99358	-0.85503	-3.06536
H	-2.47587	-2.18260	-4.16967
H	1.19224	-2.95100	-3.51912
H	-0.03366	-2.72095	-4.82076
H	0.49533	-1.33530	-3.82184
H	-1.59094	-4.50607	-3.86665

```
H          -0.30156       -4.86221       -2.66964
H          -1.96265       -4.50286       -2.10679
```

6.6.13 Housane 3^{t}Bu (PBE-D3/def2-SVP)

```
117
Housane tBu
C          -4.23237       -1.15958        4.23838
C          -3.06069       -1.72541        3.47543
C          -3.23017       -2.82338        2.61146
C          -1.77483       -1.17222        3.59321
C          -6.46768        1.20076       -1.59000
C          -2.16825       -3.34877        1.85595
C          -4.59891        1.52790        0.09384
C          -0.68402       -1.65806        2.84781
C          -5.13945        1.78295       -1.17892
C          -2.38541       -4.54160        0.95923
C          -2.80037        1.71371        1.85633
C          -0.89245       -2.73430        1.94998
C           0.68446       -1.05295        3.02827
C          -3.33931        2.01814        0.48445
C           0.80908       -4.51324        1.48306
C          -4.37807        2.55449       -2.07542
C           0.26280       -3.25352        1.16352
C           1.95087       -5.00028        0.83615
C          -2.59165        2.80377       -0.43446
C          -3.11727        3.07159       -1.72895
C           0.88550       -2.48044        0.14907
C          -1.31384        3.44252        0.00745
C          -1.31810        4.83053        0.23878
C           2.58033       -4.21474       -0.13798
C           1.09926        2.33891        3.48025
C          -0.87653       -1.08484       -0.97270
C          -2.31841        3.85671       -2.73840
C           2.06497       -2.95448       -0.49196
C          -0.18632        5.49900        0.72083
C          -0.12353        2.69087        0.25960
C           4.47405       -1.52174        0.31111
C           0.96363        4.75370        1.00465
C           1.01614        3.36198        0.79225
C           2.29086        2.15771        2.57257
C           2.77419       -2.10132       -1.49297
C           3.93943       -1.39165       -1.09174
C           2.24942        2.64460        1.23838
C           3.46858        1.55303        3.04145
C           1.11375       -2.79785       -3.29144
C           2.27475       -1.96306       -2.81365
C           4.54980       -0.51587       -2.00413
C           3.39512        2.54919        0.40886
C           4.61855        1.44446        2.23590
C           3.36332        3.04318       -1.01481
C           2.90892       -1.05864       -3.68655
C           4.56013        1.95300        0.92750
C           4.03619       -0.31423       -3.29915
C           5.87079        0.79982        2.77528
C           4.67659        0.68555       -4.22881
H          -4.95077       -0.65972        3.55305
H          -4.79876       -1.95296        4.76816
```

H	-3.91081	-0.40927	4.98670
H	-7.14568	1.07342	-0.72304
H	-4.22583	-3.28838	2.51995
H	-1.61003	-0.33444	4.29045
H	-6.97863	1.83335	-2.34241
H	-5.18051	0.93339	0.81750
H	-6.33045	0.19559	-2.04502
H	-3.57717	1.27109	2.50745
H	0.75003	-0.49088	3.97821
H	-1.89799	-5.45385	1.36082
H	-3.46377	-4.76730	0.84577
H	0.33300	-5.10311	2.28075
H	-2.40056	2.62371	2.34756
H	1.47900	-1.82483	3.02256
H	-1.96219	0.98896	1.78830
H	-1.95092	-4.34782	-0.04129
H	0.92350	-0.34349	2.20891
H	-4.77565	2.75793	-3.08324
H	2.36035	-5.98623	1.10280
H	-2.25130	5.38390	0.05099
H	0.88349	3.41593	3.63752
H	0.17948	1.90196	3.04239
H	1.27107	1.87273	4.46916
H	4.65715	-2.57769	0.59104
H	-2.83320	3.88989	-3.71777
H	-0.20822	6.58467	0.89546
H	3.74653	-1.12023	1.04895
H	3.48644	-4.57593	-0.64815
H	-2.13298	4.89876	-2.40948
H	-1.31921	3.39414	-2.88639
H	5.41573	-0.95576	0.43122
H	1.85876	5.24546	1.41661
H	3.49671	1.17494	4.07670
H	0.30159	-2.86662	-2.54329
H	1.44383	-3.84074	-3.48705
H	5.44329	0.04400	-1.68324
H	0.69047	-2.39823	-4.23361
H	2.82838	2.31682	-1.66791
H	2.82994	4.00924	-1.10599
H	5.71232	-0.28162	2.97261
H	6.17996	1.25835	3.73716
H	2.50633	-0.93442	-4.70498
H	4.38538	3.15769	-1.42539
H	5.45270	1.88739	0.28450
H	6.71690	0.89087	2.06680
H	5.77293	0.53136	-4.29957
H	4.52211	1.72250	-3.86089
H	4.25430	0.62617	-5.25046
N	-1.69861	-2.03084	-1.18595
N	0.33238	-1.23285	-0.26297
N	-0.13782	1.29680	0.02689
P	-0.67489	0.72393	-1.62268
P	1.22768	0.29578	-0.58360
C	-2.91384	-1.98330	-2.00225
C	-4.11603	-1.96281	-1.03598
C	-3.01258	-0.78523	-2.96246
C	-2.93101	-3.28795	-2.82583
H	-4.07815	-2.82194	-0.34206
H	-4.10722	-1.03266	-0.43582
H	-5.06712	-2.00926	-1.60498

```
H          -2.12717       -0.72886       -3.63022
H          -3.90933       -0.89162       -3.60614
H          -3.11558        0.17850       -2.42506
H          -3.86693       -3.36656       -3.41493
H          -2.07412       -3.31684       -3.52870
H          -2.85716       -4.16857       -2.15893
```

6.6.14 Adduct 4^{t}Bu$_2$ (PBE-D3/def2-SVP)

```
132
Adduct tBu2
C          -5.27427        0.04268        3.98433
C          -2.78422        0.50518        4.02534
C          -0.32340        1.08817        4.16453
C          -1.72634        3.53782        2.15917
C           3.18339        4.46079        2.98764
C          -3.84510       -0.37517        3.74528
C           0.72487        4.07795        2.49954
C          -1.44364        0.15155        3.78938
C          -0.34179        3.80608        1.62941
C           2.03621        4.27898        2.02672
C          -3.53154       -1.63852        3.21574
C           0.82239       -2.16074        4.28742
C          -1.15631       -1.12188        3.23446
C           2.16492       -2.54874        4.33187
C          -2.20493       -2.03450        2.96129
C           2.24632        4.28163        0.63779
C          -0.09205        3.75199        0.22991
C           0.27145       -1.55801        3.13736
C          -1.97180        4.47942       -1.22959
C          -3.97653        0.40900        0.41783
C           1.19862        4.05077       -0.27498
C          -1.22128        3.41935       -0.68866
C          -3.06882        4.24739       -2.06635
C           2.97626       -2.33416        3.21334
C          -1.89679       -3.42516        2.47065
C           1.09630       -1.36226        1.99928
C           3.86102        0.86246        1.50337
C          -1.57441        2.06866       -0.99221
C          -3.43760        2.92497       -2.33363
C           2.46417       -1.74883        2.03731
C          -4.01093       -0.16086       -0.97353
C          -2.72588        1.82976       -1.80426
C           1.46133        4.16609       -1.75359
C          -4.75029       -1.32664       -1.24012
C          -3.31950        0.47596       -2.03974
C           4.13276       -0.38249        0.69659
C           3.42564       -1.59136        0.89791
C           0.10205       -1.54678       -0.27561
C           1.39483        0.59132       -1.24988
C          -4.82625       -1.88550       -2.52789
C           5.14051       -0.33576       -0.28877
C          -3.37230       -0.07768       -3.34749
C          -5.60347       -3.15350       -2.77453
C           3.75379       -2.74105        0.12389
C           3.06451       -4.05590        0.38014
C          -4.11882       -1.24984       -3.56364
C          -1.99236       -3.34253       -1.54015
```

C	-2.62583	0.56381	-4.48970
C	-0.51372	-3.68145	-1.28948
C	-0.40398	-5.13675	-0.79606
C	5.47120	-1.44948	-1.07332
C	4.75786	-2.64459	-0.85215
C	2.73693	0.73188	-3.26907
C	4.12064	1.34448	-3.53472
C	1.69383	1.36501	-4.20890
C	0.29612	-3.53136	-2.59210
C	6.53649	-1.37661	-2.13829
C	2.77875	-0.79103	-3.47605
H	-5.38637	0.58350	4.94581
H	-3.00355	1.49393	4.46046
H	-0.71042	2.00531	4.64680
H	-1.79312	3.75293	3.24222
H	2.86279	4.97118	3.91743
H	0.53325	4.11401	3.58388
H	-5.95937	-0.82746	3.99595
H	-5.62699	0.73139	3.18609
H	0.38860	0.60260	4.86270
H	4.01013	5.04399	2.53654
H	-2.48822	4.14468	1.63065
H	-2.01402	2.47580	2.00817
H	0.26914	1.39176	3.27531
H	3.60238	3.47498	3.28519
H	0.16916	-2.30930	5.16097
H	2.58073	-3.01096	5.24001
H	-4.34557	-2.35056	3.00190
H	-1.66841	5.50721	-0.97716
H	-4.73338	-0.07221	1.06244
H	-4.15205	1.50378	0.41631
H	3.25724	4.47796	0.24482
H	-3.64412	5.08597	-2.48532
H	-2.98444	0.24487	0.88808
H	-1.42636	-4.03151	3.27415
H	3.19333	0.67746	2.36556
H	4.80659	1.29787	1.88753
H	4.03871	-2.62129	3.23298
H	-2.81525	-3.95099	2.14416
H	3.38590	1.64064	0.86841
H	1.46931	5.23415	-2.06085
H	-5.29258	-1.80752	-0.40934
H	-1.17195	-3.40212	1.63117
H	-4.32899	2.71185	-2.94360
H	2.44312	3.72647	-2.01367
H	0.68234	3.65996	-2.35171
H	-6.55884	-3.16255	-2.21200
H	-2.58422	-3.46356	-0.61185
H	3.38361	-4.49440	1.34901
H	5.69035	0.60847	-0.43281
H	1.96589	-3.91428	0.43731
H	-5.02626	-4.04352	-2.44220
H	-0.94662	-5.26231	0.16129
H	-2.11601	-2.29895	-1.88703
H	-3.03199	1.56251	-4.74863
H	-1.55981	0.72210	-4.22242
H	-5.83345	-3.29168	-3.84910
H	4.09668	2.44101	-3.37560
H	1.66318	2.46504	-4.09213
H	-2.42027	-4.00665	-2.31808

H	3.29567	-4.79096	-0.41554
H	0.65321	-5.42061	-0.62899
H	-4.15003	-1.67841	-4.57880
H	0.68064	0.96320	-4.00632
H	4.86932	0.91454	-2.84307
H	-0.83935	-5.83493	-1.53902
H	-2.66431	-0.06750	-5.39820
H	3.43729	-1.27098	-2.72715
H	0.13836	-2.53278	-3.04987
H	5.00414	-3.53913	-1.44781
H	7.14209	-0.45389	-2.04921
H	1.76944	-1.23388	-3.37768
H	1.37961	-3.66219	-2.39983
H	7.22291	-2.24640	-2.08868
H	4.44505	1.14810	-4.57630
H	1.94787	1.13555	-5.26318
H	-0.02327	-4.28660	-3.33866
H	6.08573	-1.38093	-3.15426
H	3.15256	-1.03183	-4.49166
N	-0.82132	0.96470	-0.52866
N	0.55682	-0.77036	0.80354
N	0.02888	-2.82390	-0.22030
N	2.39404	1.04778	-1.85856
P	0.67717	0.97206	0.47564
P	-0.29049	-0.31564	-1.68404

7 References

[1] C. B. Fischer, S. Xu, H. Zipse, *Chem. Eur. J.* **2006**, *12*, 5779–5784.

[2] F. Reiß, A. Schulz, A. Villinger, N. Weding, *Dalton Trans.* **2010**, *39*, 9962–9972.

[3] J. Bresien, C. Hering-Junghans, A. Schulz, M. Thomas, A. Villinger, *Organometallics* **2018**, DOI: acs.organomet.8b00318.

[4] G. M. Sheldrick, *Acta Cryst. A* **2015**, *71*, 3–8.

[5] G. M. Sheldrick, *Acta Cryst. C* **2015**, *71*, 3–8.

[6] G. M. Sheldrick, *SADABS Version 2*, University of Göttingen, Germany, **2004**.

[7] D. Kratzert, J. J. Holstein, I. Krossing, *J. Appl. Crystallogr.* **2015**, *48*, 933–938.

[8] A. Hinz, A. Schulz, A. Villinger, *J. Am. Chem. Soc.* **2015**, *137*, 9953–9962.

[9] T. Beweries, R. Kuzora, U. Rosenthal, A. Schulz, A. Villinger, *Angew. Chem. Int. Ed.* **2011**, *50*, 8974–8978.

[10] A. Hinz, R. Kuzora, U. Rosenthal, A. Schulz, A. Villinger, *Chem. Eur. J.* **2014**, *20*, 14659–14673.

[11] A. Hinz, A. Schulz, A. Villinger, *Angew. Chem. Int. Ed.* **2015**, *54*, 2776–2779.

[12] C. Feldmeier, H. Bartling, E. Riedle, R. M. Gschwind, *J. Magn. Reson.* **2013**, *232*, 39–44.

[13] *Gaussian 09, Revision E.01*, M. J. Frisch, G. W. Trucks, H. B. Schlegel, G. E. Scuseria, M. A. Robb, J. R. Cheeseman, G. Scalmani, V. Barone, B. Mennucci, G. A. Peterson, H. Nakatsuji, M. Caricato, X. Li, H. P. Hratchian, A. F. Izmaylov, J. Bloino, G. Zheng, J. L. Sonnenberg, M. Hada, M. Ehara, K. Toyota, R. Fukuda, J. Hasegawa, M. Ishida, T. Nakajima, Y. Honda, O. Kitao, H. Nakai, T. Vreven, J. A. Montgomery Jr., J. E. Peralta, F. Ogliaro, M. Bearpark, J. J. Heyd, E. Brothers, K. N. Kudin, V. N. Staroverov, T. Keith, R. Kobayashi, J. Normand, K. Raghavachari, A. Rendell, J. C. Burant, S. S. Iyengar, J. Tomasi, M. Cossi, N. Rega, J. M. Millam, M. Klene, J. E. Know, J. B. Cross, V. Bakken, C. Adamo, J. Jaramillo, R. Gomperts, R. E. Stratmann, O. Yazyev, A. J. Austin, R. Cammi, C. Pomelli, J. W. Ochterski, R. L. Martin, K. Morokuma, V. G. Zakrezewski, G. A. Voth, P. Salvador, J. J. Dannenberg, S. Dapprich, A. D. Daniels, O. Farkas, J. B. Foresman, J. V Ortiz, J. Cioslowski and D. J. Fox, Gaussian, Inc., Wallingford CT, **2013**.

[14] F. Neese, *WIREs Comput. Mol. Sci.* **2012**, *2*, 73–78.

[15] F. Neese, *WIREs Comput. Mol. Sci.* **2018**, *8*, e1327.

[16] E. D. Glendening, J. K. Badenhoop, A. E. Reed, J. E. Carpenter, J. A. Bohmann, C. M. Morales, C. R. Landis, F. Weinhold, *NBO 6.0*, Theoretical Chemistry Institute, University of Wisconsin, Madison, **2013**.

[17] J. E. Carpenter, F. Weinhold, *J. Mol. Struct.: THEOCHEM* **1988**, *169*, 41–62.

[18] F. Weinhold, J. E. Carpenter, *The Structure of Small Molecules and Ions*, Plenum Press, **1988**.

[19] F. Weinhold, C. R. Landis, *Valency and Bonding. A Natural Bond Orbital Donor-Acceptor Perspective*, Cambridge University Press, **2005**.

[20] D. Hegarty, M. A. Robb, *Mol. Phys.* **1979**, *38*, 1795–1812.

[21] R. H. A. Eade, M. A. Robb, *Chem. Phys. Lett.* **1981**, *83*, 362–368.

[22] H. B. Schlegel, M. A. Robb, *Chem. Phys. Lett.* **1982**, *93*, 43–46.

[23] F. Bernardi, A. Bottoni, J. J. W. McDouall, M. A. Robb, H. B. Schlegel, *Faraday Symp. Chem. Soc.* **1984**, *19*, 137.

[24] P. E. M. Siegbahn, *Chem. Phys. Lett.* **1984**, *109*, 417–423.

[25] M. A. Robb, U. Niazi, in *Reports in Molecular Theory, Vol. 1* (Eds.: H. Weinstein, G. Náray-Szabó), CRC Press, Boca Raton, FL, **1990**, pp. 23–55.

[26] M. Frisch, I. N. Ragazos, M. A. Robb, H. B. Schlegel, *Chem. Phys. Lett.* **1992**, *189*, 524–528.

[27] N. Yamamoto, T. Vreven, M. A. Robb, M. J. Frisch, H. B. Schlegel, *Chem. Phys. Lett.* **1996**, *250*, 373–378.

[28] M. Klene, M. A. Robb, M. J. Frisch, P. Celani, *J. Chem. Phys.* **2000**, *113*, 5653–5665.

[29] C. J. Cramer, B. A. Smith, *J. Phys. Chem.* **1996**, *100*, 9664–9670.

[30] C. J. Cramer, *Essentials of Computational Chemistry: Theories and Models*, John Wiley & Sons, Ltd, Chichester, UK, **2004**.

[31] F. London, *J. Phys. Radium* **1937**, *8*, 397–409.

[32] R. McWeeny, *Phys. Rev.* **1962**, *126*, 1028–1034.

[33] R. Ditchfield, *Mol. Phys.* **1974**, *27*, 789–807.

[34] K. Wolinski, J. F. Hinton, P. Pulay, *J. Am. Chem. Soc.* **1990**, *112*, 8251–8260.

[35] J. R. Cheeseman, G. W. Trucks, T. A. Keith, M. J. Frisch, *J. Chem. Phys.* **1996**, *104*, 5497–5509.

[36] S. Grimme, J. Antony, S. Ehrlich, H. Krieg, *J. Chem. Phys.* **2010**, *132*, 154104.

[37] S. Grimme, S. Ehrlich, L. Goerigk, *J. Comput. Chem.* **2011**, *32*, 1456–1465.

[38] F. Weigend, R. Ahlrichs, *Phys. Chem. Chem. Phys.* **2005**, *7*, 3297–305.

[39] A. D. Boese, N. C. Handy, *J. Chem. Phys.* **2002**, *116*, 9559–9569.

[40] T. Van Voorhis, G. E. Scuseria, *J. Chem. Phys.* **1998**, *109*, 400–410.

[41] S. H. Vosko, L. Wilk, M. Nusair, *Can. J. Phys.* **1980**, *58*, 1200–1211.

[42] A. D. Becke, *Phys. Rev. A* **1988**, *38*, 3098–3100.

[43] C. Lee, W. Yang, R. G. Parr, *Phys. Rev. B* **1988**, *37*, 785–789.

[44] B. Miehlich, A. Savin, H. Stoll, H. Preuss, *Chem. Phys. Lett.* **1989**, *157*, 200–206.

[45] A. D. Becke, *J. Chem. Phys.* **1993**, *98*, 5648–5652.

[46] P. J. Stephens, F. J. Devlin, C. F. Chabalowski, M. J. Frisch, *J. Phys. Chem.* **1994**, *98*, 11623–11627.

[47] E. Miliordos, K. Ruedenberg, S. S. Xantheas, *Angew. Chem. Int. Ed.* **2013**, *52*, 5736–5739.

[48] C. J. Jameson, A. De Dios, A. Keith Jameson, *Chem. Phys. Lett.* **1990**, *167*, 575–582.

[49] C. van Wüllen, *Phys. Chem. Chem. Phys.* **2000**, *2*, 2137–2144.
